# Cyanostyryl‐Guanidiniocarbonyl‐Pyrrole Amphiphiles: From Aggregation‐Induced Emission to Photodimerization, Self‐Assembly, and Bioimaging

**DOI:** 10.1002/cplu.202500542

**Published:** 2025-09-23

**Authors:** Kevin Rudolph, Lea Höfmann, Sidharth Thulaseedharan Nair Sailaja, Alexander Höing, Johannes Koch, Nina Schulze, Elisabeth Verheggen, Felix. C. Niemeyer, Florian Uteschil, Shirley K. Knauer, Jens Voskuhl

**Affiliations:** ^1^ Faculty of Chemistry (Organic Chemistry) Center of Medical Biotechnology (ZMB) and Center for Nanointegration (CENIDE) University of Duisburg‐Essen Universitätsstraße 7 45117 Essen Germany; ^2^ Department of Molecular Biology II Center of Medical Biotechnology (ZMB) University of Duisburg‐Essen Universitätsstraße 2 45141 Essen Germany; ^3^ Imaging Center Campus Essen (ICCE) Center of Medical Biotechnology (ZMB) University of Duisburg‐Essen Universitätsstraße 2 45141 Essen Germany

**Keywords:** aggregation‐induced‐emission, amphiphiles, bio‐imaging, self‐assembly, static quenching

## Abstract

Two Cyanostyryl‐guanidiniocarbonyl‐pyrrole based amphiphiles are synthesized and examined in detail. In addition to achieving aggregation‐induced emission from self‐assembly, resulting in nanoparticles, it was found that the observed [2 + 2] photocycloaddition tunes the photophysical properties. The guanidiniocarbonyl‐pyrrole component of these hybrid luminophores is shown to bind oxo‐anions, such as pyrene‐tetracarboxylate, as confirmed by fluorescence lifetime measurements. Moreover, both amphiphiles are used in bio‐imaging experiments with HeLa cells, demonstrating effective cellular uptake.

## Graphical abstract

Amphiphiles with AIE properties, built on a novel luminophore containing an oxo‐anion binding motif, were synthesized and studied for self‐assembly in water. Carboxylate binding was verified by static quenching of a bound pyrene probe, and morphologies were analyzed by TEM, DLS, and ζ‐potential. Despite strong emission, UV light induced photodimerization, modifying photophysical properties and biocompatibility.

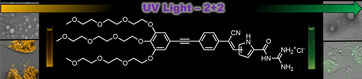

## 1 Introduction

Amphiphiles represent a fundamental component of supramolecular chemistry due to their spontaneous self‐assembly.^[^ ^1^, ^2^ ^–^ ^3^ ^]^ The interplay of hydrophilic and lipophilic domains produces molecules that form specific suprastructures depending on the solvent. In this process, the size, shape, and arrangement of the individual domains are crucial to the dominant morphology.^[^ ^4^ ^]^ By controlling these factors, small micelles, vesicles, bilayer membranes, and even cell‐mimicking assemblies can be obtained.^[^ ^5^, ^6^, ^7^, ^8^ ^–^ ^9^ ^]^ This control enables the creation of specialized templates for specific platforms or carriers used for drug delivery.^[^ ^10^, ^11^ ^–^ ^12^ ^]^ Visual tracking of these carriers by luminophores is a valuable methodology to understand ground‐laying principles of assembly and time‐dependent processes distribution.^[^ ^13^, ^14^, ^15^ ^–^ ^16^ ^]^ While the carriers can be loaded with various luminophores located in the cargo or membrane,^[^ ^17^ ^,^ ^18^ ^]^ there is no further evidence of their presence after potential disassembly. Incorporating emissive properties into self‐assembling molecules enables reliable tracking from formation to degradation of these molecules.^[^ ^19^ ^,^ ^20^ ^]^ Classical fluorophores experience a quenching effect at high concentrations, especially as solids or when aggregated in a nonsolvent (aggregation‐caused quenching (ACQ)), whereas aggregation‐induced emitters (AIEgens) become more emissive in the aggregated state.^[^ ^21^ ^]^ This effect has been known for more than a decade and was described as “emission from solid solutions” by G. C. Schmidt.^[^ ^22^ ^]^ Since 2001, this effect has undergone a renaissance, as Tang and coworkers discovered that phenylated siloles emit efficiently when aggregated.^[^ ^23^ ^]^ It is noteworthy that the ground‐lying mechanism in this phenomenon is the hindrance of molecular motion, not aggregation, causing even single molecules to become emitters when entrapped in a sterically demanding environment^[^ ^24^ ^]^ or in a viscous medium.^[^ ^25^ ^]^ Most publications on micellar and vesicle‐forming amphiphiles with AIE properties focus mainly on the well‐known tetraphenylethene (TPE) motif, which typically forms the lipophilic part domain.^[^ ^26^ ^,^ ^27^ ^]^ Balszuweit et al. recently presented a new class of AIEgens, the so‐called cyanostyryl‐guanidiniocarbonyl‐pyrroles (CGCPs), which merge the established binding motif guanidiniocarbonyl‐pyrrole (GCP), initially designed by Schmuck et al. in 1999 as a versatile oxo‐anion binding motif,^[^ ^28^ ^]^ with the well‐known cyanostilbenes able to act as efficient emitters with AIE properties.^[^ ^29^ ^]^ This design enables sophisticated oxo‐anion binding with emission, addressing common problems associated with basic labeling, such as loss of function and increased interference toxicity.^[^ ^30^, ^31^ ^–^ ^32^ ^]^ In this work, we aim to convert these CGCPs into self‐assembling amphiphiles featuring aggregation‐induced emission. These compounds were studied for their self‐assembly in water, photophysical properties, light responsiveness, and potential toward bio‐imaging of cancer cells. Additionally, the anion binding property of the GCP units was studied using pyrene‐tetracarboxylate, where the amphiphile causes static quenching of the pyrene emission.

## 2 Results and Discussion

### 2.1 Synthesis

**Figure 1:**
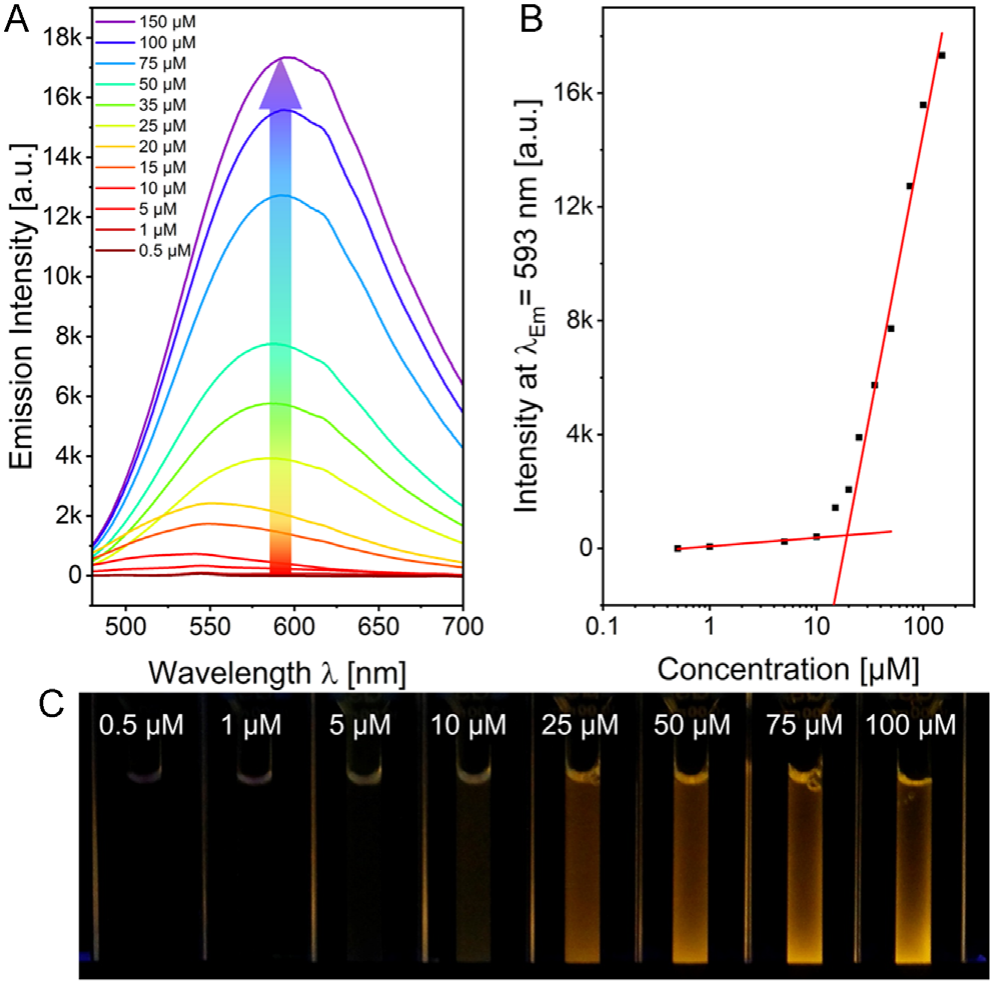
A) Emission spectra of **Aα** in different concentrations with *λ*_Ex_ = 461 nm, B) plotted intensity values at *λ*_Em,max_ = 600 nm against log([**Aα**]) with fitted straights for the first and last four data points each, crossing at the CAC, and C) photograph of cuvettes containing selected concentrations of **Aα** in water under UV‐light irradiation at 365 nm.

**Scheme 1:**
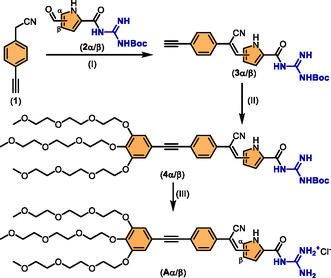
Synthesis of the amphiphiles **Aα** and **Aβ**, (I): K_2_CO_3_, THF, 80 °C, 87% (*α*)/58% (*β*); (II): **5**, Pd(PPh_3_)_4_, CuI, THF, 60 °C, 39% (*α*)/35% (*β*); and (III): 6 M HCl, THF, r.t., quant. yield.

The synthesis of both amphiphiles **Aα** and **Aβ** (α/β indicating the pyrrole substitution pattern) started with the condensation of the CGCP from 4‐ethynyl‐phenylacetonitrile **1** and the corresponding GCP aldehydes **2α**/**β**. The latter were obtained according to known syntheses by Schmuck et al.^[^ ^33^ ^]^ In parallel, the ethylene glycol‐functionalized tail **5** was prepared analogous to Liang et al. and converted to the Boc‐protected amphiphiles **4α**/**β** by Sonogashira coupling (**Scheme** **1** and Scheme S1, Supporting Information).^[^ ^34^ ^]^ The products were obtained as an *E* */* *Z* mixture with respect to the stilbene double‐bond geometry despite the use of the pure *Z*‐isomers of CGCPs **3α**/**β**. Prior to acidic deprotection, an enrichment of the *Z*‐isomers of **4** was obtained by reverse phase‐medium pressure liquid chromatography (MPLC) (RP‐MPLC). It should be noted that, according to Cahn‐Ingold‐Prelog (CIP) nomenclature, the *Z*–configuration of cyanostilbenes refers to both aromatic groups being in *trans*‐geometry because of the higher prioritized nitrile group. Vogeli et al. reported C—H (^3^ *J*_H,C_) coupling constants of around 15 Hz for a *trans*‐geometry of vicinal nitrile‐carbon and proton, while *cis*‐isomers result in lower values.^[^ ^35^ ^]^ After acidic deprotection, the amphiphile **Aα** was obtained as the pure *Z*‐isomer as confirmed in the ^1^H–coupled ^13^C‐NMR (Figure S17, Supporting Information) with a ^3^ *J*_H,C_ coupling constant of 14.2 Hz, indicating the *Z*/*trans*‐configuration. **Aβ** was received in a 4:1 isomer ratio after identical purification (Figure S13, Supporting Information). Additionally, **Aβ** reveals low solubility in most solvents even after neutralization of the guanidinium ion, making it impossible to measure a sufficiently resolved ^1^H–coupled ^13^C‐NMR spectrum to verify the absolute stilbene geometry. However, in comparison to the characterized precursors *E*/*Z‐* **4α** and *E*/*Z*‐**4β**, the downfield shift of the stilbene‐proton at 7.95 ppm suggests the presence of an excess *Z*‐isomer, which was used as such without additional purification (Figure S18, Supporting Information). As a model oxo‐anion for binding to GCP (vide infra), tetra‐sodium pyrene‐1,3,6,8‐tetracarboxyate (**TCP**) was synthesized and employed as the oxo‐anion binding partner for the CGCP amphiphiles. Following the procedure of Zych et al.,^[^ ^36^ ^]^ **TCP** was obtained through nitrile substitution of the respective tetrabromopyrene, followed by base‐catalyzed hydrolysis to yield the carboxylic acid. Owing to the poor solubility, the crude product could not be purified via column chromatography. To facilitate purification by column chromatography, the impure crude mixture was converted into the corresponding butyl esters, purified, and then hydrolyzed under basic conditions to obtain the desired tetra‐substituted product. The amphiphiles were soluble in water at concentrations below 10 μM, whereas visible fluorescence and optical density were observed at higher concentrations (**Figure** **1**C). Since emission from **Aα** and **Aβ** can be attributed to the hindered motion enabled by the AIE effect, this behavior provides an initial insight into the onset of assembly processes.

### 2.2 Self‐Assembly Properties

To investigate the self‐assembly of the amphiphiles, the critical assembly concentrations (CACs) of both amphiphiles were determined by fluorescence spectroscopy on aqueous samples with increasing concentration (Figure 1 & Figure S19, Supporting Information).

Due to spatial restrictions in molecular motion within the assemblies, the aggregation‐induced emission properties can be utilized to monitor the formation of the latter. Once the CAC is exceeded, the samples exhibit a sharp increase in fluorescence intensity at the emission maximum (*λ*_max_) at 600 nm (**Aα**) and 460 nm (**Aβ**). Plotting the intensities of *λ*_max_ against log(concentration), the CAC can be obtained from the intersection point of the two fitted straight lines (Figure 1B). CAC‐values were determined to be 19 ± 2 μM for **Aα** and 32 ± 3 μM for **Aβ**. Assembly‐related experiments were consequently performed at concentrations exceeding the CAC. Both values are within the same order of magnitude but differ sufficiently to the extent that the influence of the pyrrole substitution pattern on the overall molecular geometry or the presence of ≈20% of *E‐* **Aβ** must be considered.

Dynamic light scattering (DLS) measurements were conducted to give insight into the self‐assembly in water (Figure S20, Supporting Information). **Aα** showed sizes of 16–19 nm, whereas the sizes of **Aβ** varied with each measurement, ranging from 30 to 100 nm, indicating less well‐defined assemblies. Given that the DLS method relies on the Stokes–Einstein equation, which assumes spherical structures when calculating the hydrodynamic radius, the reproducible sizes from **Aα** postulate a round morphology that needs to be confirmed by electron microscopy (EM) (vide infra). Though morphology varies for both amphiphiles, the formed assemblies exhibited *ζ*–potential values (Figure S20D, Supporting Information) above 35 mV and can thus be classified as electrostatically stabilized.^[^ ^37^ ^]^ Both samples were irradiated with 405 nm light to induce photoisomerization, enriching the *E*‐CGCP inside the structures and thereby modulating the assemblies’ size and morphology. It was found that an immediate decolorization (yellow to colorless) occurred, and hence, a light–responsiveness was anticipated (Figure S37, Supporting Information). The size distributions determined by DLS differed only slightly from the initial values obtained beforehand, indicating that the visible photoreaction has only a low influence on the formed assemblies. However, a reduction in *ζ*‐potential was observed down to 23 and 26 mV for **A** **α** and **Aβ**, respectively, indicating superficial alterations in the assemblies that lead to the shielding of the positively charged head groups.

**Figure 2:**
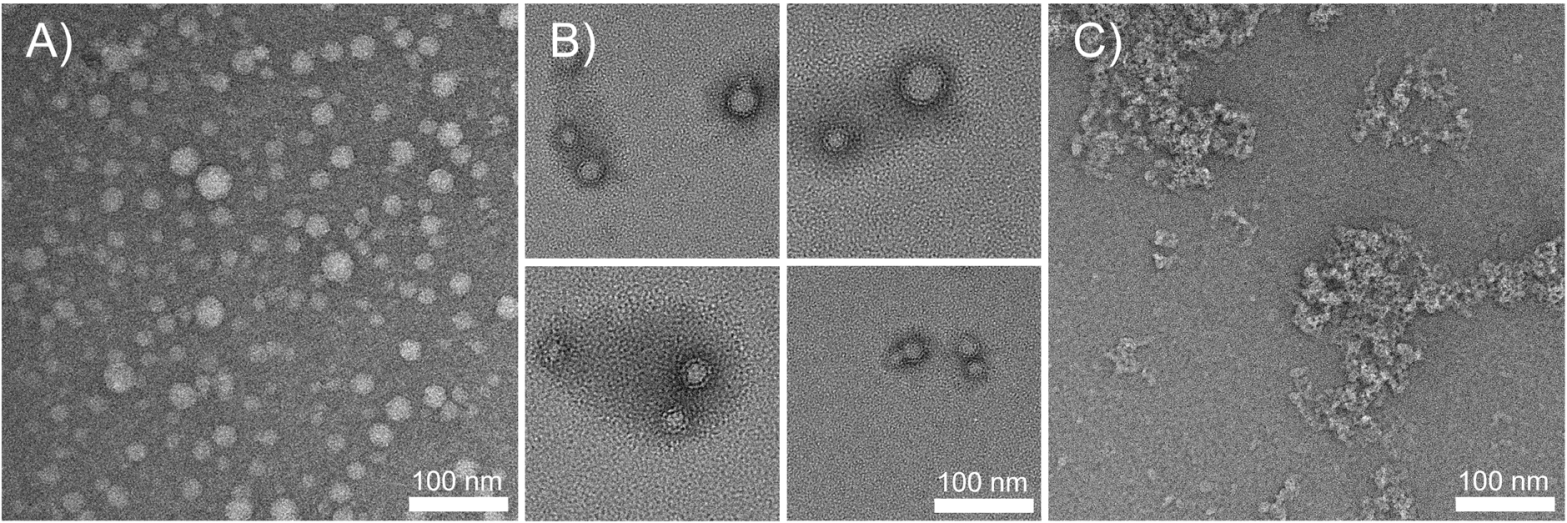
TEM images of A) **Aα** (100 μM), B) **Aα** (100 μM) irradiated with 405 nm prior to sample preparation, and C) a 4:1 mixture of **Aα** (100 μM) and **TCP** (25 μM). Samples are negatively stained with uranyl formate.

The observations from DLS and *ζ*‐potential were verified by transmission electron microscopy (TEM) (**Figure** **2** and Figure S21–26, Supporting Information). Respective samples of **Aα** confirm the presence of round structures measuring between 15 and 32 nm in size, with an average diameter of 20 nm (based on 100 measurements), thereby aligning with the DLS results. **Aβ**, on the other hand, shows structures in the range of 50–100 nm without distinctive geometry (Figure S22, Supporting Information). **Aβ** should have the same tendency to form round assemblies as **Aα**, since both molecules display a similar overall molecular shape. However, **Aα** can be obtained as a pure *Z*‐isomer, while **Aβ** consists of a 4:1 (*Z*:*E*) isomer mixture, which influences its molecular packing. Analogous samples of both amphiphiles, irradiated with 405 nm UV‐light prior to TEM grid preparation (Figure S23, 24, Supporting Information), exhibited similar morphologies and particle diameters compared to the unirradiated starting materials, although an increased contrast at the particles’ edges was observed. Thus, it can be assumed that UV‐light exposure (405 nm) does not induce a morphology change through *Z*/*E*‐isomerization, but it alters the initial superficial structure. Interestingly, the irradiated **Aβ** sample revealed a higher number of spherical structures than its original unirradiated sample, hinting toward a higher geometrical order after the photoreaction. Either a higher amount of *E*‐isomer or the formation of photoproducts with identical geometry from both isomers leads to less distortion. Dünnebacke et al. reported the formation of a [2 + 2] cycloaddition product from cyanostilbenes upon photoreaction in close proximity.^[^ ^38^ ^]^ Although their study focuses only on the *Z‐*isomer, it is postulated that the photoreaction yields only head‐to‐tail products, implying that *E*/*Z* mixtures would produce similarly shaped products in our case.

### 2.3 Photophysical and Chemical Properties

Both amphiphilic structures are related as constitutional isomers, differing only slightly in the pyrrole substitution pattern, which is why they exhibit comparable assembly properties. However, the pyrrole substitution in either *α*‐ or *β*‐position should lead to an overall difference in the photophysical properties of both molecules. **Aα** with two *α*–substituents features a large conjugated *π*‐system that extends from the ethylene glycol‐bearing arene to the guanidinium‐linking carbonyl group. In contrast, **Aβ** with the emission‐inducing cyanostyryl group in *β*–position is crossconjugated to the GCP‐unit.^[^ ^39^ ^]^ Here, the pyrrole unit interrupts the extended *π*‐conjugation, effectively shortening the chromophore compared to **Aα**. As a result, **Aβ** exhibits higher energy transitions, resulting in a blueshifted absorption and emission wavelengths. Therefore, the photophysical properties of both amphiphiles were investigated and compared.

**Figure 3:**
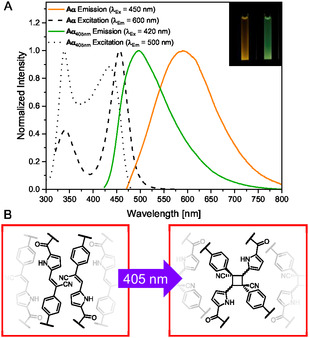
A) Normalized excitation and emission spectra of **Aα** (100 μM) before and after irradiation with 405 nm light for 5 min. The inserted photo shows both samples under UV light. B) Schematic depiction of the occurring photoreaction of **Aα** to **Aα**_**405 nm**_.

The absorption spectra of both **Aα** and **Aβ** are similar, with **Aα** exhibiting a slightly bathochromically shifted absorption maximum compared to **Aβ** (Figure S27, Supporting Information). Similar trends are observed in their emission properties (**Figure** **3**A, Figure S28, Table S2, Supporting Information), which arises through aggregation‐induced emission upon exceeding the CAC. **Aα** displayed a significantly redshifted emission maximum (*λ*_em,max_ = 600 nm) compared to **Aβ** (*λ*_em,max_ = 501 nm). Overall, the substitution pattern on the pyrrole ring strongly influences the absorption and emission properties of the molecules.

The photoproducts obtained after irradiation with 405 nm showed strongly altered photophysical properties, most notably a pronounced reduction in the intensities of the absorption bands. To classify the speed of this reaction, the sample was irradiated under continuous stirring at controlled time intervals, and the absorption maxima (*λ*_abs,max_) were plotted against time (Figures S43, S44, Supporting Information). While the absorption intensity at 392 nm for **Aα** decreases to 10% of its initial value after 180 s of irradiation, whereas **Aβ**, which has its initial strongest maximum at 363 nm reaches the same intensity just after 120 s. Typically, cyanostilbenes undergo *Z*/*E*‐isomerization when exposed to UV light, as indicated by the appearance of a new, slightly hypsochromically shifted maximum in the UV/vis spectrum.^[^ ^38^ ^]^ The absence of this and the observed decrease in all absorption bands suggest that the *π*–conjugated system is disrupted upon irradiation. This molecular change is also evident from the hypsochromically shifted emission maxima of both molecules. For **A** **α**_**405 nm,**_ a 98 nm shift of emission occurs (*λ*_em,max_ = 600 to 504 nm), while the maximum of **Aβ**_**405 nm**_ shifts by 42 nm (*λ*_em,max_ = 501 to 459 nm).

From the above observations and the proven self‐assembly formation, it can be presumed that a [2 + 2]–cycloaddition of the stilbene double bond occurs for both amphiphiles, as reported by others.^[^ ^38^ ^]^ NMR and mass spectra underline this finding by showing the photocyclized proton at 5.87 ppm with a ^1^ *J*_H,C_ and ^2^ *J*_H,C_ correlation toward the newly formed photocyclized carbons at 47.65 and 49.97 ppm, visible in HSQC and HMBC 2D‐spectra, respectively (Figure S39–41, Supporting Information). These data are consistent with literature reports on [2 + 2] photoproducts derived from common cyanostilbenes.^[^ ^40^, ^41^ ^–^ ^42^ ^]^ Additionally, a new twofold positively charged water conjugate could be detected in high‐resolution electrospray ionization time‐of‐flight mass spectrometry (ESI‐TOF‐MS) Figure S42, Supporting Information). By correlating the photophysical changes with the appearance of aliphatic signals in the NMR spectra and a doubled mass after irradiation, the formation of a cyclobutene via [2 + 2] cycloaddition of dimerized amphiphiles can be confirmed (Figure 3B).

### 2.4 Anion‐Binding Properties

**Figure 4:**
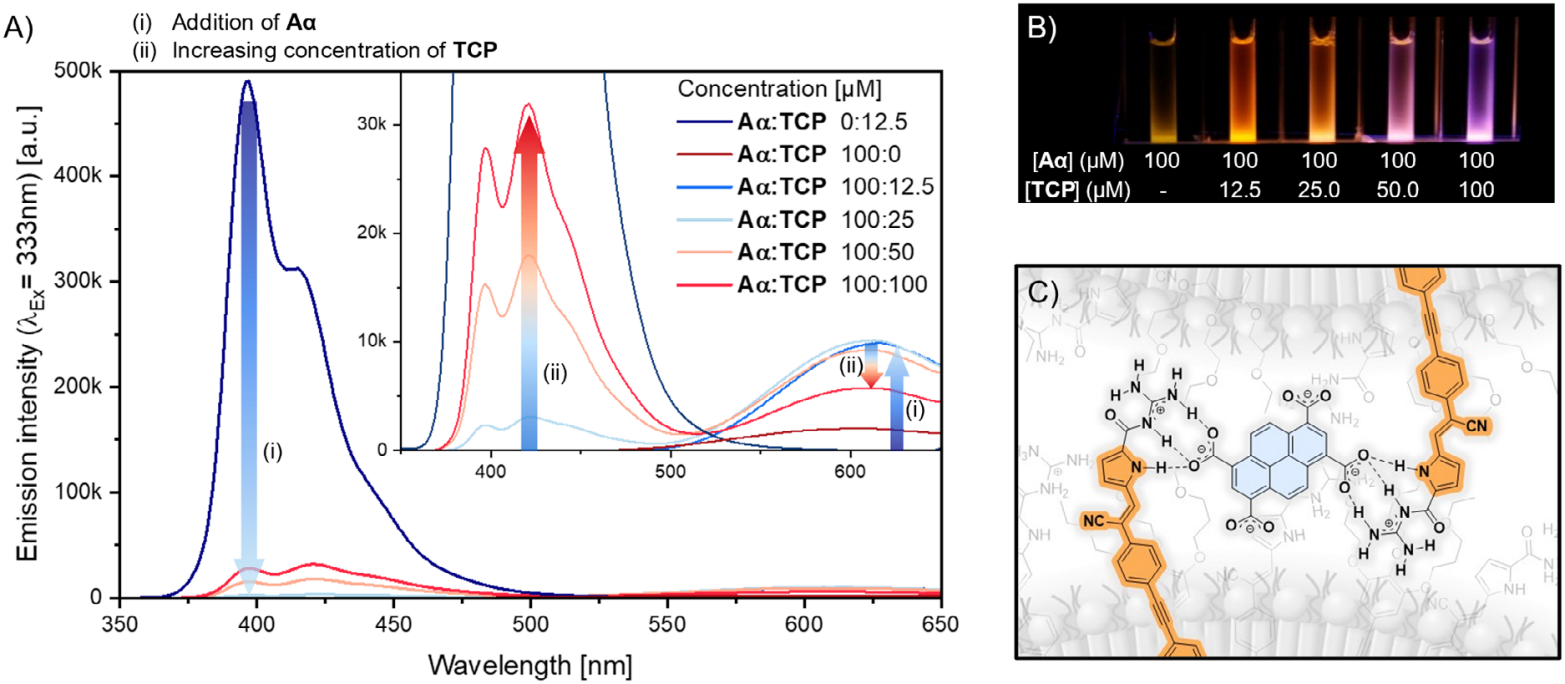
A) Fluorescence emission spectra unimolecular samples of **TCP** and **Aα** (*λ*_ex_ = 333 nm) and different mixtures in water, recorded at a fixed concentration of **Aα** with increasing amounts of **TCP**. The inset shows the expanded view of the spectra. B) Photograph of respective samples of **Aα** with increasing ratio of **TCP** in water under UV light (365 nm). C) Schematic representation of the binding of the GCP units of **Aα** molecules with the oxo‐anion of **TCP** on the micellar surfaces.

To investigate the anion binding of the GCP units incorporated within the amphiphiles, we prepared solutions of **Aα** and **Aβ** at a fixed concentration of 100 μM and measured their emission spectra upon addition of increasing amounts of pyrene‐tetracarboxylate (**TCP**, **Figure** **4**, S29, Supporting Information). **TCP** was chosen as a water‐soluble pyrene derivative to serve as a suitable binding partner, which typically emits in the blue range of visible wavelengths. Typical binding assays, such as ITC or Job‐Plot, rely exclusively on intermolecular interactions in the absence of other competing effects. However, the formation or solvation of assemblies can interfere with these interactions and can potentially lead to the distortion of the obtained data. Steady‐state and time‐resolved photoluminescence spectroscopy are methods that are highly sensitive to environmental changes, and therefore, they serve as an established method for detecting interactions between molecules in close proximity.

To further examine the binding affinity of **TCP** toward the GCP unit in the amphiphiles, we carried out fluorescence emission and lifetime measurements while increasing the concentrations of **Aα**, keeping the **TCP** concentration constant (Table S2, Figure S30A, Supporting Information). The changes observed in the emission spectra of **TCP** with increasing **Aα** concentrations confirm the interaction between the two molecules. **TCP** alone exhibits strong emission at 397 nm; however, upon increasing the concentration of **Aα**, the emission intensity of **TCP** decreases significantly. In order to understand the underlying quenching process, whether dynamic or static, the fluorescence lifetime of **TCP** was measured across varying concentrations of **Aα**, and the data were evaluated using a Stern–Volmer plot (Figure S30B, Supporting Information). Despite the significant decrease in emission intensity, no change in the fluorescence lifetime of **TCP** was observed (Figure S31–36, Supporting Information). This behavior is characteristic of static quenching, wherein fluorescence is reduced due to ground–state complex formation rather than diffusion–controlled quenching.^[^ ^43^ ^,^ ^44^ ^]^ In static quenching, the number of emissive species is reduced through complex formation, while the excited state lifetime remains unchanged. Hence, in our system, the quenching of **TCP** emission by **Aα** can be assigned to a static quenching process, indicative of stable complex formation between **TCP** and the GCP unit (Figure 4C).

Even with **TCP** bearing four carboxylate groups, it is not necessarily the case that one molecule binds multiple CGCP units. Since a complete quenching effect of the **TCP** emission is only observed for an 8:1 ratio, it may be highly possible that due to steric hindrance between multiple amphiphiles and weak interaction toward **TCP** in water, which competes with solvation, free **TCP** is present without an excess of the amphiphiles. As the emission intensity of **TCP** decreases with the addition of **A**α, an enhancement of the emission intensity of **Aα** can be observed (Figure 4A&B). Since static quenching does not necessarily correlate with higher emission efficiency of the quencher, this increase could also be due to denser packing. Because of the 4:1 ratio of binding sites, **TCP** can connect two or more assemblies (Figure 4C), forming agglomerates, as observed in TEM (Figure 2C), which enable the activation of **Aα**'s AIE. As the **TCP** ratio increases further, the emission intensity of **Aα** decreases due to an excess of the oxo‐anion. This unquenched emission strongly influences the overall fluorescence appearance, which at higher **TCP** content is shifted to purple (Figure 4B). At an equimolar ratio, the emission intensity of the amphiphile band decreases again, indicating a loss of spatial limitation and, therefore, possible solvation of the overall assemblies (Figure 4A).

A stoichiometric ratio of 4:1 (concerning binding moieties) already leads to near neutralization of the *ζ*‐potential at around 4 mV (Figure S20D, Supporting Information), drastically decreasing stability. Pyrene derivatives, as well as CGCP, are highly planar.^[^ ^45^ ^]^ Hydrogen‐bonded complexes could result in defined structures from *π*–stacked disc‐like complexes, which is why we were interested in revealing the morphology of the formed structures in 4:1 mixtures. Interestingly, the hydrodynamic diameters measured by DLS varied randomly between 20 and 70 nm, so that no predominant morphology can be assumed. These results agree with the measured *ζ*–potentials of all samples. Without **TCP**, **A** **α** and **Aβ** are stabilized as described before; the mixtures, on the other hand, have values below 10 mV and cannot be classified as stable. TEM measurements of **TCP** amphiphile mixtures reveal no spherical particle morphology, but accumulations of compound (Figure 2C and Figure S25, 26, Supporting Information). The lack of electrostatic stabilization could lead to the aggregation of undefined assemblies, which dry into larger structures.

### 2.5 Bioimaging

**Figure 5:**
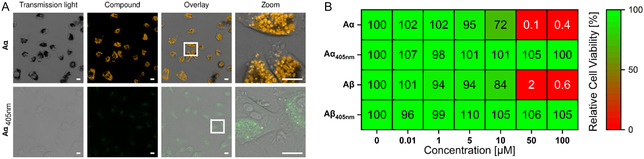
A) Confocal laser scanning microscopy (CLSM) of HeLa cells incubated with **Aα** (10 μM). Scale bar: 20 μm. B) 24 h toxicity study investigating the effect of amphiphiles and photoreacted amphiphiles on cell viability (0.01–100 μM).

To overcome the limitation of water solubility, that most organic molecules impede from bio‐availability, additional solvent is often used to enhance the latter. Another approach, which does not introduce a new variable into such studies, is to induce amphiphilic properties to form stable nanoassemblies in water. The CGCP motif already demonstrates promising properties in terms of cytotoxicity and cellular internalization, as previously reported.^[^ ^29^ ^]^ With the amphiphilic derivatives **Aα** and **Aβ** having a better compatibility with water, we were motivated to test the general biocompatibility in vitro. Balszuweit et al., as well as numerous studies by the Schmuck group targeting the GCP binding motif, utilize the human cervical carcinoma cell line HeLa‐Kyoto as a standard.^[^ ^46^, ^47^, ^48^, ^49^, ^50^, ^51^, ^52^, ^53^, ^54^ ^–^ ^55^ ^]^ For reproducibility and comparison reasons, we also focus on this target to address cytotoxicity and cell permeability of the amphiphiles and their photoproducts. Potential cytotoxic effects of all compounds were investigated by MTS cell proliferation assay (**Figure** **5**B & Figure S45, 46, Supporting Information). While the photoreacted amphiphiles showed no toxic effects, the unirradiated compounds affected cell proliferation in concentrations of 10–50 μM. Notably, the amphiphiles overcame the CAC within the same concentration range. A closer look at relative cell viability plots of the unirradiated compounds revealed IC_50_ values of 12.3 μM for **A** **α** and 20.6 μM for **Aβ**. Again, this trend aligns with the CAC, with **A** **α** having a slightly lower value than **Aβ**. We chose confocal laser scanning microscopy (CLSM) to investigate correlations with the assembly and the cellular uptake at concentrations below and above the CAC. At 10 μM concentration and 24 h incubation time, the amphiphiles **Aα** and **Aβ** show good uptake even though the CAC is not reached. The compounds are located inside vesicular structures in the cytoplasm (Figure 5A & Figure S47, 48, Supporting Information). Automated Image Analysis was performed to extract the average cell size (Figure S49, Supporting Information).^[^ ^56^ ^,^ ^57^ ^]^ Mean values of 1.4 and 1.2 μm^2^ were measured for **A**α and **Aβ**, respectively, while it must be mentioned that a broad distribution was achieved (Figure S42, Supporting Information). Concentrations above the CAC of the compounds only led to an increase in fluorescence, but no change in localization occurred. Thus, we cannot correlate the cell uptake of the amphiphiles **Aα** and **Aβ** with the effect of self‐assembly. The cytotoxicity at higher concentrations remains to be explained, as the bioavailability of the vesicular‐trapped compounds appears to be very low. If single molecules were able to escape and bind to DNA in deeper cell functionalities, a fluorescence signal of such would be expected, as well as higher toxicity.^[^ ^29^ ^]^ However, microscopy reveals why cytotoxicity completely vanishes for the photoreacted compounds, as **Aα**_**405 nm**_ shows significantly weaker cell uptake at both concentrations compared to its unaltered starting compound. **Aβ**_**405 nm**_ seems to be unable to enter the cells at all. At higher concentrations, big aggregates of **Aβ**_**405 nm**_ are only found in the medium. Since the cell‐uptake for the nonirradiated amphiphiles is independent of the formed morphologies, the explanation for the deviant behavior of the photoproducts must be due to the different molecular structure. With the cyclobutane core of **Aα**_**405 nm**_ and **Aβ**_**405 nm**_ bringing volume to the originally planar amphiphiles, the internalization could be obstructed. Moreover, the GCP binding motif may be sterically hindered from interacting with negative cell‐surface charges. Considering the assumed head‐to‐tail geometry of the formed dimer,^[^ ^38^ ^]^ and more evidently the decrease in *ζ*‐potential of irradiated assemblies, the GCP‐motif is possibly shielded by the neighboring ethylene glycol chains and therefore cannot efficiently attach to the cells. It cannot be excluded that the emission visible for **Aα**_**405 nm**_ derives from nonreacted amphiphile, but that would raise the question of why **Aβ**_**405 nm**_ does not show the same tendency. It is more plausible that the varying orientation of the GCP‐motif imposes the different cell‐uptake behavior of both photoproducts. In total, nonirradiated amphiphiles and photoproducts differ in structure, geometry, and orientation of the functional groups, which is why bio‐compatibility could vary.

## 3 Conclusion

To conclude, we synthesized two amphiphiles, **Aα** and **Aβ**, based on the CGCP‐AIE motif, which form assemblies in water. Both are regioisomeric amphiphiles, differing in their pyrrole‐substitution patterns, and exhibit unique photophysical (aggregation–induced emission) and self‐assembly properties. DLS measurements and TEM images show the formation of defined round structures from **Aα**, while **Aβ** assembles into shapes with no predominant geometry, possibly due to a mixture of *E*‐ and *Z*‐isomers. Upon mixing the assemblies with the luminescent tetra‐anionic pyrene‐tetracarboxylate (**TCP**), employed as a model binding substrate for the GCP unit, a disruption of the well‐defined structures of **Aα** was observed, accompanied by static fluorescence quenching. This confirms the formation of a specific binding interaction between **TCP** and the GCP‐containing amphiphile. The irradiation with 405 nm light initiated the well‐known [2 + 2] photocyclization between two neighboring amphiphiles, yielding a cyclobutene moiety. The photodimers of **A** **α** were characterized using NMR spectrometry and mass spectroscopy.

The photoproducts of **A** **α** and **Aβ** exhibit a drastic change in photophysical properties, characterized by hypsochromically shifted absorption and fluorescence bands. The morphology and size of the assemblies, however, seem almost unaltered, as proven by TEM and *ζ*‐potential, although an increased density in the membrane was well detectable. Finally, the biocompatibility and cellular distribution of both amphiphiles and their respective photoproducts were investigated in detail using HeLa Kyoto cells. Before photoirradiation, the amphiphiles show good cellular uptake but become cytotoxic at concentrations exceeding the CAC. The photoproducts neither show significant toxicity nor are they taken up by the HeLa cells, even at high concentrations. Besides that, the assembled amphiphiles of **Aα** and **Aβ** were located inside vesicular structures, as proven by CLSM. This design concept—merging luminophores with binding motifs while converting into amphiphilic structures will for sure open new avenues toward novel classes of potential cargo delivery systems, which can be easily tracked by fluorescence‐lifetime imaging microscopy (FLIM).

## Supporting Information

The authors have cited additional references within the Supporting Information.^[33–36, 56–61]^

## Conflict of Interest

The authors declare no conflict of interest.

## Supporting information

Supplementary Material

## Data Availability

The data that support the findings of this study are available in the supplementary material of this article.
