# Supplementary material for: Cyanostyryl‐Guanidiniocarbonyl‐Pyrrole Amphiphiles: From Aggregation‐Induced Emission to Photodimerization, Self‐Assembly, and Bioimaging
Source: Chempluschem. 2025 Sep 23;90(12):e202500542. doi: 10.1002/cplu.202500542 (PMC12701296; doi:10.1002/cplu.202500542)
Supplement: Supplementary file 1 — Supplementary Material [file CPLU-90-e202500542-s001.pdf]

Cyanostyryl-guanidiniocarbonyl-pyrrole amphiphiles: From aggregation-induced emission to photodimerization, self-assembly and bioimaging

Kevin Rudolph,[a]+ Lea Höfmann,[a]+ Sidharth Thulaseedharan Nair Sailaja,[a]+ Alexander Höing,[b] Johannes Koch,[c] Nina Schulze,[c] Elisabeth Verheggen,[a] Felix. C. Niemeyer,[a] Florian Uteschil,[a] Shirley K. Knauer[b] and Jens Voskuhl*[a]

[a] K. Rudolph, L. Höfmann, S. T. Nair Sailaja, E. Verheggen, Dr. F. C. Niemeyer, Dr. F. Uteschil, Prof. Dr. J. Voskuhl*
Faculty of Chemistry (Organic Chemistry, Center of Medical Biotechnology (ZMB) and Center for NanoIntegration (CENIDE)
University of Duisburg-Essen
Universitätsstraße 7, 45117 Essen, Germany
* Corresponding author: E-mail: jens.voskuhl@uni-due.de.
+ authors contributed equally.

[b] Dr. A. Höing, Prof. Dr. S. K. Knauer
Department of Molecular Biology II, Center of Medical Biotechnology (ZMB)
University of Duisburg-Essen
Universitätsstraße 2, 45141 Essen, Germany

[c] Dr. J. Koch, Dr. N. Schulze
Imaging Center Campus Essen (ICCE), Center of Medical Biotechnology (ZMB)
University of Duisburg-Essen
Universitätsstraße 2, 45141 Essen, Germany

1. General Information

Chemicals were purchased from Sigma Aldrich or TCI Chemicals and used without further purification if not otherwise noted. Reactions were carried out in the dark, using dried solvents and under an atmosphere of argon. Dried DMF (extra dry) was purchased from *Acros Organics*, dried THF was freshly distilled from Na. MQ-water (R = 18.2 Ω) from an *Elga Purelab* classic was used for stock solutions. Reactions were monitored by thin-layer chromatography (TLC), using 0.2 mm POLYGRAM SIL G/UV254 plates by *Macherey-Nagel*. Spots were visualized by basic $KMnO_4$ solution, acidic 2,4-dinitrophenylhydrazine or under a UV lamp (254, 365, and 395 nm). Column chromatography was carried out on silica gel 60 (0.04 – 0.063 mm) by *Macherey-Nagel*. Medium-pressure column chromatography (MPLC) was carried out on a *Gilson* PLC2050 using self-packed RP-18 columns with 17 g or 120 g fill-weight of LiChroprep® RP-18 (0.040 – 0.063 mm) by *Merck*. NMR spectra were recorded on a *Bruker* Avneo 400 MHz spectrometer (^{1}H: 400 MHz, ^{13}C: 101 MHz) or a *Bruker* Avance III HD 600 MHz (^{1}H: 600 MHz, ^{13}C: 151 MHz). All measurements were performed at room temperature (r.t.), using Acetone-d_6 or DMSO-d_6 as solvents. The chemical shifts are referenced relative to the residual proton signals of the solvents in the ^{1}H-NMR spectrum (Acetone-d_6: δ = 2.05 ppm, DMSO-d_6: δ = 2.50 ppm) or relative to the solvent signal in the ^{13}C-NMR spectra (Acetone-d_6: δ = 29.84 ppm, DMSO-d_6 δ = 39.51 ppm). Coupling constants (*J*) are reported in Hertz (Hz). Mass spectra were recorded using a LC-MS system equipped with a liquid chromatograph from *Agilent Technologies* (1260 Infinity) and a high-resolution time-of-flight mass spectrometer from *Bruker Daltonics* (Maxis 4G), employing electrospray ionization (ESI) in positive mode as the ionization source. A YMC Triart C18 (50x1 mm I.D. with 3 µm particle size) was used as an analytical column. The mobile phase consisted of water with 0.1% formic acid (A) and methanol with 0.1% formic acid (B) at a flow rate of 100 µL min^{-1}. The gradient was established as follows: 0 min, 10% B, 30 min 100% B and was maintained for 30 min. The eluent was introduced into the mass spectrometer's ion source. Nitrogen was used as a nebulizing gas and a dry gas. The nebulizer gas pressure was set to 29.0 psi, and the dry gas was set to 5.0 L min^{-1} at 250 °C. The capillary voltage was set to -4500 V. The end plate offset was set to -500 V. The acquisition was performed in scan mode over the m/z range of 100 – 2900. The MS system was controlled by the software Hystar version 3.2. The data was analysed using Compass Data Analysis version 4.1. UV/vis- and fluorescence spectra were measured on a *Jasco* V-550 and *Shimadzu* RF-6000, respectively, using semi-micro quartz-glass fluorescence cuvettes with 4x10 mm lightpath and 1.4 mL volume from *Hellma Analytics*. DLS and ζ-potential measurements were performed on a *Malvern* Zetasizer nanoZS using disposable small-volume PMMA cuvettes and DTS1070 cells, respectively, as provided by *Malvern*. Photographs taken under UV light were captured in the dark using a *Canon* EOS 1100D camera, with the exposure time adjusted to match the optical impression, and a 365 nm Lamp by *Herolab*. IR spectra were measured on a *Jasco* FT/IR-4600. TEM measurements were performed on a *JEOL* JEM-2200FS microscope using an acceleration voltage of 200 kV. Samples were negatively stained with uranyl formate. Irradiation experiments were conducted using a Star-UV405-03-00-00 High-Power UV LED (405 nm) by *Roschwege* with a 700 mA power source. Stock solutions of **Aα**, **Aβ**, and **TCP** with 10 mM concentration in MQ-water were prepared prior the experiments and kept in the cold and dark. Lifetime measurements of **Aα**, **TCP**, and mixtures were examined using the FluoTime 300 from *Picoquant*, with excitation at 373 nm. Quantum yields were determined with the *Hamamatsu* Quantaurus QY at room temperature.

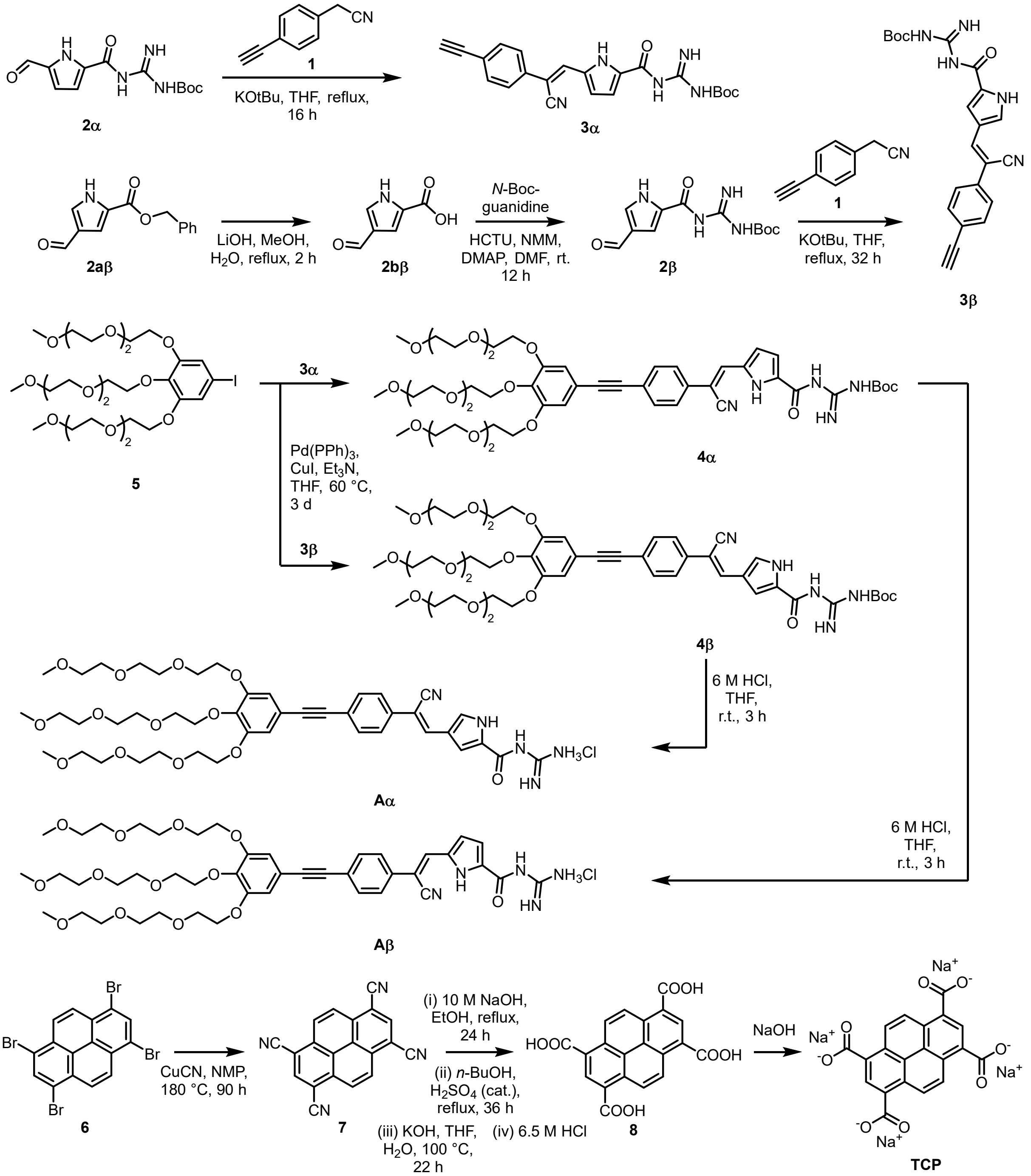

Scheme 1: Synthesis route of **Aα**, **Aβ** and **TCP**.

The following compounds have been described in the literature and synthesized according to the corresponding procedures: **2α**[1], **2aβ**[2] and **5**[3]. The analytical details were in accordance with those reported.

2. Synthetic procedures

4-Formyl-1H-pyrrol-2-carboxylic acid (2bβ)

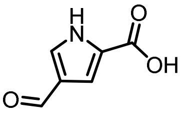

Benzyl 4-Formyl-1H-pyrrol-2-carboxylate[2] (**2aβ**) (5.10 g, 22.3 mmol, 1.0 eq.) and LiOH (1.40 g, 58.5 mmol, 2.6 eq.) were dissolved in 75 mL MeOH and 25 mL water. The reaction was stirred at 90 °C for 2 h. After removal of MeOH by evaporation, the aqueous residue was adjusted to pH = 1 by addition of 6.5 M HCl, yielding a brown-orange solid. After filtration, the brown solid was washed three times with 3 mL of cold water. After drying, a light brown solid was obtained (3.0 g, 21.7 mmol, 98%). Molar mass: 139.11 g/mol, Molecular formula: $C_6H_5N_1O_3$, ^{1}H-NMR (400 MHz, DMSO-d_6) δ = 12.81 (s (br), 1H), 12.53 (s, 1H), 9.74 (s, 1H), 7.76 (dd, *J* = 3.4, 1.6 Hz, 1H), 7.07 (dd, *J* = 2.4, 1.6 Hz, 1H) ppm. ^{13}C-NMR (101 MHz, DMSO-d_6) δ = 185.91, 161.60, 130.91, 126.66, 125.66, 112.68 ppm. IR (ATR): 3296, 3166, 3119, 2878, 1682, 1645, 1566, 1452, 1435, 1416, 1383, 1353, 1338, 1260, 1212, 1141, 1119, 869, 779, 733, 608, 558 cm^{-1}. HR-MS (ESI-pos.), m/z: 140.0345 (calcd. 140.0342 for $[C_6H_6N_1O_3]^+$).

1-(4-Formyl-1H-pyrrol-2-carbonyl)-*N*-Boc-guanidin (2β)

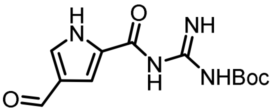

2bβ (3.0 g, 21.7 mmol, 1.0 eq.), HCTU (10.5 g, 25.3 mmol, 1.2 eq.), NMM (12.1 mL, 55.2 mmol, 2.5 eq.) and 20 mg DMAP were dissolved in 130 mL DMF and stirred for 1 h at r.t. before addition of *N*-Boc-guanidine (5.46 g, 34.3 mmol, 1.6 eq.). The reaction was stirred for 12 h before it was poured into 380 mL of ice water. The resulting light brown precipitate was filtered and dried. The crude product was purified using column chromatography (SiO_2, DCM : ethyl acetate 6:1 → 2:1) and subsequently RP-MPLC (RP18, MeOH : H_2O 30:70 → 100:0) to give an off-white solid (0.9 g, 3.30 mmol, 15%). Molar mass: 280.28 g/mol, Molecular formula: $C_{12}H_{16}N_4O_4$, ^{1}H-NMR (400 MHz, DMSO-d_6) δ = 12.21 (s, 1H), 10.88 (s, 1H), 9.72 (s, 1H), 9.29 (s, 1H), 8.54 (s, 1H), 7.73 (s, 1H), 7.18 (s, 1H), 1.46 (s, 9H) ppm. ^{13}C-NMR (151 MHz, DMSO-d_6) δ = 185.94, 170.02 (br), 158.49, 153.79 (br), 132.08 (br), 130.88, 126.58, 111.26, 81.33, 27.78 ppm. IR (ATR): 3380, 3182, 2981, 1733, 1720, 1670, 1634, 1551, 1497, 1474, 1433, 1413, 1392, 1366, 1283, 1263, 1237, 1227, 1095, 1059, 999, 940, 873, 846, 789, 763, 618, 536, 502, 485, 474, 457, 445, 404 cm^{-1}. HR-MS (ESI-pos.), m/z: 281.1243 (calcd. 281.1244 for $[C_{12}H_{17}N_4O_4]^+$).

4-Ethynylcyanostyryl-α-guanidiniocarbonyl(boc)guanidine (3α)

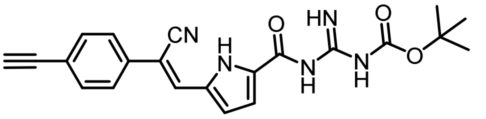

4-Ethynylphenylacetonitrile (**1**) (1.2 mL, 8.82 mmol, 2.0 eq.), 1-(5-Formyl-1H-pyrrol-2-carbonyl)-N-Boc-guanidine (**2α**) (1.2 g, 4.28 mmol, 1.0 eq.) and KO*t*Bu (1.2 g, 10.7 mmol, 2.5 eq.) were suspended in 80 mL THF and refluxed for 16 h. After cooling, the reaction was mixed with water and extracted with ethyl acetate five times, using brine to enhance phase separation. The combined organic phases were dried over $MgSO_4$, filtered, and the solvent was removed under vacuum. The crude product was purified using column chromatography (SiO_2, 9:1 DCM : ethyl acetate), yielding a yellow-orange solid (1.49 g, 3.71 mmol, 87%). Molar mass: 403.44 g/mol, Molecular formula: $C_{22}H_{21}N_5O_3$, ^{1}H-NMR (400 MHz, Acetone-d_6) δ = 11.00 (s (br), 1H), 10.25 (s (br), 1H), 9.25 (s (br), 1H), 8.55 (s, 1H), 7.95 (s, 1H), 7.69 (m, 2H), 7.60 (m, 2H), 7.24 (d, *J* = 4.09, 1.88, 1H), 6.55 (d, *J* = 4.12, 1.59, 1H), 3.79 (s, 1H), 1.52 (s, 9H) ppm. ^{13}C-NMR (101 MHz, Acetone-d_6) δ = 159.8, 154.6, 135.6, 133.8, 133.6, 132.6, 131.1, 129.9, 126.0, 123.2, 119.1, 116.0, 115.5, 105.4, 83.7, 83.4, 80.9, 28.1 ppm. IR (ATR): 3370, 3283, 2976, 2930, 2220, 2162, 2102, 1715, 1614, 1593, 1553, 1520, 1506, 1464, 1429, 1414, 1395, 1368, 1339, 1279, 1267,

1238, 1144, 1090, 1067, 1045, 1013, 984, 966, 926, 907, 882, 835, 773, 756, 729, 660, 640, 623 cm^{-1}. HR-MS (ESI-pos.), m/z: 404.1716 (calcd. 404.1717 for $[C_{22}H_{22}N_5O_3]^+$).

4-Ethynylcyanostyryl-β-guanidiniocarbonyl(boc)guanidine (3β)

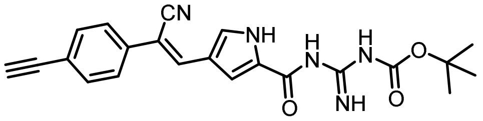

4-Ethynylphenylacetonitrile (**1**) (244 mg, 1.73 mmol, 1.5 eq.), 1-(4-Formyl-1H-pyrrol-2-carbonyl)-N-Boc-guanidine (**2β**) (323 mg, 1.15 mmol, 1.0 eq.) and KO*t*Bu (194 mg, 1.73 mmol, 1.5 eq.) were suspended in 10 mL THF and refluxed for 16 h. Since the reaction was not complete, an additional 0.7 eq. of **1** and KO*t*Bu were added, and the reaction was again left to reflux for 16 h. This process was repeated until no more aldehyde (**2β**) could be observed on TLC (R_f = 0.25, DCM : Acetone 9:1). After cooling, the reaction was mixed with water (50 mL), the solvent evaporated, and the aqueous residue extracted with ethyl acetate three times. Combined organic phases were dried over $MgSO_4$, filtered, and solvent removed under vacuum. The crude product was purified using column chromatography (SiO_2, DCM : ethyl acetate 19:1 → 2:1), yielding a light yellow solid (270 mg, 0.67 mmol, 58%). Molar mass: 403.44 g/mol, Molecular formula: $C_{22}H_{21}N_5O_3$, ^{1}H-NMR (400 MHz, Acetone-d_6) δ = 11.11 (s, 1H), 10.44 (s, 1H), 9.26 (s, 1H), 8.52 (s, 1H), 7.89 (s, 1H), 7.69 (d, *J* = 8.5 Hz, 2H), 7.66 (s, 2H), 7.62 (s, 1H), 7.57 (d, *J* = 8.5 Hz, 2H), 3.75 (s, 1H), 1.50 (s, 9H) ppm. ^{13}C-NMR (101 MHz, Acetone-d_6) δ = 159.84, 138.39, 136.30, 133.98, 133.63, 133.41, 129.91, 128.33, 125.89, 122.59, 121.61, 119.40, 112.86, 104.60, 83.79, 83.26, 80.43, 28.12 ppm. IR (ATR): 3368, 3261, 2976, 2336, 2207, 2105, 1726, 1626, 1594, 1528, 1506, 1479, 1390, 1367, 1286, 1236, 1142, 1002, 959, 836, 776, 753, 639, 594, 554, 529, 512, 483, 474, 457, 436, 420, 403 cm^{-1}. HR-MS (ESI-pos.), m/z: 404.1718 (calcd. 404.1717 for $[C_{22}H_{22}N_5O_3]^+$).

Boc-α-Amphiphile (4α)

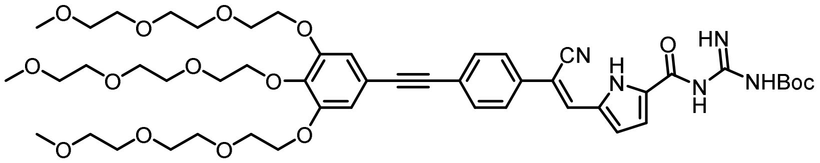

5[3] (159 mg, 0.23 mmol, 1.0 eq.) was added to 2 mL THF via syringe before addition of **3α** (95 mg, 0.23 mmol, 1.0 eq.), CuI (1.5 mg, 3 mol%), $Pd(PPh_3)_4$ (15 mg, 5 mol%) and 2 mL Et_3N. The mixture was degassed with argon for 20 min. and subsequently heated to 60 °C for 3 days. After the addition of a spatula tip of $Pd(PPh_3)_4$ and CuI, the reaction was heated again to 60 °C for 2 days. The solvent was removed under vacuum, the residue dissolved in ethyl acetate, mixed with celite and evaporated again. The crude was first purified via column chromatography (SiO_2, DCM : MeOH 100:0 → 95:5). Fractions containing the product were combined and purified via RP-MPLC (RP18, ACN : H2O 50:50 → 100:0), yielding fractions each enriched with the *E*- or *Z*-isomer. The product was obtained as a bright yellow viscous oil (*E*: 17 mg, 0.018 mmol, 8 %; *Z*: 83 mg, 0.086 mmol, 37%). Molar mass: 966.10 g/mol, Molecular formula: $C_{49}H_{67}N_5O_{15}$, *Z*: ^{1}H-NMR (400 MHz, Acetone-d_6) δ = 11.02 (s, 1H), 10.28 (s, 1H), 9.20 (s, 1H), 8.53 (s, 1H), 7.99 (s, 1H), 7.72 (d, *J* = 8.7 Hz, 2H), 7.62 (d, *J* = 8.6 Hz, 2H), 7.24 (d, *J* = 4.1 Hz, 1H), 6.95 (d, *J* = 4.1 Hz, 1H), 6.89 (s, 2H), 4.30 – 4.10 (m, 6H), 3.91 – 3.82 (m, 4H), 3.82 – 3.74 (m, 2H), 3.74 – 3.65 (m, 6H), 3.65 – 3.55 (m, 12H), 3.48 (m, 6H), 3.29 (s, 9H), 1.53 (s, 9H) ppm. ^{13}C-NMR (151 MHz, Acetone-d_6) δ = 159.84, 153.76, 140.71, 135.06, 133.24, 132.98, 132.35, 131.23, 129.92, 126.05, 124.22, 119.18, 118.38, 116.01, 115.44, 111.86, 105.48, 92.25, 88.49, 83.42, 73.30, 72.70, 71.47, 71.36, 71.32, 71.31, 71.24, 71.14, 71.11, 70.39, 69.89, 58.82, 28.15 ppm. IR (ATR): 3735, 3677, 3371, 3195, 2870, 2210, 1721, 1620, 1569, 1551, 1526, 1510, 1456,

1420, 1393, 1341, 1262, 1237, 1214, 1091, 942, 836, 778, 753, 671, 624, 606, 569, 530, 457, 441, 419 cm^{-1}. HR-MS (ESI-pos.), m/z: 966.4712 (calcd. 966.4706 for $[C_{49}H_{68}N_5O_{15}]^+$).

Boc-β-Amphiphile (4β)

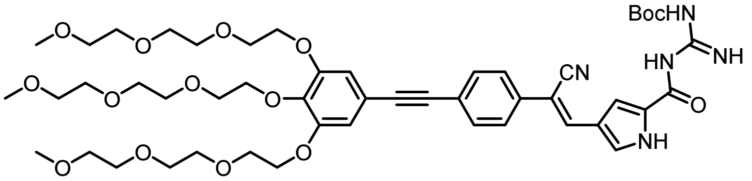

5[3] (255 mg, 0.37 mmol, 1.0 eq.) was added to 2 mL THF via syringe before addition of **3β** (149 mg, 0.37 mmol, 1.0 eq.), CuI (1.5 mg, 2 mol%), $Pd(PPh_3)_4$ (26 mg, 4 mol%) and 5 mL Et_3N. The mixture was degassed with argon for 15 min. and subsequently heated to 60 °C for 4 days. After the addition of a spatula tip of $Pd(PPh_3)_4$ and CuI, the reaction was heated again to 60 °C for 8 days. The solvent was removed under vacuum, the residue dissolved in ethyl acetate, mixed with celite and evaporated again. The crude was first purified via column chromatography (SiO_2, DCM : MeOH 100:0 → 95:5). Fractions containing the product were combined and purified via RP-MPLC (RP18, ACN : H_2O 50:50 → 100:0), yielding fractions enriched with the *Z*-isomer (97%). The product was obtained as a bright yellow viscous oil (120 mg, 0.12 mmol, 34%). Molar mass: 966.10 g/mol, Molecular formula: $C_{49}H_{67}N_5O_{15}$, ^{1}H-NMR (400 MHz, Acetone-d_6) δ = 11.09 (s, 1H), 10.41 (s, 1H), 9.20 (s, 1H), 8.49 (s, 1H), 7.90 (s, 1H), 7.72 (d, *J* = 8.6 Hz, 2H), 7.66 (d, *J* = 2.4 Hz, 1H), 7.60 (m, 3H), 6.89 (s, 2H), 4.34 – 4.13 (m, 6H), 3.92 – 3.82 (m, 4H), 3.82 – 3.75 (m, 2H), 3.75 – 3.64 (m, 6H), 3.65 – 3.54 (m, 12H), 3.48 (m, 6H), 3.29 (m, 9H), 1.52 (s, 9H) ppm. ^{13}C-NMR (151 MHz, Acetone-d_6) δ = 159.76, 153.75, 140.63, 138.14, 135.76, 133.02, 132.83, 130.49, 129.97, 128.31, 125.95, 123.60, 118.48, 112.77, 111.83, 91.81, 88.57, 83.36, 73.29, 72.69, 71.47, 71.36, 71.32, 71.29, 71.24, 71.14, 71.11, 70.39, 69.88, 58.82, 28.15 ppm. IR (ATR): 3377, 3258, 2871, 2206, 1725, 1629, 1606, 1560, 1532, 1511, 1497, 1450, 1417, 1391, 1366, 1348, 1286, 1236, 1093, 941, 840, 781, 754, 674, 642, 616, 533, 442, 417, 405 cm^{-1}. HR-MS (ESI-pos.), m/z: 966.4746 (calcd. 966.4706 for $[C_{49}H_{68}N_5O_{15}]^+$).

α-Amphiphile (Aα)

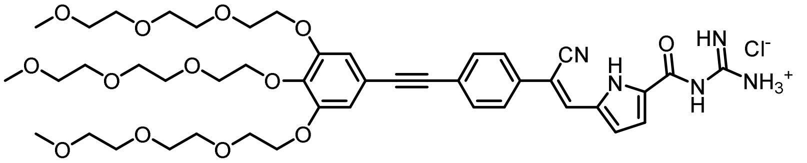

Z-**4α** (42 mg, 0.04 mmol) was dissolved in 3 mL of THF, and 1.5 mL of 6 M HCl was added. The mixture was then stirred overnight. After cooling, the solvent was removed under vacuum, and acetone was added and evaporated to remove acid residues. The obtained product was mixed with 20 mL of 1 M HCl and evaporated. After repeating the process once, acetone was added and evaporated to remove water, yielding *Z*-**Aα** as a yellow, highly viscous oil (39 mg, 0.04 mmol, quant.). Molar mass: 902.44 g/mol, Molecular formula: $C_{44}H_{60}N_5O_{15}Cl$, ^{1}H-NMR (400 MHz, Acetone-d_6) δ = 13.51 (s, 1H), 12.04 (s, 1H), 9.20 (s, 2H), 8.03 (s, 2H), 7.93 (m, 2H), 7.75 (d, *J* = 8.3 Hz, 2H), 7.65 (d, *J* = 8.4 Hz, 3H), 7.37 (d, *J* = 4.3 Hz, 1H), 6.90 (s, 2H), 4.28 – 4.16 (m, 6H), 3.90 – 3.83 (m, 4H), 3.79 (m, 2H), 3.69 (m, 6H), 3.65 – 3.52 (m, 12H), 3.51 – 3.45 (m, 6H), 3.30 (s, 3H), 3.29 (s, 6H) ppm. ^{13}C-NMR* (151 MHz, Acetone-d_6) δ = 153.70, 140.58, 135.19, 133.30, 132.94, 132.48, 130.39 (br), 129.95, 125.93, 123.95, 119.37, 118.46, 115.67, 115.12 (br), 111.83, 104.49 (br), 92.11, 88.58, 73.29, 72.67, 71.44, 71.33, 71.29, 71.26, 71.20, 71.11, 71.08, 70.37, 69.86, 58.82 ppm. IR (ATR): 3567, 3353, 3144, 3094, 2986, 2870, 2366, 2339, 2255, 2210, 2172, 1684, 1574, 1550, 1512, 1498, 1462, 1418, 1347, 1326, 1253, 1199, 1099, 1018, 944, 833, 806, 750, 670, 624, 603, 578, 568, 556, 526, 491 cm^{-1}. HR-MS (ESI-pos.), m/z: 866.4174 (calcd. 866.4182 for $[C_{44}H_{60}N_5O_{13}]^+$).

*The HCl salt aggregates in organic solvents. ^{13}C-NMR sample was stoichiometrically deprotonated with NaOH and lyophilized before measurement to enhance resolution.

β-Amphiphile (Aβ)

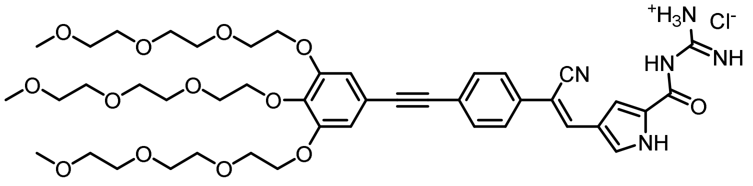

Z-**4β** (43 mg, 0.04 mmol) was dissolved in 3 mL of THF, and 1.5 mL of 6 M HCl was added. The mixture was then stirred at overnight. After cooling, the solvent was removed under vacuum, and acetone was added and evaporated to remove acid residues. The crude product was purified via RP-MPLC (RP18, ACN : H_2O (TFA 0.1%) 50:50 → 100:0) with the UV detector turned off. The obtained product was mixed with 20 mL of 1 M HCl and evaporated. After repeating the process once, acetone was added and evaporated to remove water, yielding a yellow, highly viscous oil (*Z*:*E* = 4:1, 40 mg, 0.04 mmol, quant.). Molar mass: 902.44 g/mol, Molecular formula: $C_{44}H_{60}N_5O_{15}Cl$, ^{1}H- NMR (400 MHz, Acetone-d_6) δ = 12.92 (s, 1H), 12.36 (s, 1H), 8.82 (s, 2H), 8.07 – 7.96 (m, 4H), 7.95 (s, 1H), 7.76 (d, J = 8.0 Hz, 2H), 7.61 (d, J = 7.9 Hz, 2H), 6.90 (s, 2H), 4.28 – 4.14 (m, 6H), 3.87 (m, 4H), 3.79 (m, 2H), 3.70 (m, 6H), 3.65 – 3.56 (m, 12H), 3.54 – 3.43 (m, 6H), 3.30 (s, 3H), 3.29 (s, 6H) ppm. ^{13}C-NMR* (151 MHz, Acetone-d_6) δ = 153.54, 140.02, 137.82, 135.59, 133.13, 132.84, 129.96, 128.39, 126.03, 123.63, 121.74, 119.41 (br), 118.79, 111.75, 111.68, 105.35 (br), 91.74, 88.73, 73.29, 72.60, 72.54, 71.29, 71.22, 71.07, 71.04, 71.02, 70.97, 70.23, 69.68, 58.86 ppm. IR (ATR): 3308, 3167, 2871, 2364, 2248, 2209, 2182, 2170, 1691, 1637, 1607, 1573, 1511, 1498, 1447, 1420, 1379, 1348, 1282, 1249, 1220, 1087, 1027, 944, 837, 755, 722, 670, 636, 617, 574, 518, 458, 431, 403 cm^{-1}. HR-MS (ESI-pos.), m/z: 866.4185 (calcd. 866.4182 for $[C_{44}H_{60}N_5O_{13}]^+$).

*The HCl salt aggregates in organic solvents. ^{13}C-NMR sample was stoichiometrically deprotonated with NaOH and lyophilized before measurement to enhance resolution.

1,3,6,8-Tetracyanopyrene (7)[4]

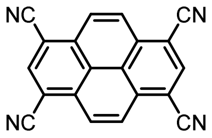

1,3,6,8-Tetrabromopyrene (1.00 g, 1.93 mmol, 1.0 eq.) and CuCN (0.86 g, 9.66 mmol, 5.0 eq.) were suspended in 16 mL *N*-Methyl-2-pyrrolidone and stirred at 180 °C for 90 h. After cooling to room temperature, 40 mL of 12 % NH_3 (aq.) was added slowly, and the mixture was stirred for 30 minutes. The suspension was filtered, and the precipitate was subsequently washed with 12% NH_3 (aq.), water, and diethyl ether. The precipitate was dried, giving 0.62 g (>100%) of an insoluble brown solid, which was used without further characterization.

Pyrene-1,3,6,8-tetracarboxylic acid (8)

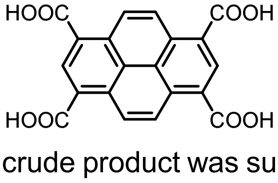

The synthesis was adapted from protocols of Wu[5] and Huang.[6] **7** (0.62 g) was refluxed with 20 mL 10 M NaOH and 30 mL ethanol for 24 h. After cooling to r.t., the pH was adjusted to pH = 1 by the addition of 37% HCl. Filtration yielded 0.90 g of a dark brown solid. 0.62 g of the crude product was suspended in 10 mL of *n*-butanol and treated with six drops of concentrated H_2SO_4. The mixture was then stirred at 120 °C for 36 h before being cooled and filtered. The filtrate was evaporated and the residue purified using column chromatography (SiO_2, diethyl ether : toluene : hexane 1:3:16). 140 mg of the tetrabutyl-pyrene-1,3,6,8-tetracarboxylate (0.23 mmol) was obtained and dissolved in 7.5 mL THF and 2.5 mL water. KOH (0.39 g, 6.97 mmol, 30 eq.) was added, and the mixture was stirred at 100 °C for 22 h. After cooling, THF was evaporated, and the aqueous residue was treated with 6.5 M HCl to yield a yellow solid, which

was filtered and washed thoroughly with hot water. After drying, 60 mg of a yellow solid could be obtained (0.16 mmol, 68% referring to the tetrabutyl ester). Molar mass: 378.29 g/mol, Molecular formula: $C_{20}H_{10}O_8$, ^{1}H-NMR (400 MHz, DMSO-d_6) δ = 13.82 (s, 4H), 9.41 (s, 4H), 9.15 (s, 2H) ppm. ^{13}C-NMR (101 MHz, DMSO-d_6) δ = 168.25, 131.40, 130.76, 127.70, 126.29, 124.18 ppm. IR (ATR): 3060, 2919, 2850, 2610, 2510, 2359, 1690, 1557, 1461, 1408, 1286, 1255, 1237, 1146, 1122, 1035, 901, 846, 801, 770, 705, 664, 649, 497, 487, 478, 462, 452, 422, 411 cm^{-1}. HR-MS (ESI-neg.), m/z: 377.0300 (calcd. 377.3030 for $[C_{20}H_9O_8]^-$).

Sodium pyrene-1,3,6,8-tetracarboxylate (TCP) stock solution

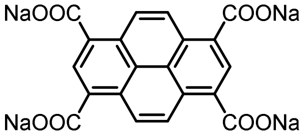

Pyrene-1,3,6,8-tetracarboxylic acid (**8**) (5 mg, 13.2 µmol) was mixed with 529 µL (4.0 eq.) of a freshly prepared 0.1 M NaOH solution in MQ-water, 1 mL MQ-water and stirred for 5 minutes. After that, the flask was freeze-dried. The dried **TCP** was dissolved in exactly 1.322 mL MQ-water to give a 10 mM stock solution.

3. NMR Spectra

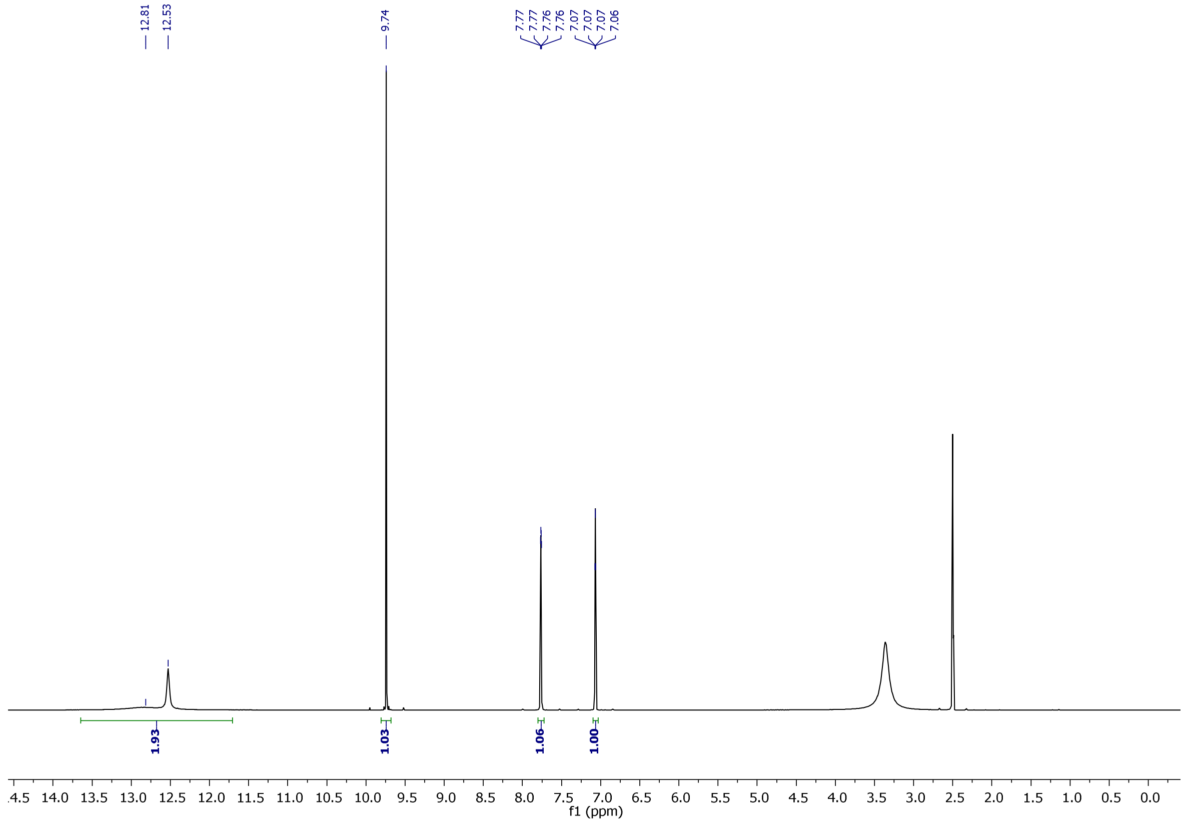

Fig. S1: ^{1}H-NMR of **2bβ** (DMSO-d_6, 400 MHz).

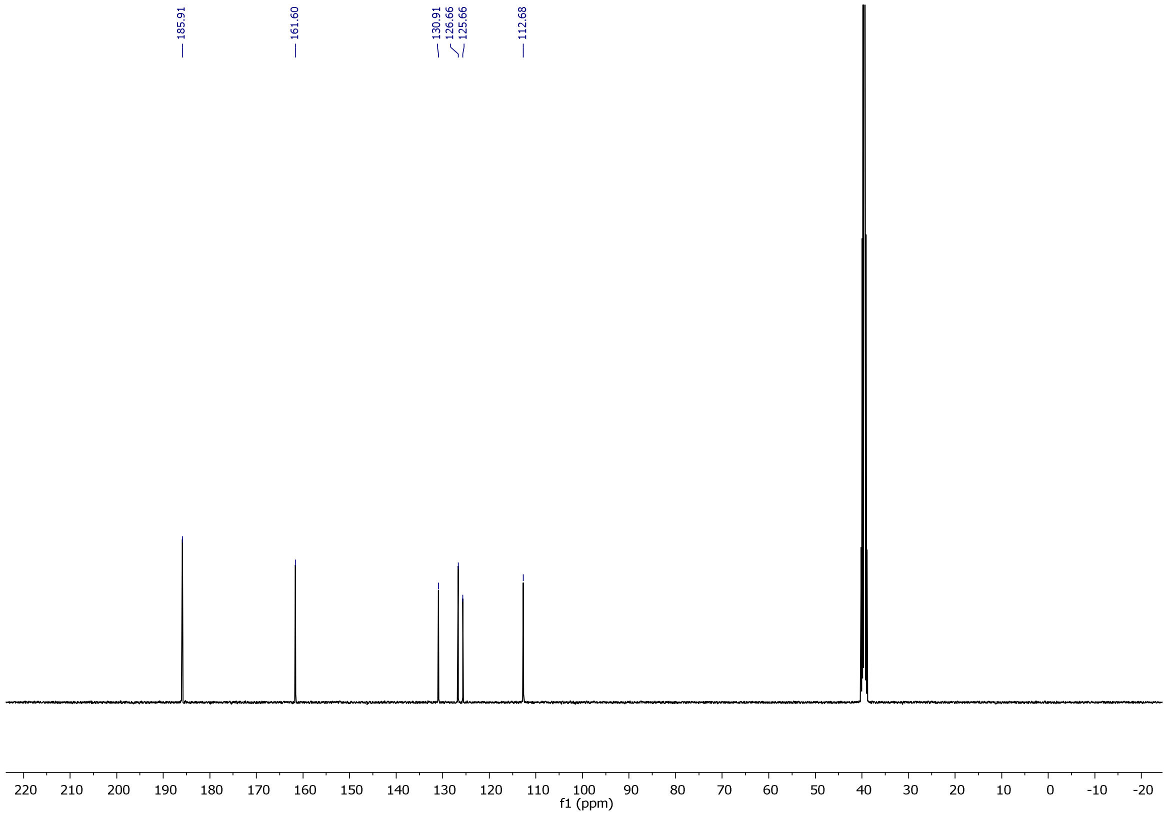

Fig. S2: ^{13}C-NMR of **2bβ** (DMSO-d_6, 101 MHz).

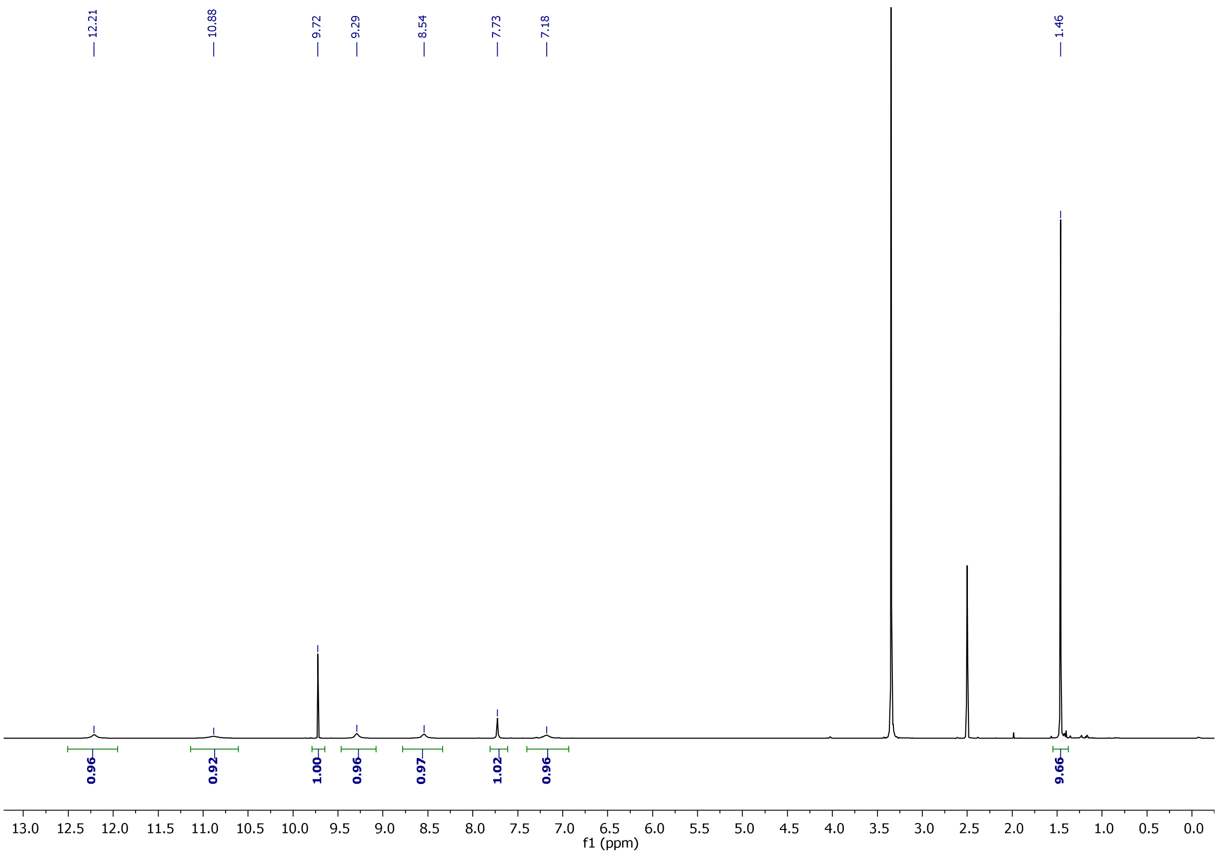

Fig. S3: ^{1}H-NMR of **2β** (DMSO-d_6, 400 MHz).

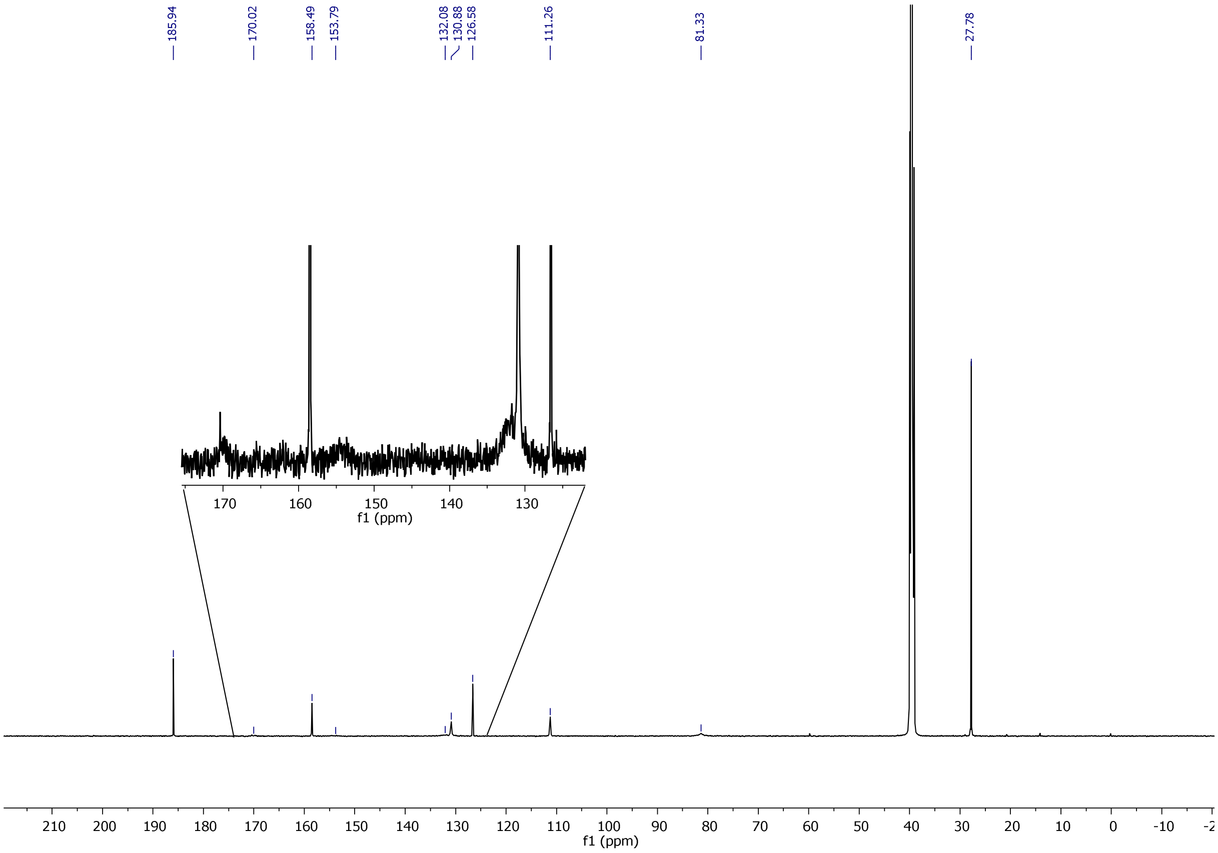

Fig. S4: ^{13}C-NMR of **2β** (DMSO-d_6, 101 MHz).

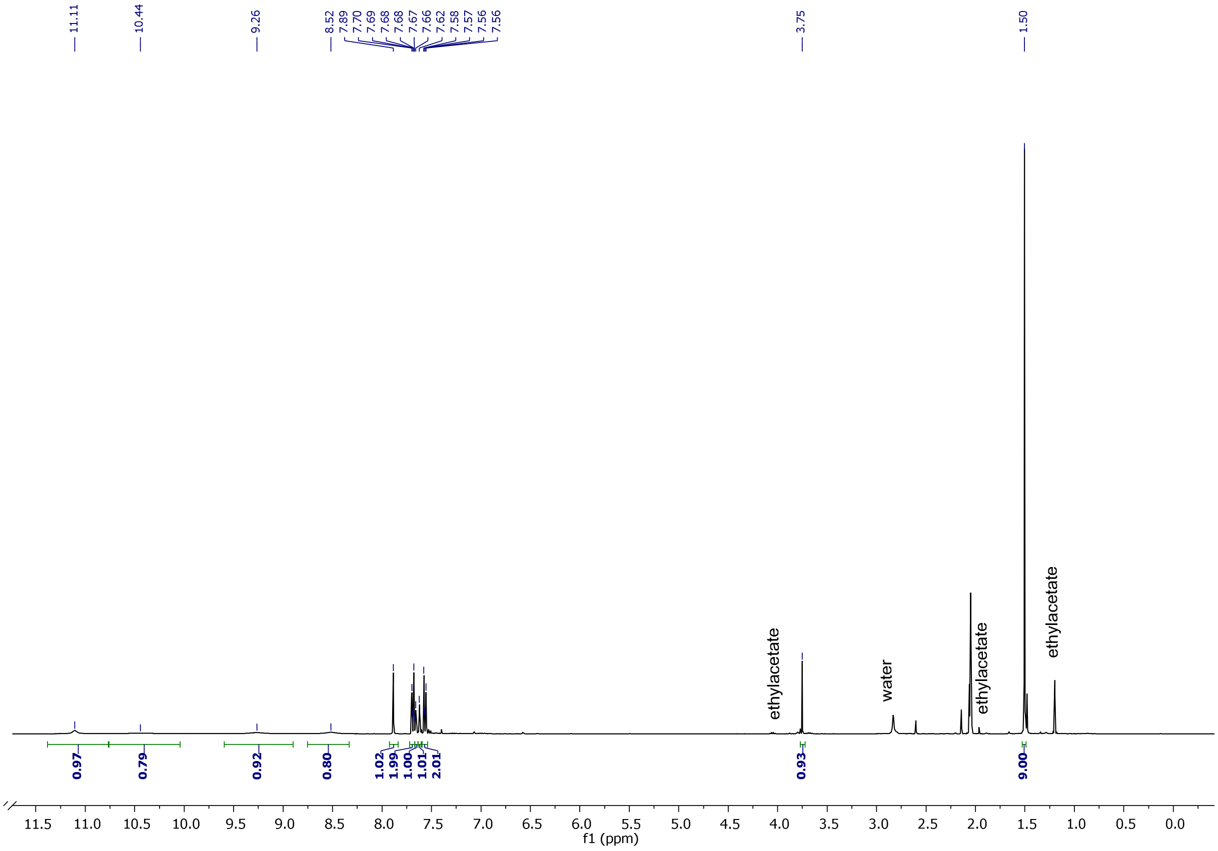

Fig. S5: ^{1}H-NMR of **3β** (Acetone-d_6, 400 MHz).

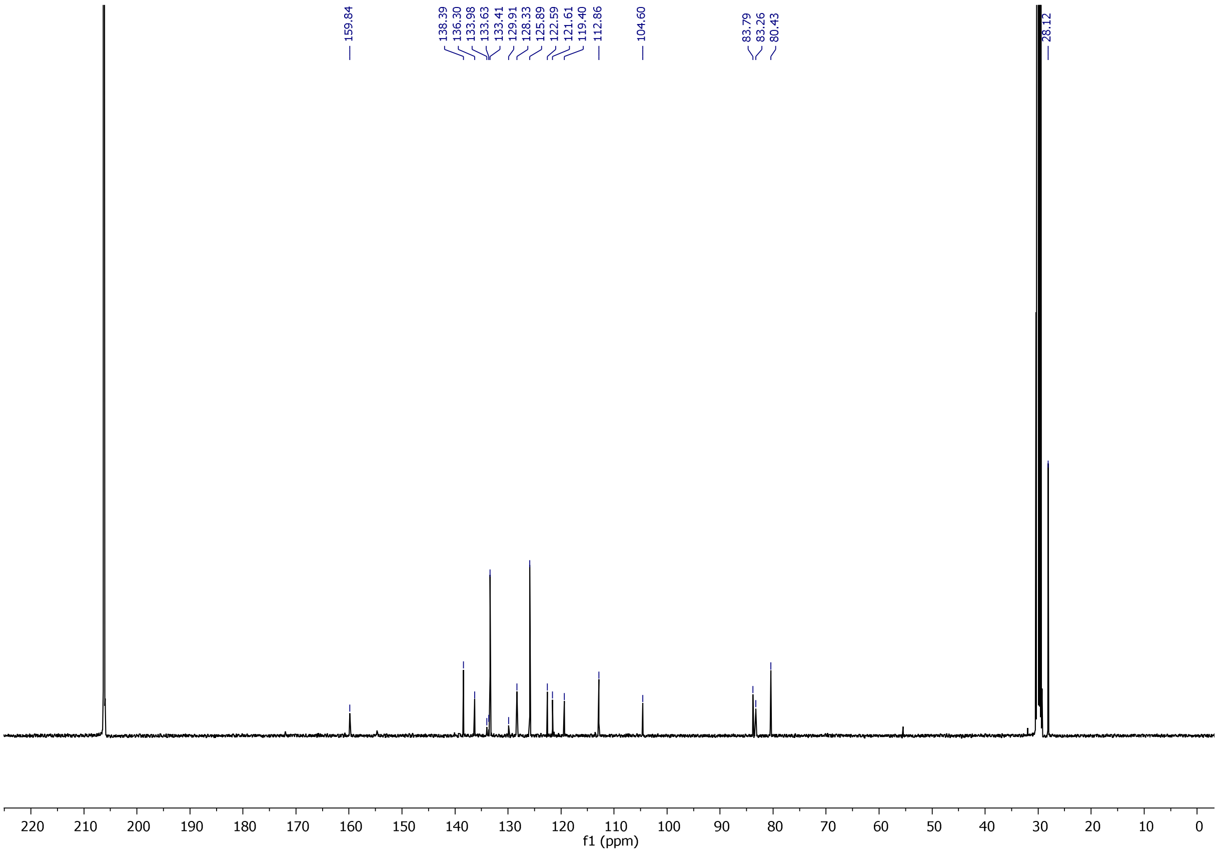

Fig. S6: ^{13}C-NMR of **3β** (Acetone-d_6, 101 MHz).

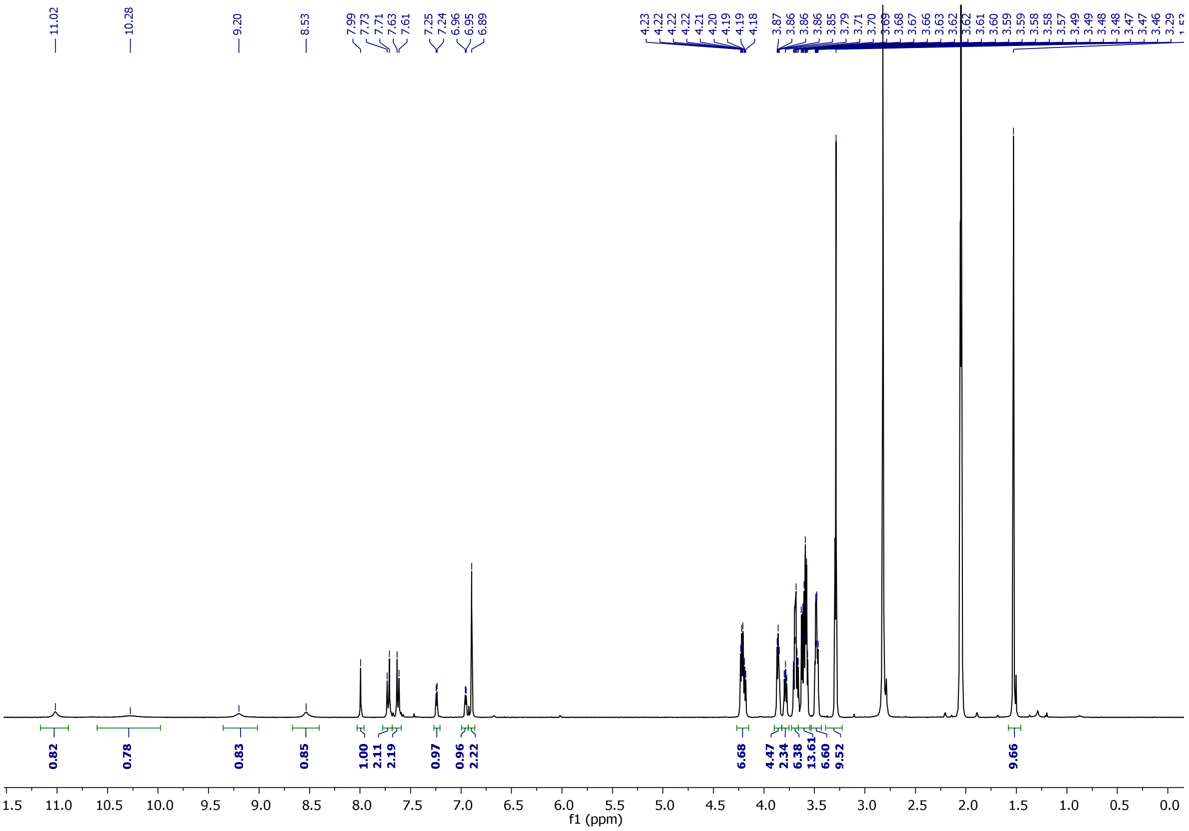

Fig. S7: ^{1}H-NMR of **4α** (Acetone-d_6, 600 MHz).

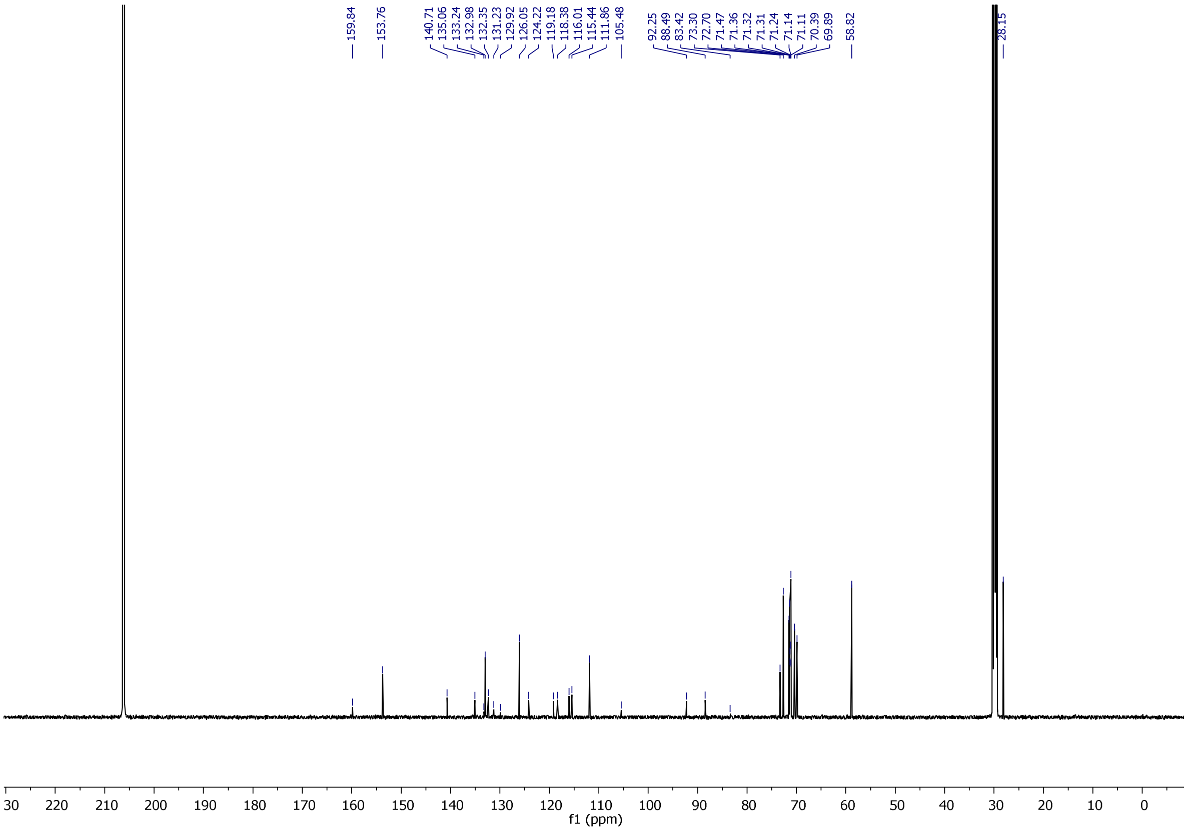

Fig. S8: ^{13}C-NMR of **4α** (Acetone-d_6, 151 MHz).

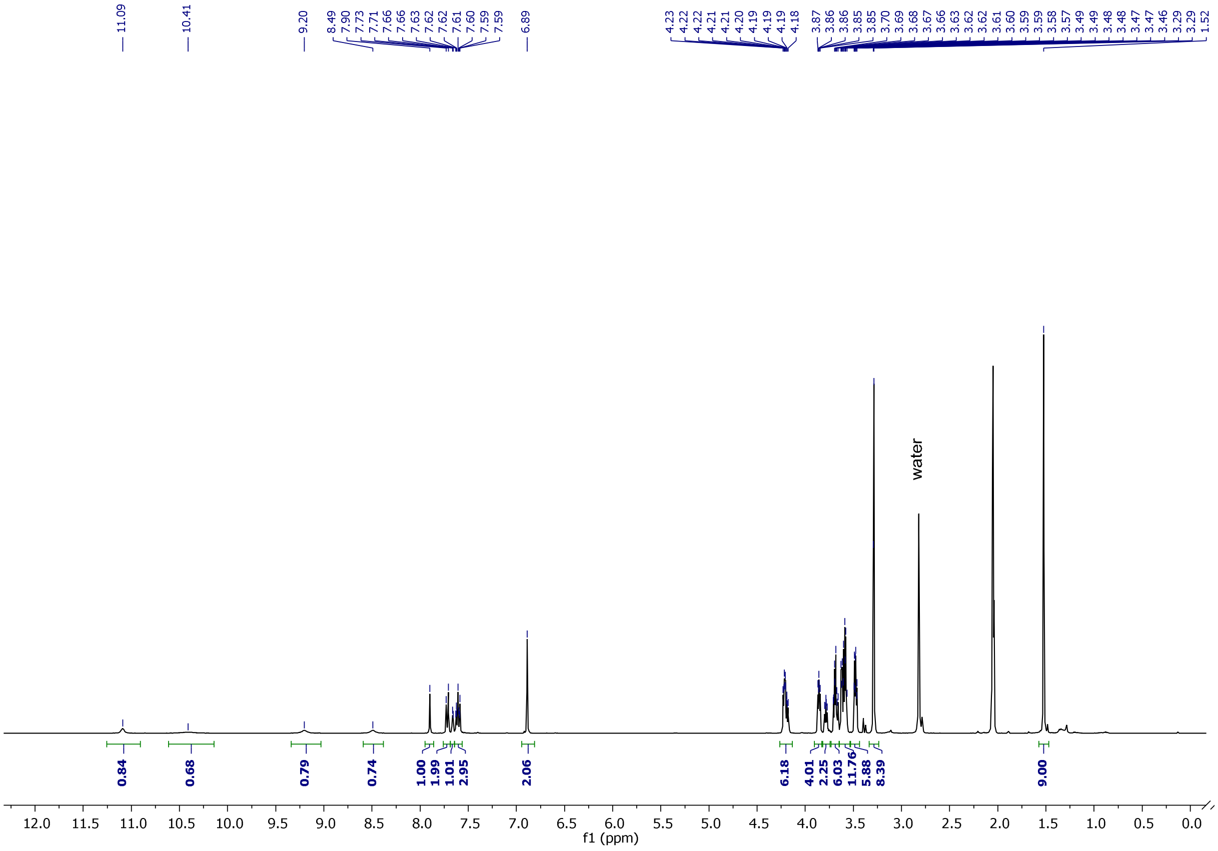

Fig. S9: ^{1}H-NMR of **4β** (Acetone-d_6, 600 MHz).

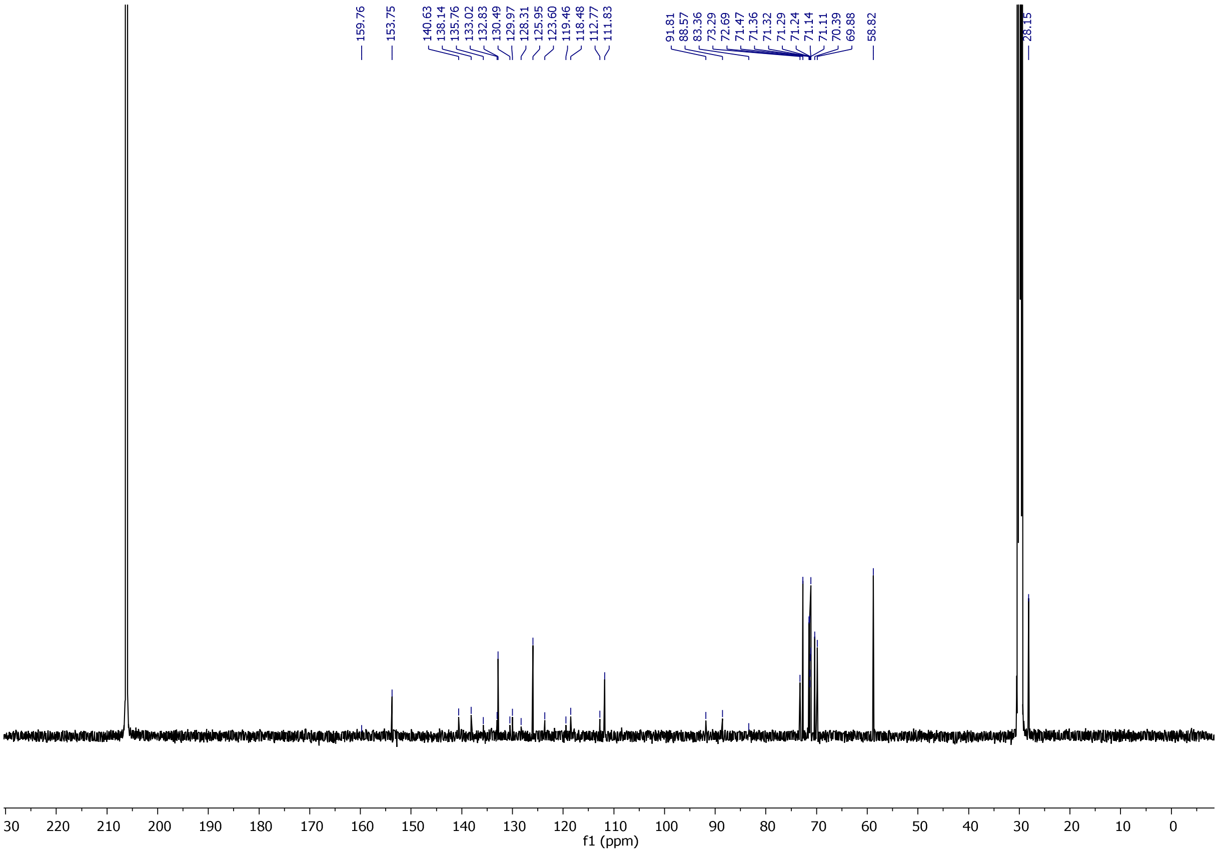

Fig. 10: ^{13}C-NMR of **4β** (Acetone-d_6, 151 MHz).

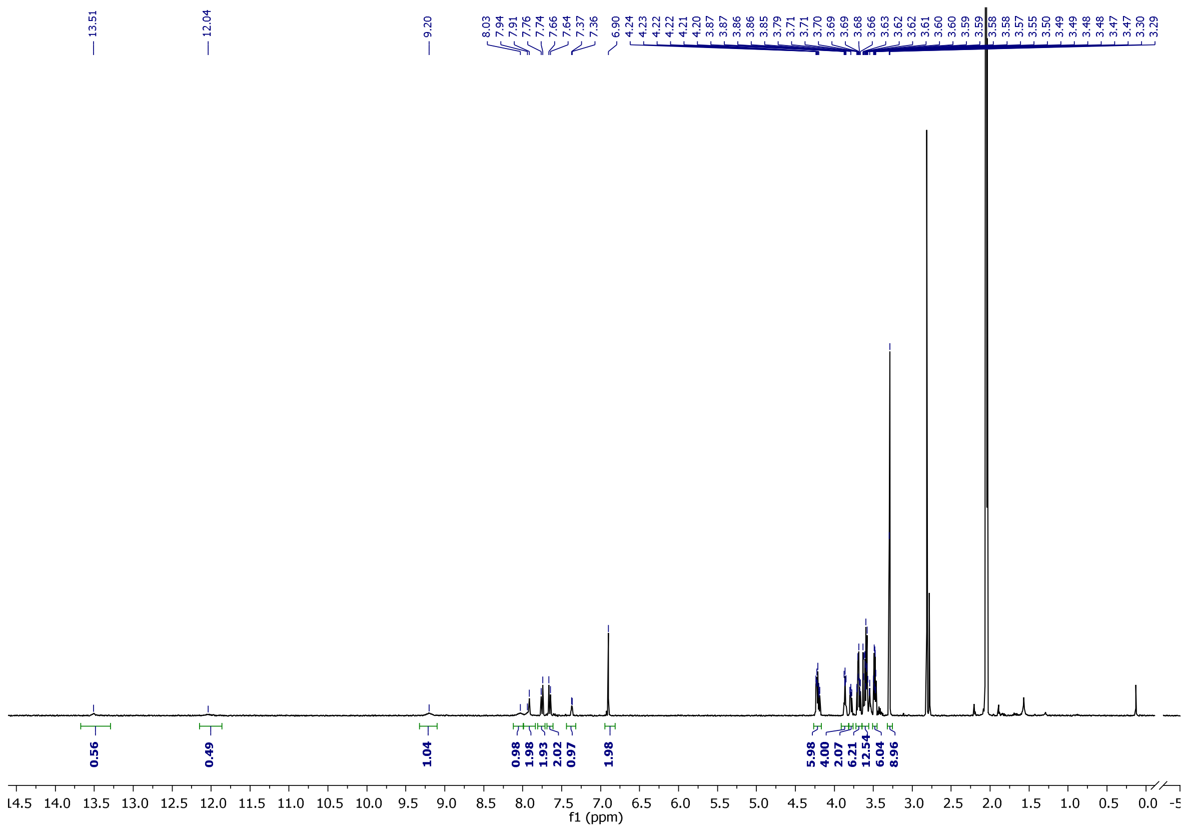

Fig. S11: ^{1}H-NMR of **Aα** (Acetone-d_6, 600 MHz) which was neutralized by adding 1.0 eq. NaOD and freeze-dried prior to measurement.

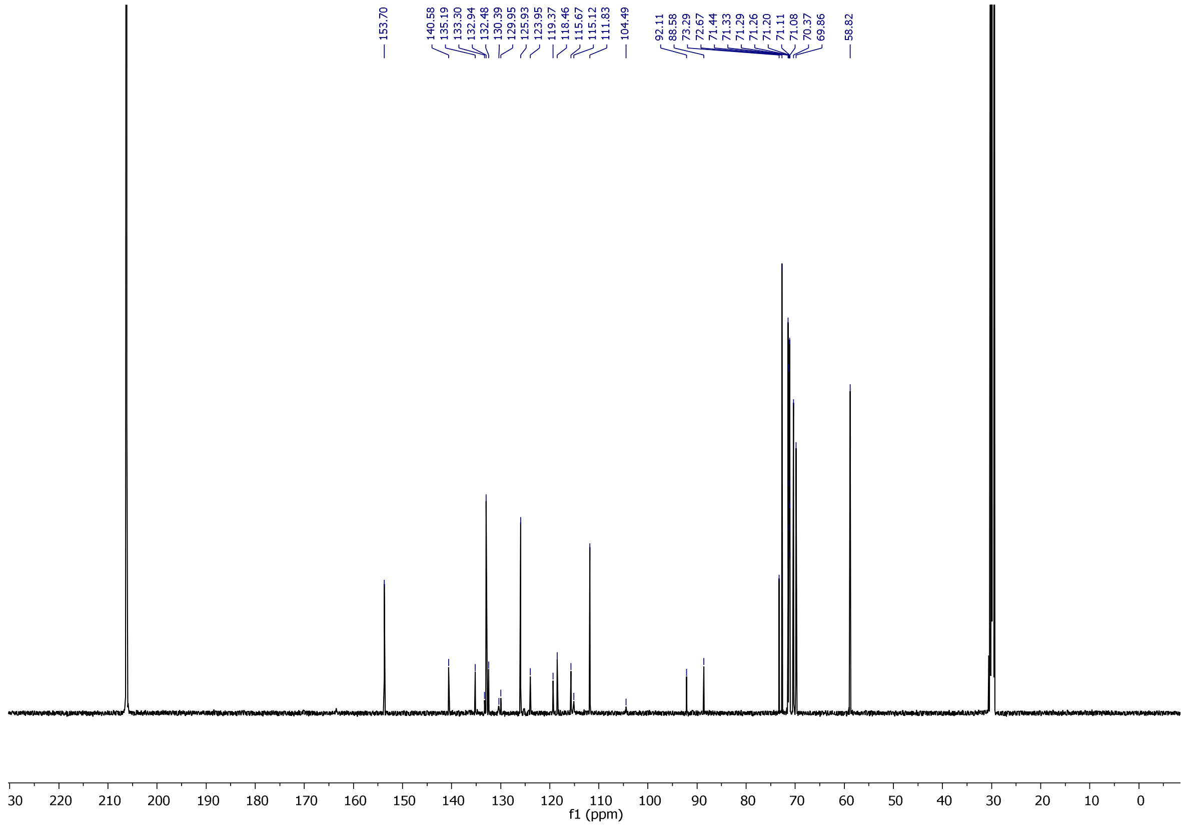

Fig. S12: ^{13}C-NMR of **Aα** (Acetone-d_6, 151 MHz) which was neutralized by adding 1.0 eq. NaOD and freeze-dried prior to measurement.

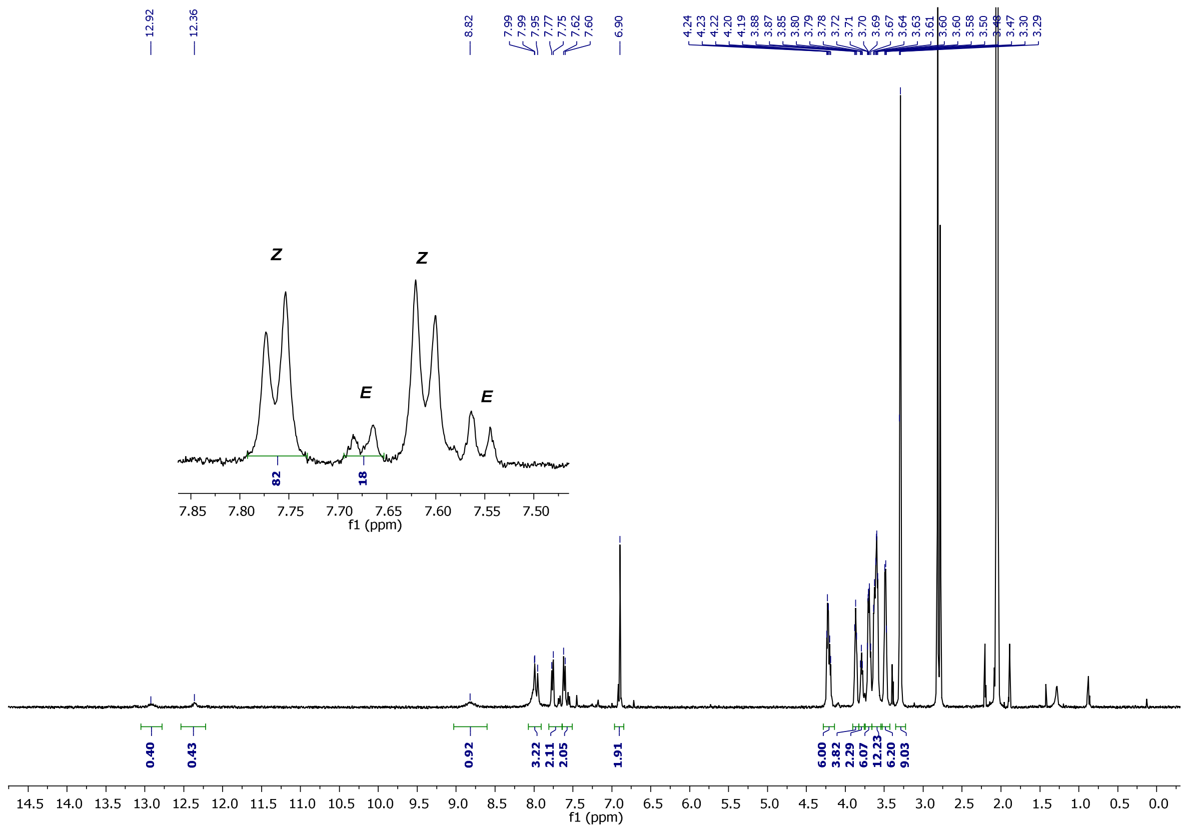

Fig. S13: ^{1}H-NMR of **Aβ** (Acetone-d_6, 600 MHz) which was neutralized by adding 1.0 eq. NaOD and freeze-dried prior to measurement.

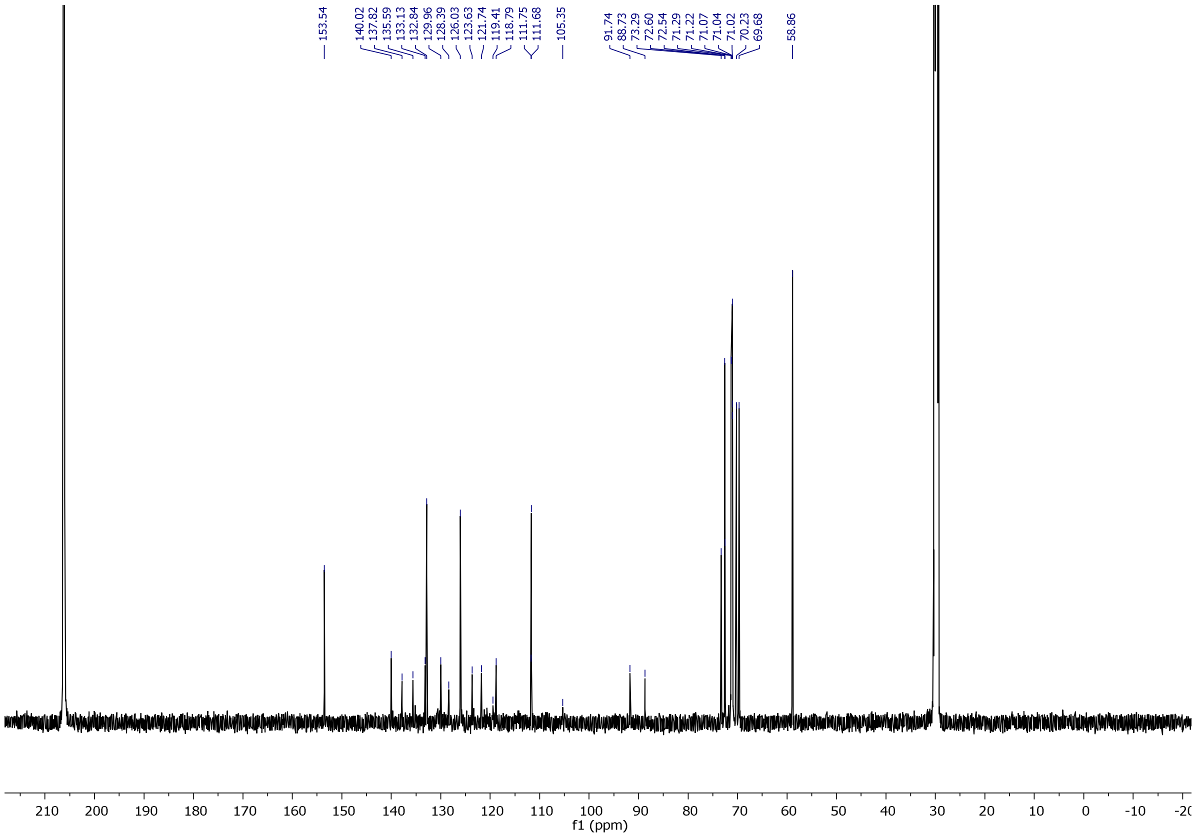

Fig. S14: ^{13}C-NMR of **Aβ** (Acetone-d_6, 151 MHz) which was neutralized by adding 1.0 eq. NaOD and freeze dried prior to measurement.

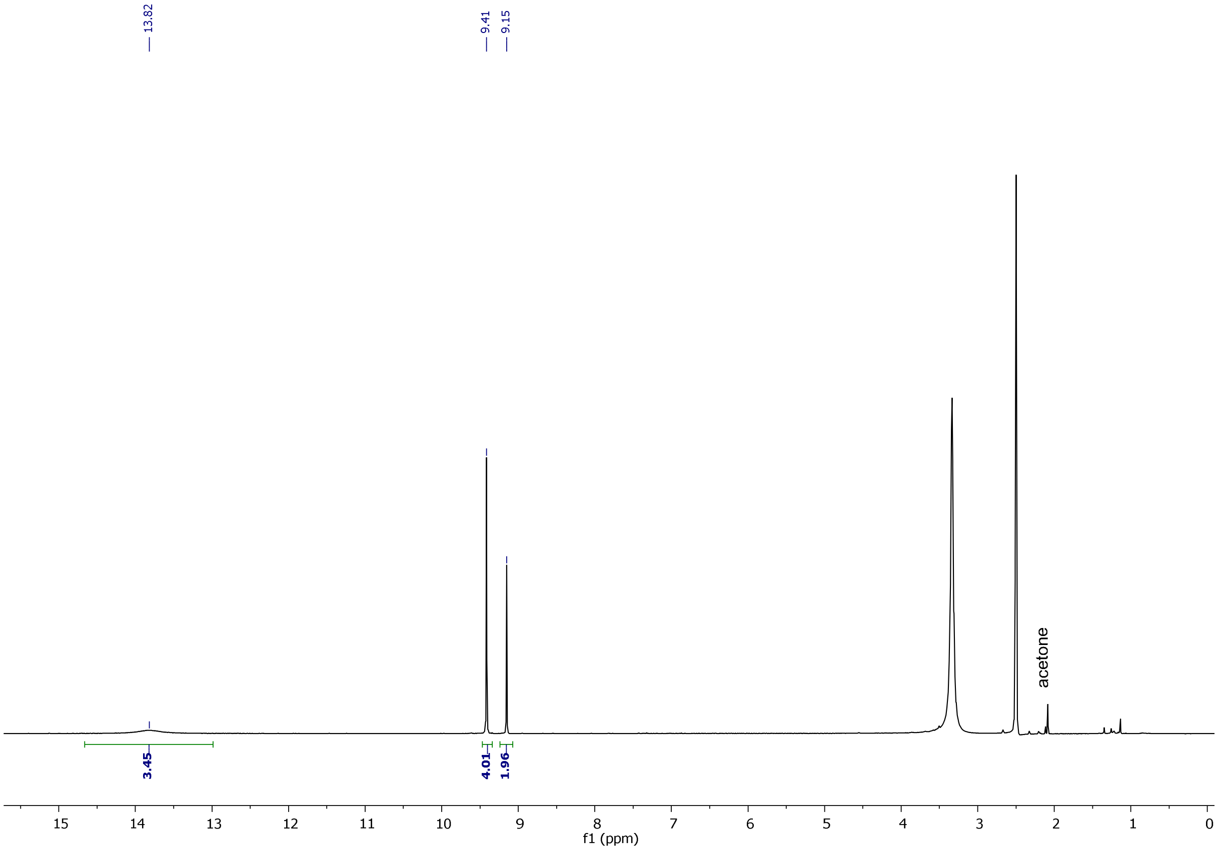

Fig. S15: ^{1}H-NMR of **7** (DMSO-d_6, 400 MHz).

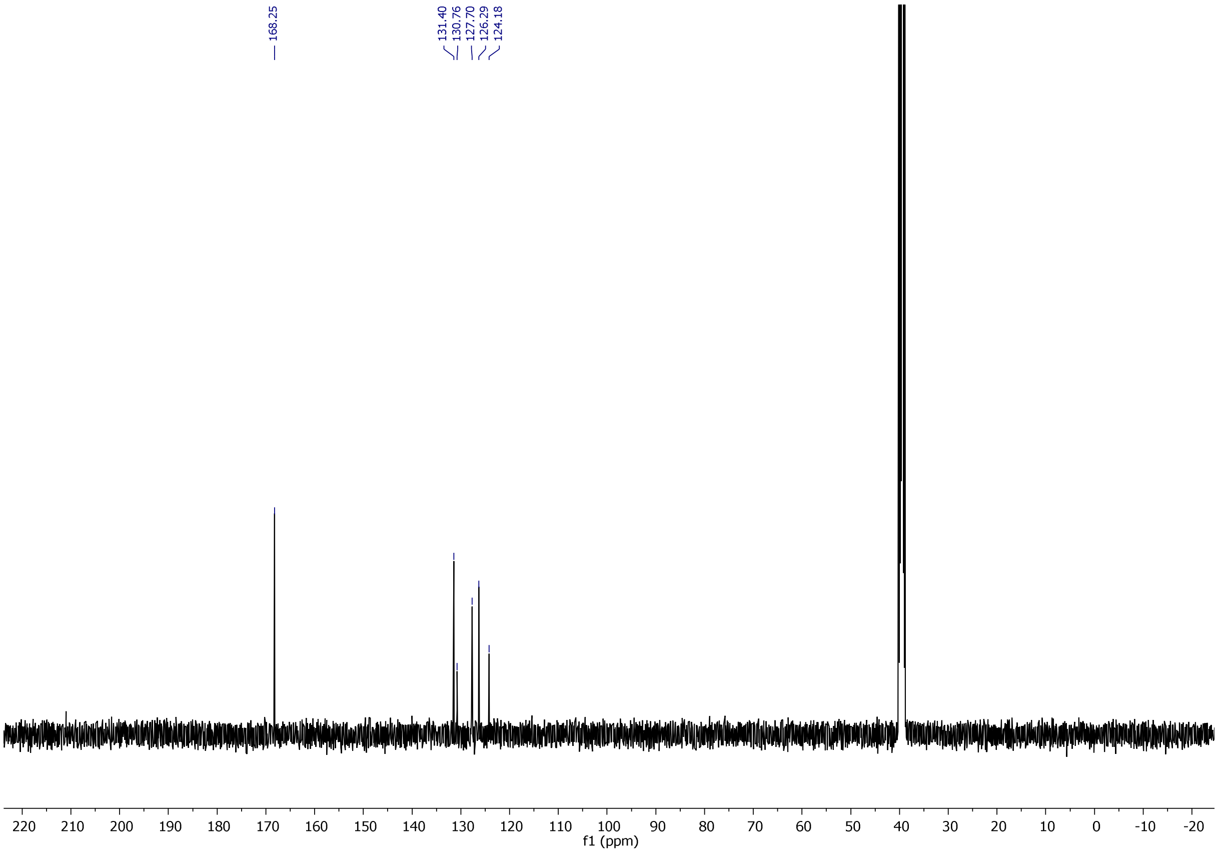

Fig. S16: ^{13}C-NMR of **7** (DMSO-d_6, 101 MHz).

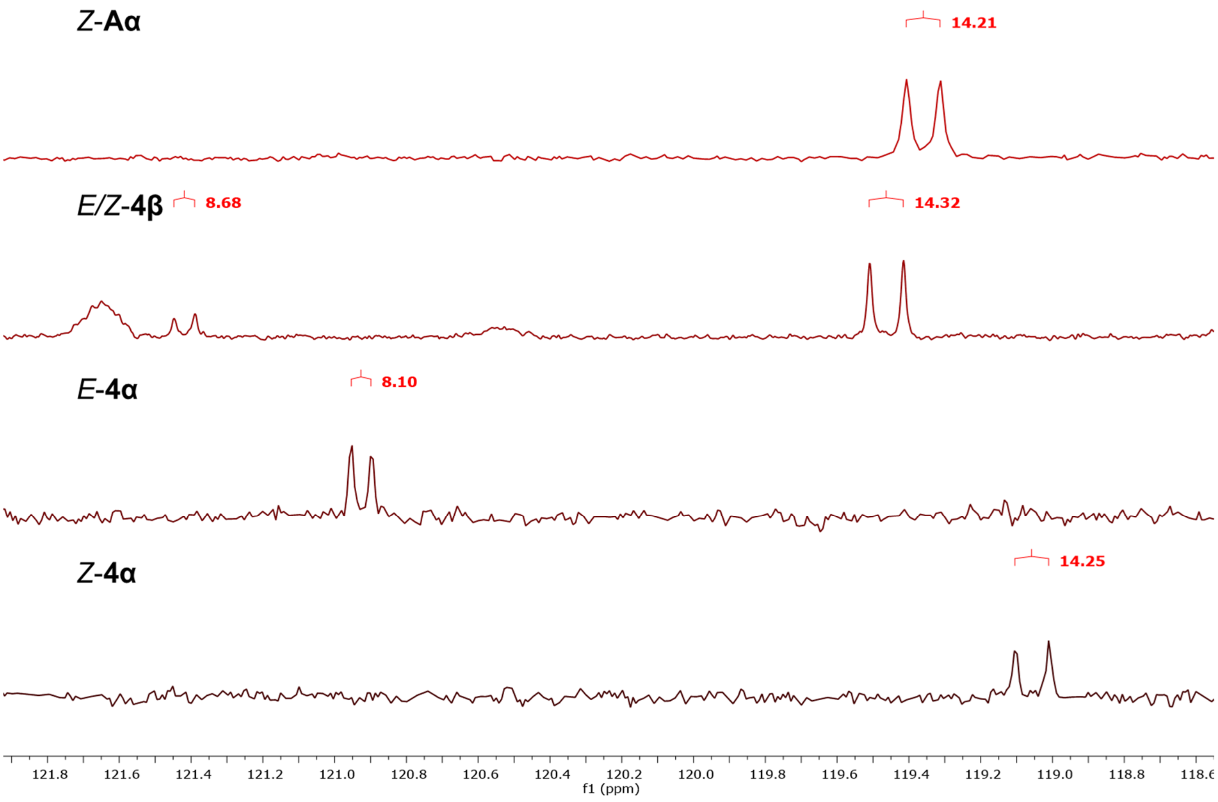

Fig. S17: ^{1}H-coupled ^{13}C-spectra (151 MHz, Acetone-d$_6$) of **4α/β** isomers and **Aα** (neutralized with 1.0 eq. NaOD and freeze-dried prior to measurement), focusing on the vicinal $^{3}J_{H,C}$-coupling of nitrile-carbon and stilbene-proton. Higher coupling constants (14.25, 14.32 and 14.21 Hz) refer to the *trans*-/*Z*-isomer while lower constants refer to the *cis*/*E*-isomer.[7]

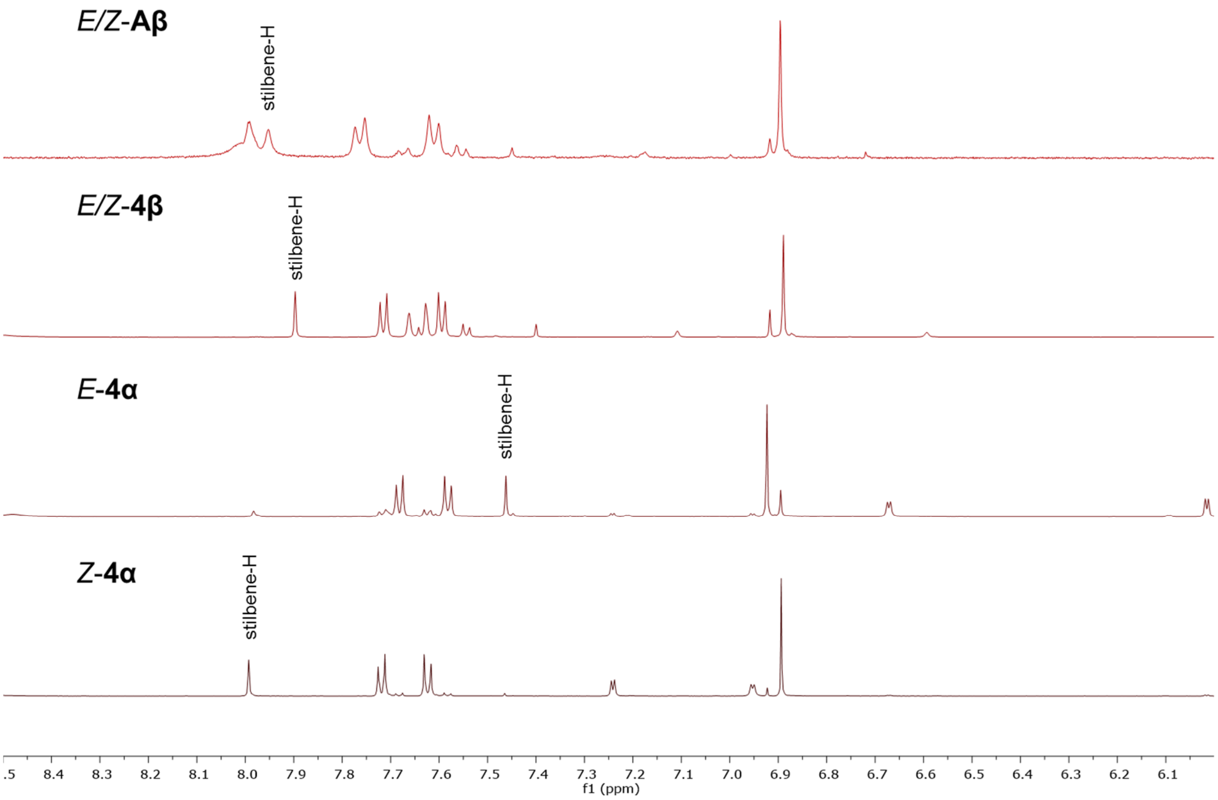

Fig. S18: ^{1}H-spectra of **4α**/**β** isomers and the **Aβ**-mixture (600 MHz, Acetone-*d*$_6$). The position of the stilbene proton and aromatic protons suggests the main isomer being in *Z*-configuration.

4. Self-assembly studies

Critical assembly-concentration (CAC)

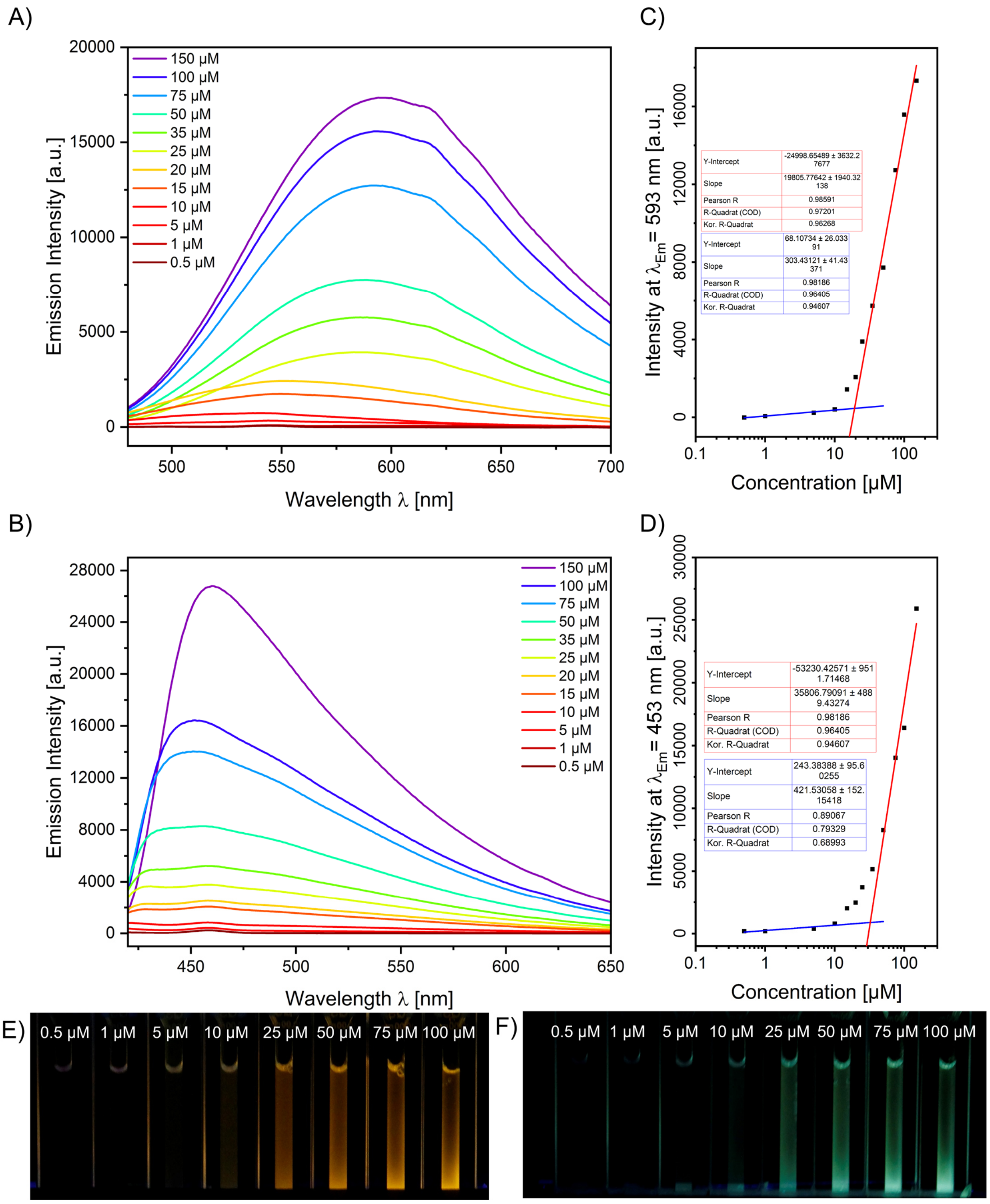

Fig. S19: Fluorescence spectra of **Aα** (A) and **Aβ** (B) at increasing concentration in water; Plotted maximum intensities of **Aα** (C) and **Aβ** (D) against concentration. Fitted straights were plotted for datapoints pre-aggregation (blue) and post-aggregation (red) to indicate CACs at the intersection; Photographs of respective samples of **Aα** (E) and **Aβ** (F) under UV light (365 nm).

To determine the CAC, the intercepts of both lines can be calculated using the following equation:

$$CAC=10^{\left(\frac{b_1-b_2}{a_1-a_2}\right)}\ \mu M$$

a_1: Slope of blue straight; b_1: Y-Intercept of blue straight; a_2: Slope of red straight; b_2: Y-Intercept of red straight.

Considering the maximum deviations from both straights, CAC values for both amphiphiles were calculated:

CAC(**Aα**) = 19 ± 2 μM CAC(**Aβ**) = 32 ± 3 μM

Dynamic light scattering (DLS) and ζ-potential measurements

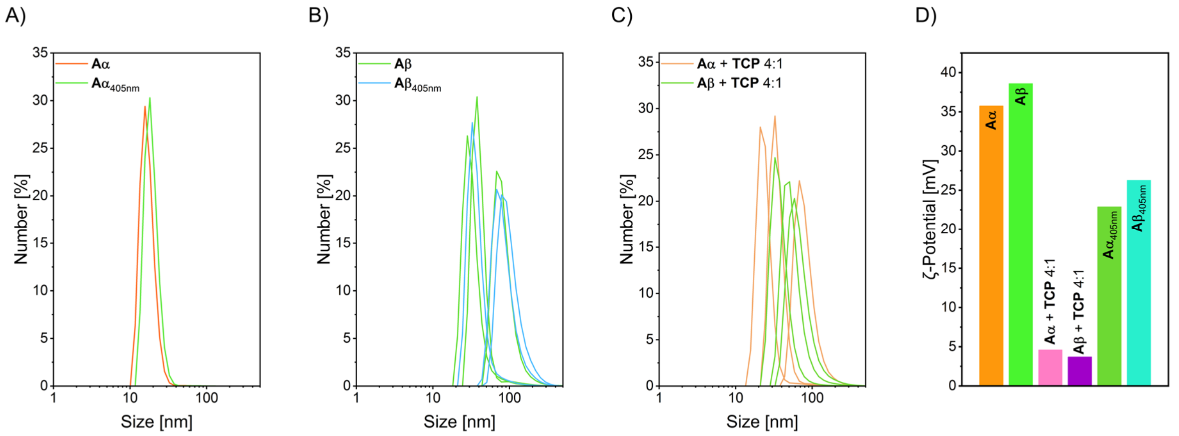

Fig. S20: DLS measurements of samples (A) 50 μM of **Aα** and **Aα405nm** (sample irradiated with 405 nm for 5 minutes before measurement), (B) **Aβ** and **Aβ405nm** (sample irradiated with 3W 405 nm LED for 5 minutes before measurement), and (C) 50 μM **Aα**/**Aβ** with 12.5 μM **TCP**. (D) ζ-potential values for amphiphiles and **TCP** complexes.

TEM

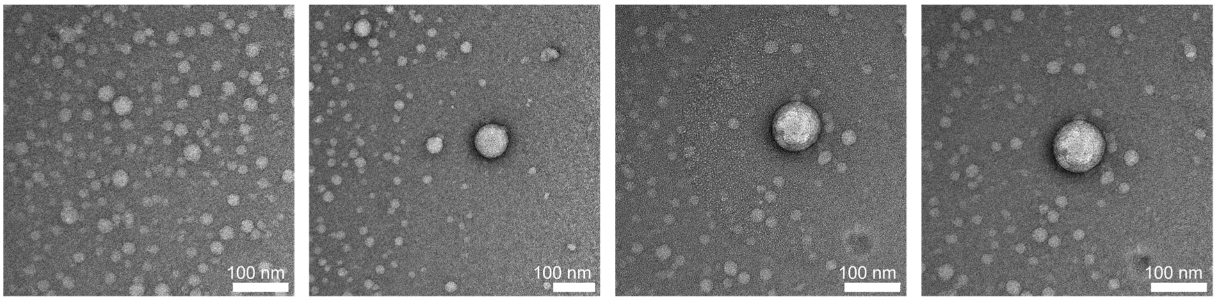

Fig. S21: TEM images of **Aα** from 100 μM solutions. Scale bar: 100 nm.

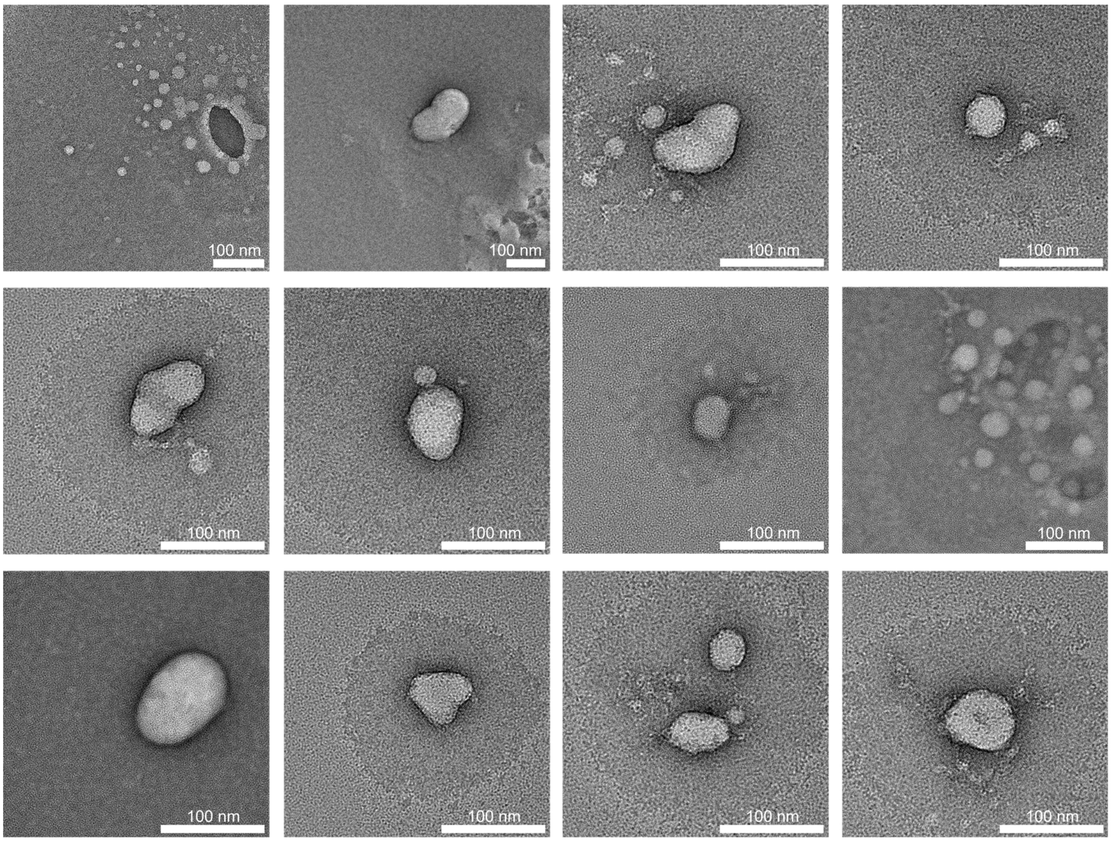

Fig. S22: TEM images of **Aβ** from 100 μM solutions. Scale bar: 100nm.

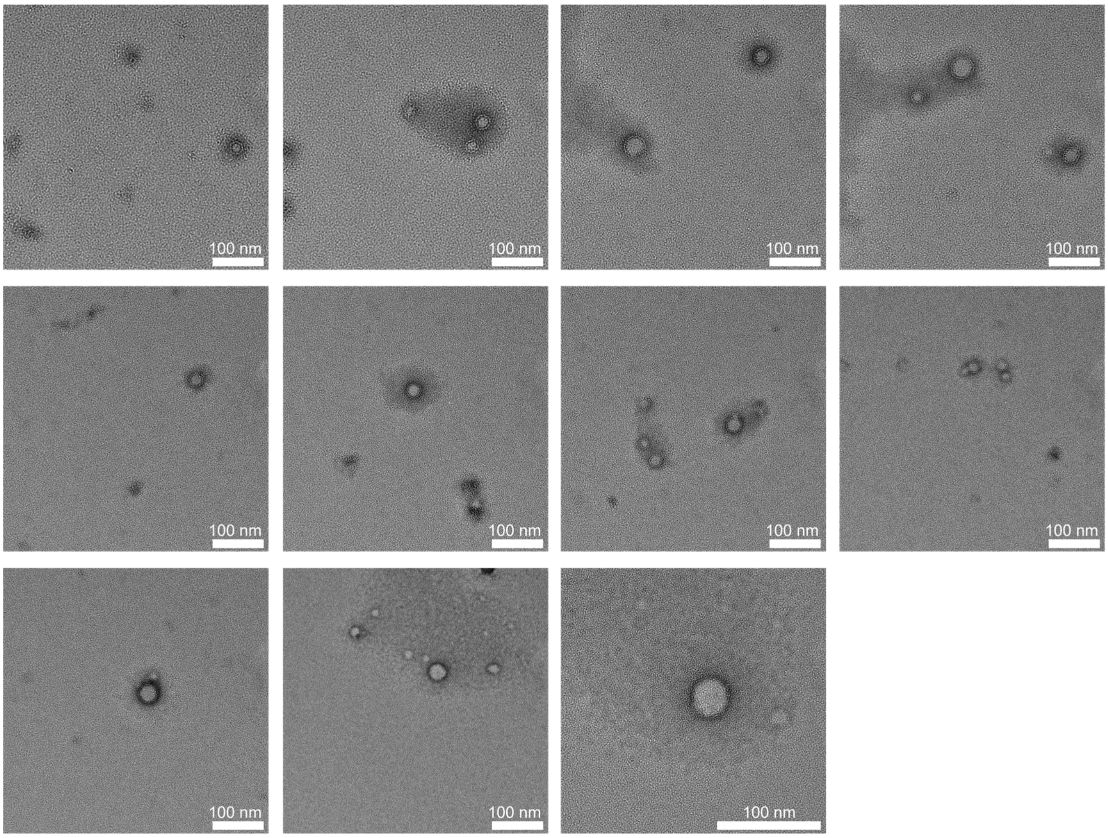

Fig. S23: TEM images of **Aα** from 100 μM solutions, which were irradiated with a 3W 405 nm LED prior to sample preparation. Scale-bar: 100 nm.

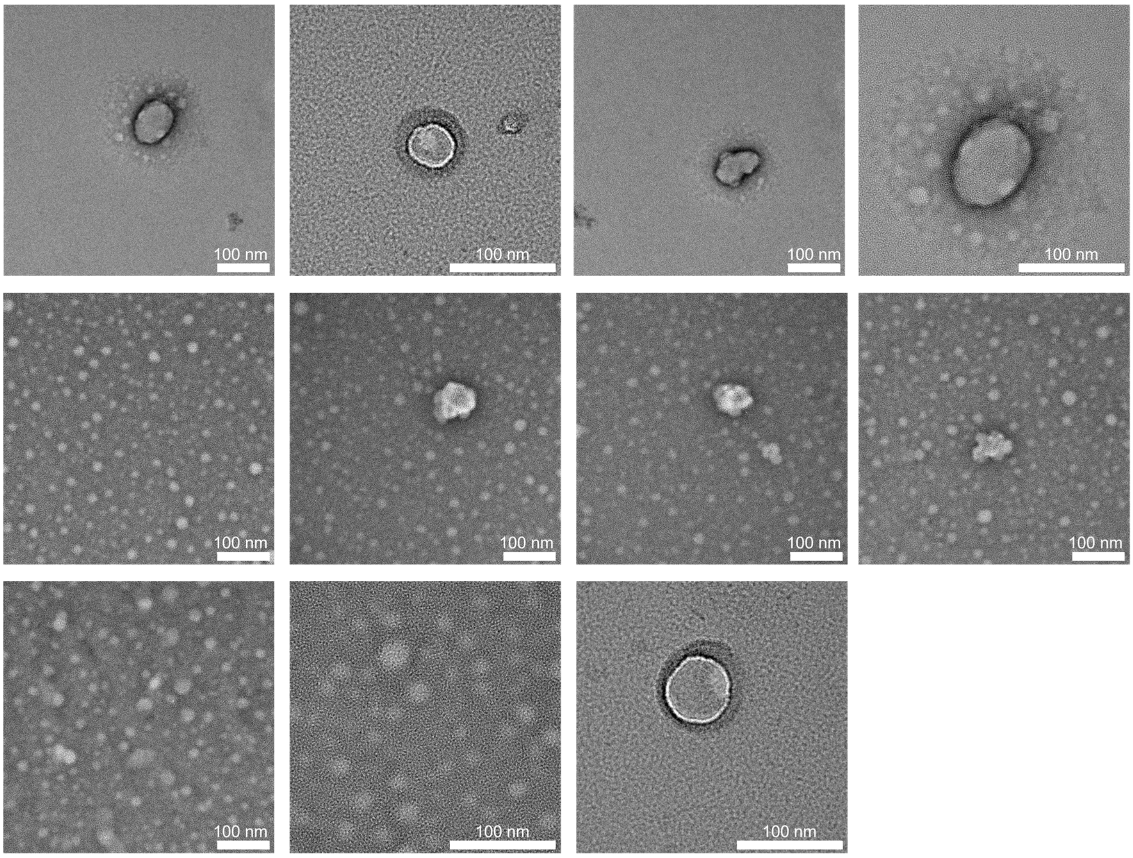

Fig. S24: TEM images of **Aβ** from 100 μM solutions, which were irradiated with a 3W 405 nm LED prior to sample preparation. Scale bar: 100 nm.

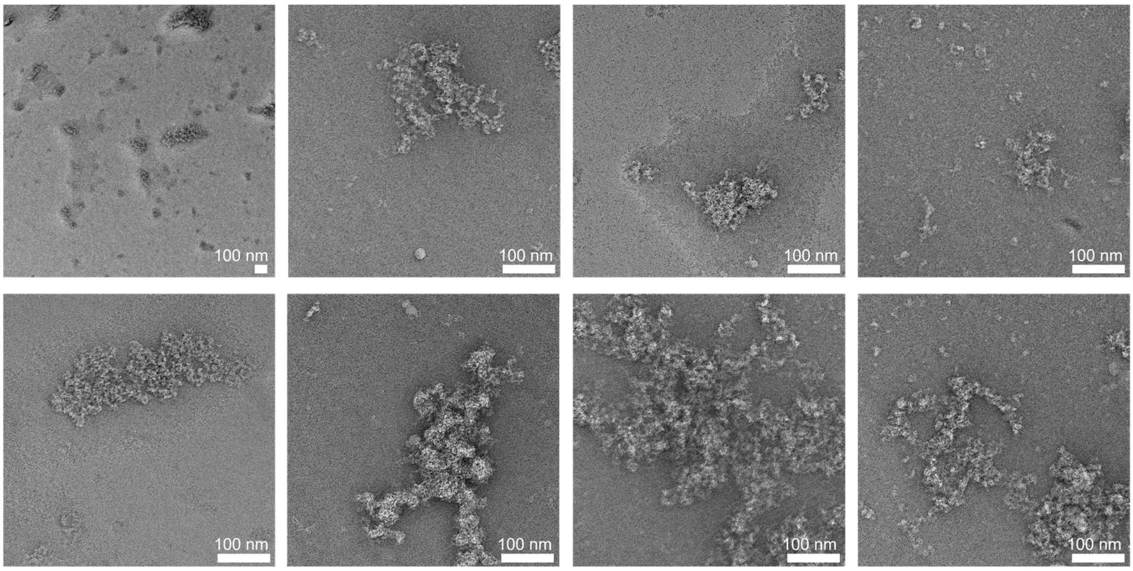

Fig. S25: TEM images of **Aα:TCP** (100:25 μM) mixtures. Scale bar: 100 nm.

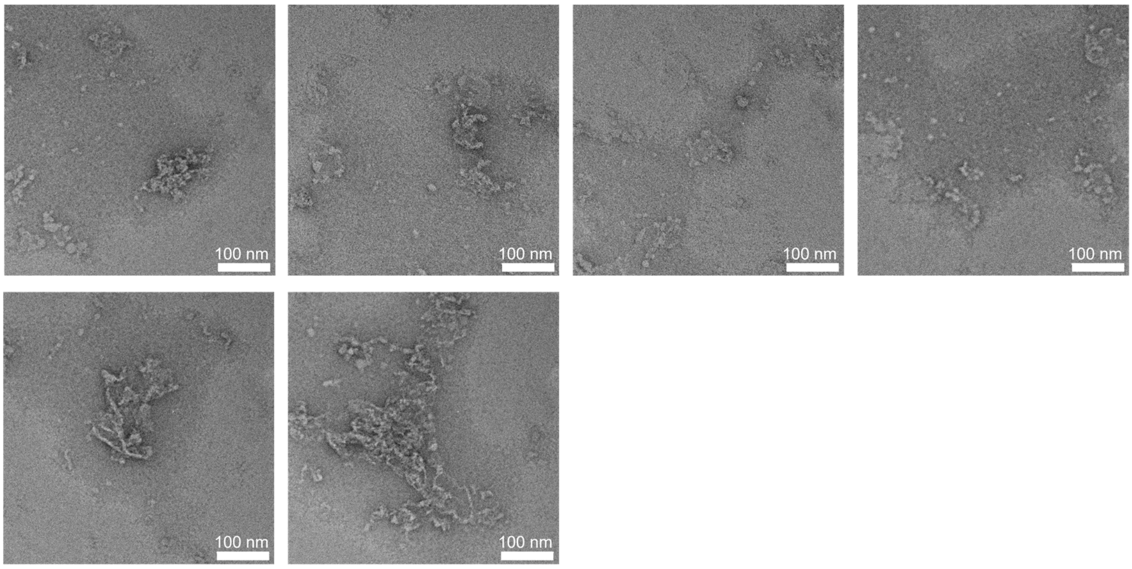

Fig. S26: TEM-images of **Aβ:TCP** (100:25 μM) mixtures. Scale bar: 100 nm.

5. Photophysical Investigation

UV/vis spectroscopy

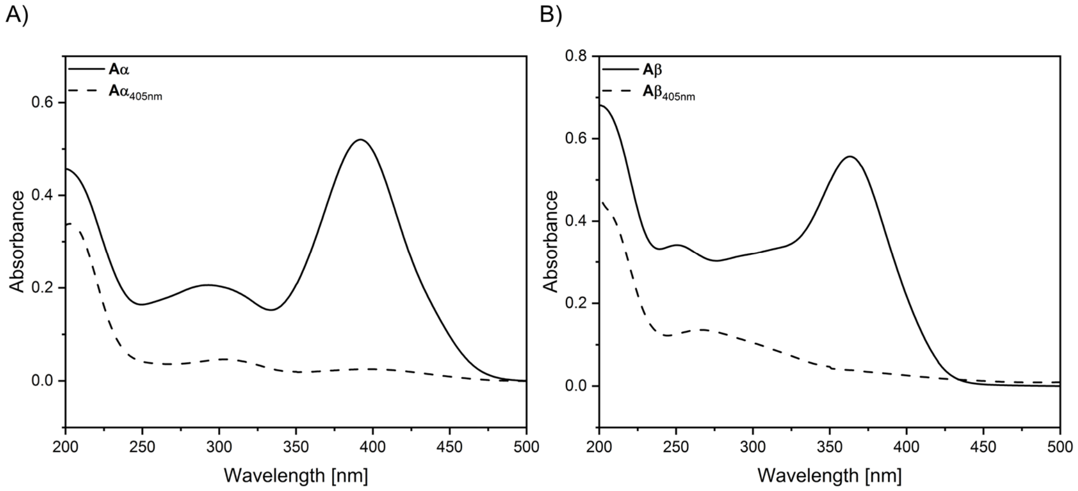

Fig. S27: UV/vis spectra of **Aα** (25 µM), **Aβ** (35 µM), and respective samples with a 3W 405 nm LED irradiated samples in water.

Fluorescence spectroscopy

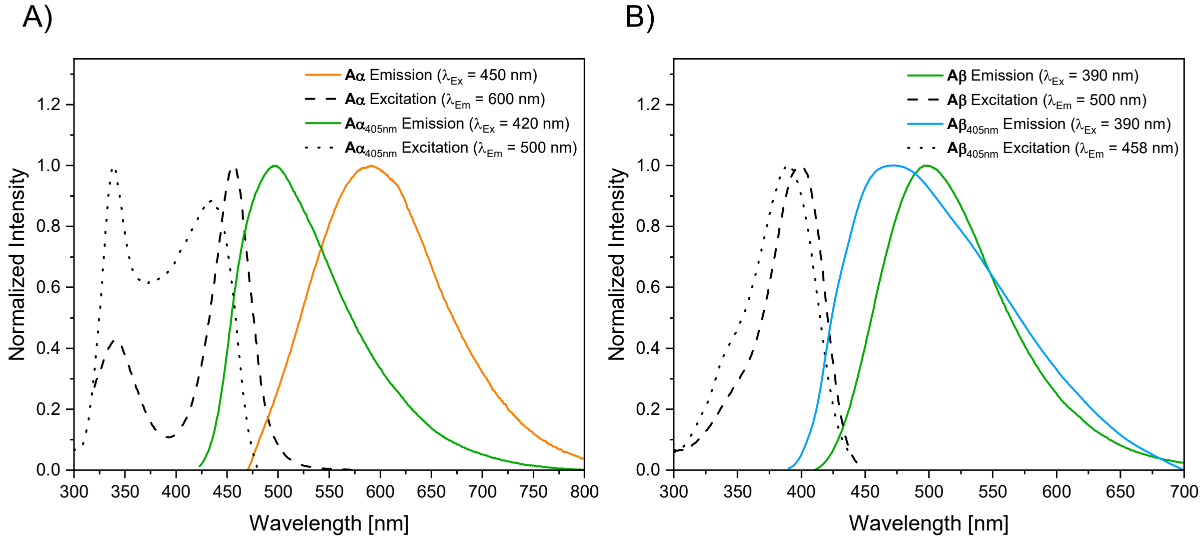

Fig. S28: Normalized fluorescence and excitation spectra of **Aα** (A), **Aβ** (B) and respective irradiated (3W 405 nm LED) samples at 100 µM in water.

We additionally determined the absolute quantum yields (Φ_L) of **Aα** and **Aα**405nm (see Tab. S1).

Tab. S1: Measured Φ_L for the **Aα** amphiphile (30 µM, H_2O) before and after irradiation (3 W 405 nm LED).

**Compound**	Φ_L ± 0.02
**Aα**	≤0.02
**Aα**405nm	≤0.02

Static Quenching

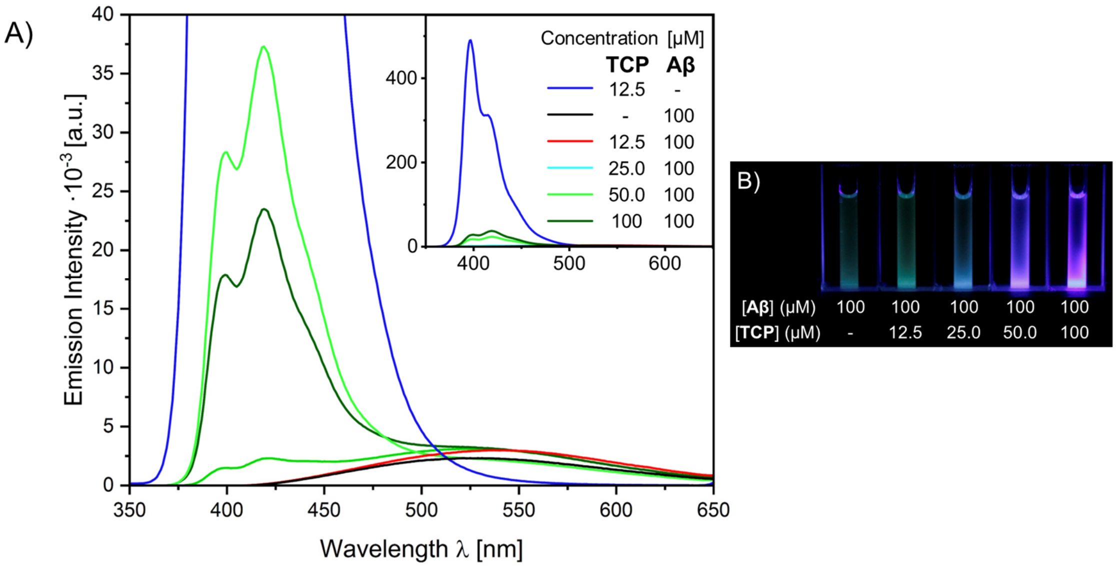

Fig. S29: (A) Fluorescence emission spectra of unimolecular samples of **TCP** and **Aβ** in water, as well as 1:8/1:4/1:2/1:1 mixtures at $\lambda_{Ex,\mathbf{TCP}}$ = 333 nm. (B) Photographs of **Aβ** and **Aβ**:**TCP** mixtures in water under UV-light (365 nm).

To investigate the binding of **TCP** to the GCP unit of the amphiphiles in detail, we carried out lifetime measurements as well as fluorescence emission spectra with increasing concentrations of the amphiphile **Aα**. The determined values are listed in the following table.

Tab. S2: Emission intensity I of the maximum of **TCP** (100 µM, H_2O) at different concentrations of **Aα** (30 – 90 µM) in a.u. as well as the lifetime τ in ns. For the Stern-Volmer-Plot, the values were referenced to the respective value for **TCP** without the amphiphile (I_0, τ_0).

c(**Aα**) [µM]	I [a.u.]	I_0/I	τ [ns]	τ_0/τ
0	1296615	1.000	5.887	1.000
30	515169	2.517	5.885	1.000
45	224973	5.763	5.811	1.013
60	119873	10.817	5.733	1.027
75	90832	14.275	5.705	1.032
90	67781	19.129	5.761	1.022

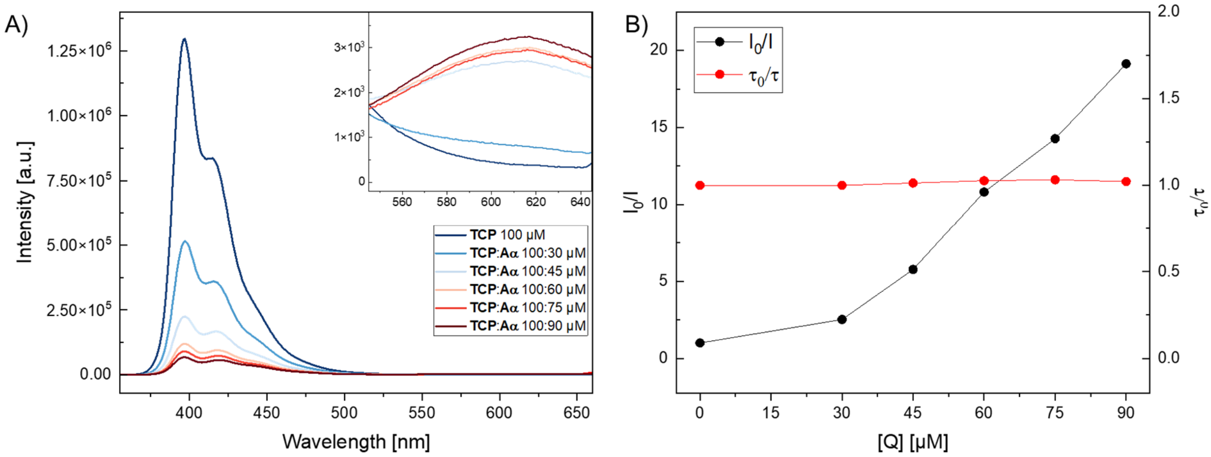

Fig. S30: A) Fluorescence emission spectra of **TCP** ($\lambda_{Ex,\mathbf{TCP}}$ = 333 nm) recorded at a fixed **TCP** concentration with increasing concentrations of **Aα** in water. The inset shows an expansion at ca. 600 nm for the emission of **Aα**. B) The intensity decrease of the **TCP** emission (black) was translated into a Stern-Volmer plot as well as measured lifetimes (red, see below) of **TCP** for the different concentrations of the quencher [Q] (**A**α).

Lifetime measurements

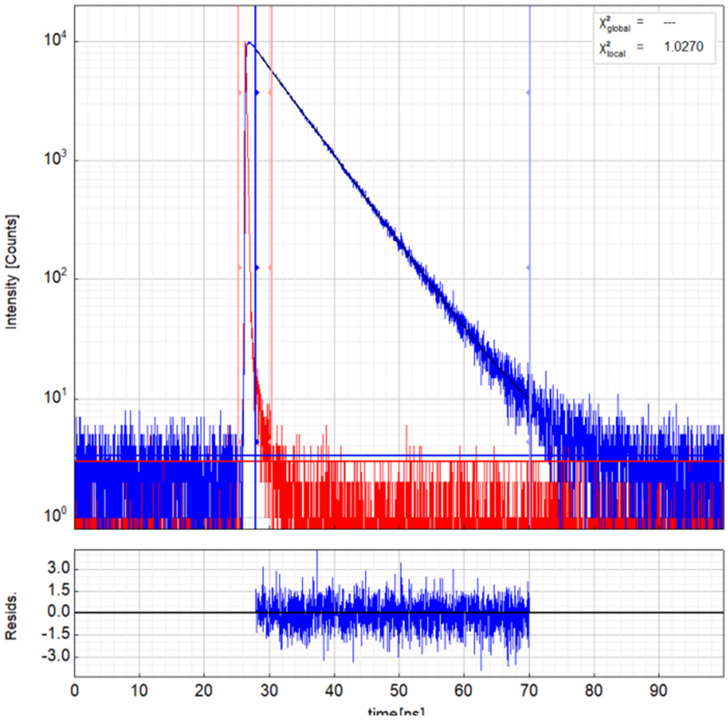

Parameter	Value	Δ	δ
A_1[kCnts/Chnl]	11.157	±0.032	0.3%
τ_1[ns]	5.887	±0.012	0.2%
I_1[kCnts]	2 627.1	±4.8	0.2%
A_{Rel1}[%]	100.0	---	---
I_{Rel1}[%]	100.0	---	---
$Bkgr_{Dec}$[kCnts]	0.0034	±0.0006	17%
$Bkgr_{IRF}$[Cnts/Chnl]	3.0000	---	---
$Shift_{IRF}$[ps]	0.0000	---	---
τ_{AvInt}[ns]	5.887	±0.012	0.2%
τ_{AvAmp}[ns]	5.887	±0.012	0.2%

Fig. S31: Raw time-resolved photoluminescence decay of **TCP** (100 µM, H_2O) in the solid-state (blue) with instrumental response function in red (left), including the residuals (λ_{ex} = 373 nm, λ_{em} = 415 nm); fitting parameters including pre-exponential factors and confidence limits (right).

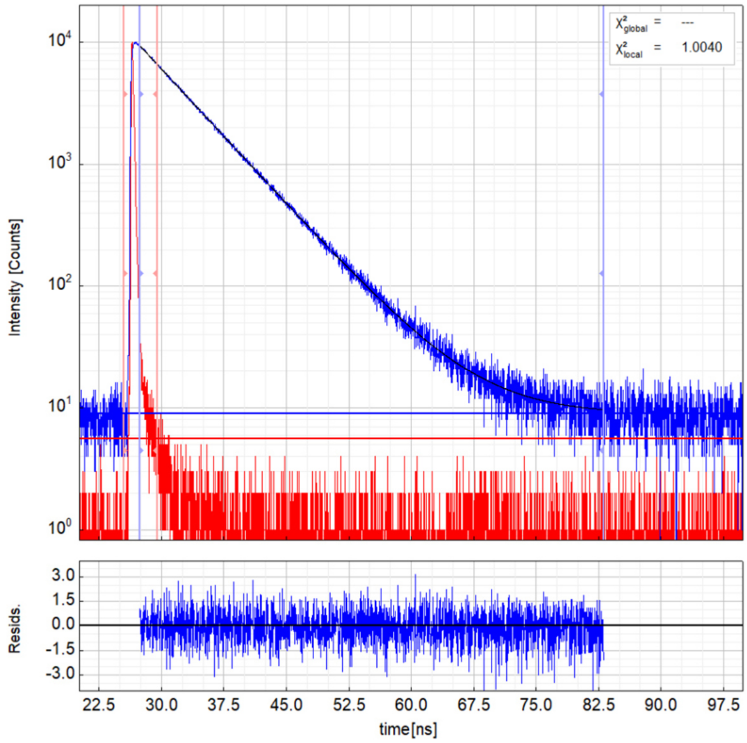

Parameter	Value	Δ	δ
A_1[kCnts/Chnl]	10.94	±0.57	5.1%
τ_1[ns]	5.885	±0.014	0.2%
I_1[kCnts]	2 580	±140	5.1%
A_{Rel1}[%]	100.0	---	---
I_{Rel1}[%]	100.0	---	---
$Bkgr_{Dec}$[kCnts]	0.0090	±0.0004	3.9%
$Bkgr_{IRF}$[Cnts/Chnl]	5.6	±6.9	122%
$Shift_{IRF}$[ps]	150	±340	237%
τ_{AvInt}[ns]	5.885	±0.014	0.2%
τ_{AvAmp}[ns]	5.885	±0.014	0.2%

Fig. S32: Raw time-resolved photoluminescence decay of **TCP:Aα** (100:30 µM, H_2O) in the solid-state (blue) with instrumental response function in red (left), including the residuals (λ_{ex} = 373 nm, λ_{em} = 415 nm); fitting parameters including pre-exponential factors and confidence limits (right).

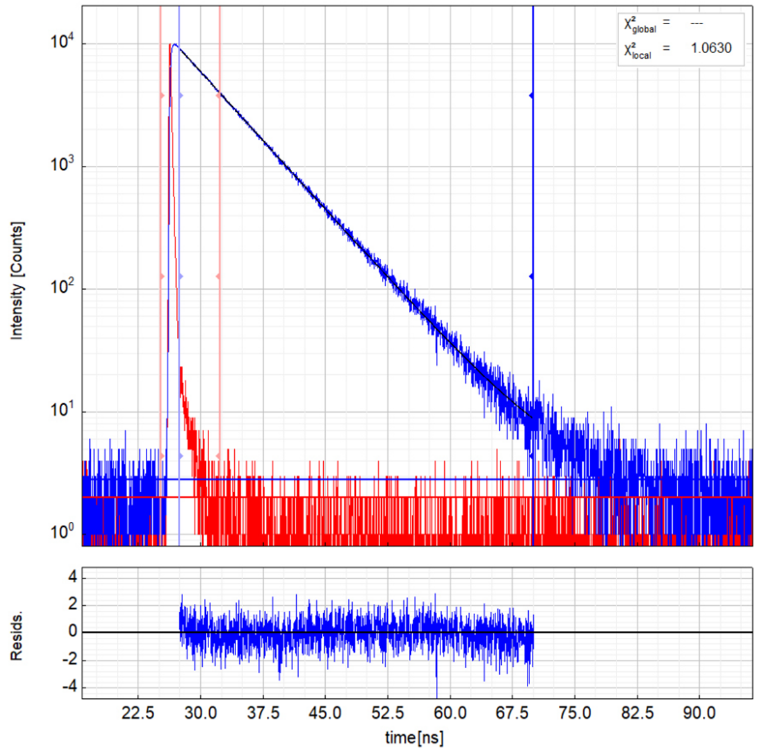

Parameter	Value	Δ	δ
A_1[kCnts/Chnl]	11.042	±0.035	0.3%
τ_1[ns]	5.811	±0.013	0.2%
I_1[kCnts]	2 566.1	±5.0	0.2%
A_{Rel1}[%]	100.0	---	---
I_{Rel1}[%]	100.0	---	---
$Bkgr_{Dec}$[kCnts]	0.0029	±0.0006	20%
$Bkgr_{IRF}$[Cnts/Chnl]	2.0000	---	---
$Shift_{IRF}$[ps]	0.0000	---	---
τ_{AvInt}[ns]	5.811	±0.013	0.2%
τ_{AvAmp}[ns]	5.811	±0.013	0.2%

Fig. S33: Raw time-resolved photoluminescence decay of **TCP:Aα** (100:45 µM, H_2O) in the solid-state (blue) with instrumental response function in red (left), including the residuals (λ_{ex} = 373 nm, λ_{em} = 415 nm); fitting parameters including pre-exponential factors and confidence limits (right).

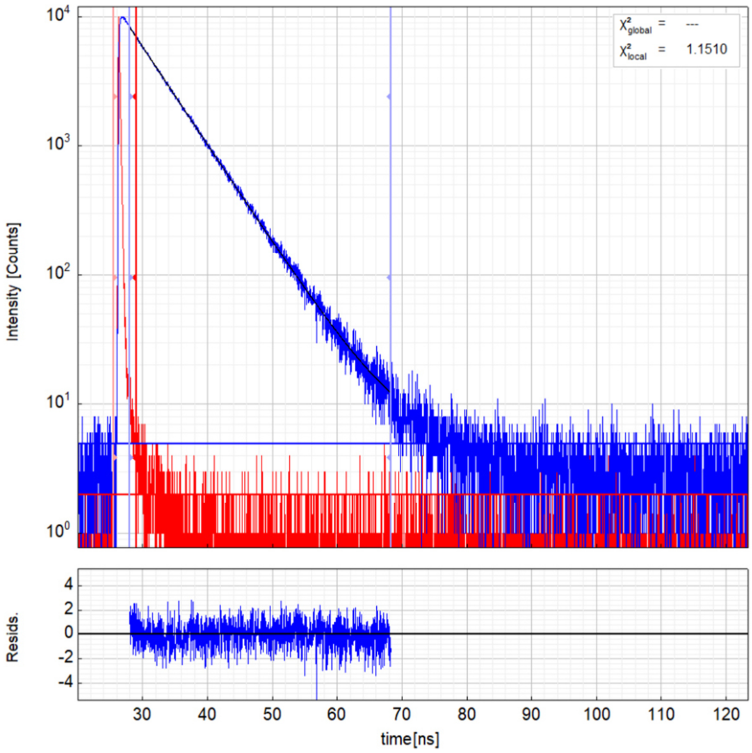

Parameter	Value	Δ	δ
A_1[kCnts/Chnl]	10.960	±0.041	0.4%
τ_1[ns]	5.733	±0.015	0.3%
I_1[kCnts]	2 512.9	±5.0	0.2%
A_{Rel1}[%]	100.0	---	---
I_{Rel1}[%]	100.0	---	---
$Bkgr_{Dec}$[kCnts]	0.0050	±0.0007	14%
$Bkgr_{IRF}$[Cnts/Chnl]	2.0000	---	---
$Shift_{IRF}$[ps]	0.0000	---	---
τ_{AvInt}[ns]	5.733	±0.015	0.3%
τ_{AvAmp}[ns]	5.733	±0.015	0.3%

Fig. S344: Raw time-resolved photoluminescence decay of **TCP:Aα** (100:60 µM, H_2O) in the solid-state (blue) with instrumental response function in red (left), including the residuals (λ_{ex} = 373 nm, λ_{em} = 415 nm); fitting parameters including pre-exponential factors and confidence limits (right).

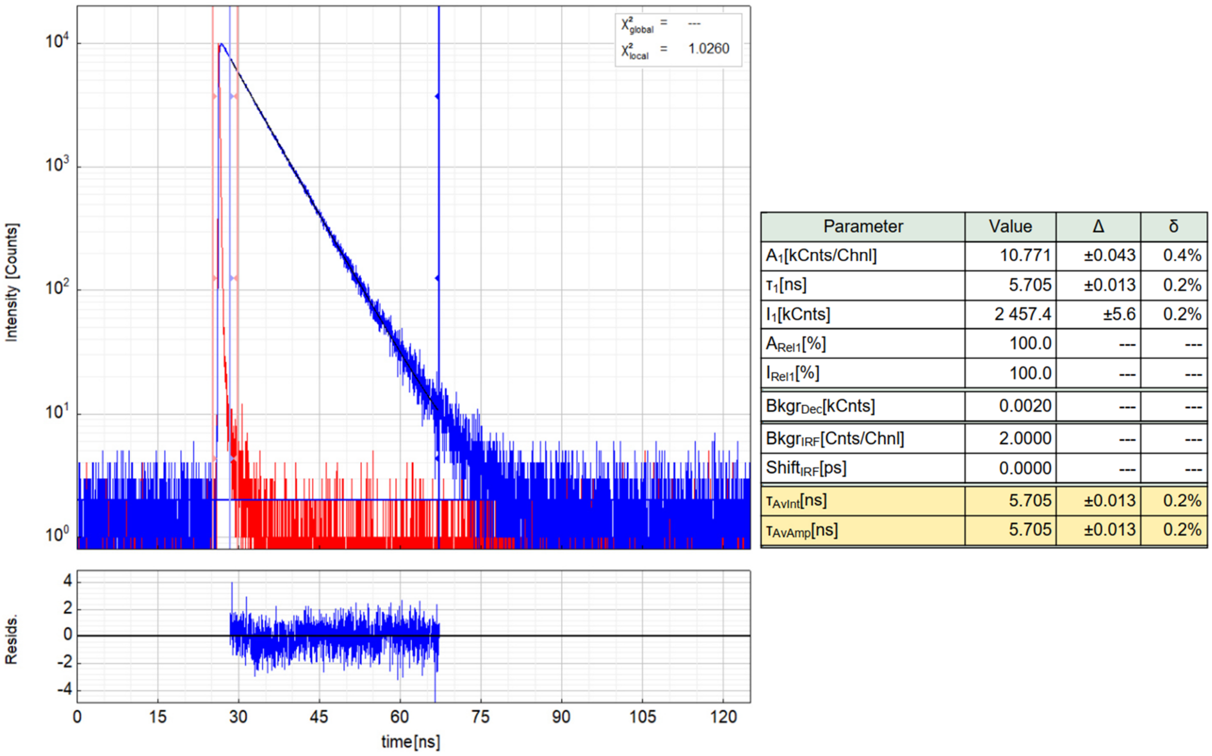

Parameter	Value	Δ	δ
A_1[kCnts/Chnl]	10.771	±0.043	0.4%
τ_1[ns]	5.705	±0.013	0.2%
I_1[kCnts]	2 457.4	±5.6	0.2%
A_{Rel1}[%]	100.0	---	---
I_{Rel1}[%]	100.0	---	---
$Bkgr_{Dec}$[kCnts]	0.0020	---	---
$Bkgr_{IRF}$[Cnts/Chnl]	2.0000	---	---
$Shift_{IRF}$[ps]	0.0000	---	---
τ_{AvInt}[ns]	5.705	±0.013	0.2%
τ_{AvAmp}[ns]	5.705	±0.013	0.2%

Fig. S35: Raw time-resolved photoluminescence decay of **TCP:Aα** (100:75 µM, H_2O) in the solid-state (blue) with instrumental response function in red (left), including the residuals (λ_{ex} = 373 nm, λ_{em} = 415 nm); fitting parameters including pre-exponential factors and confidence limits (right).

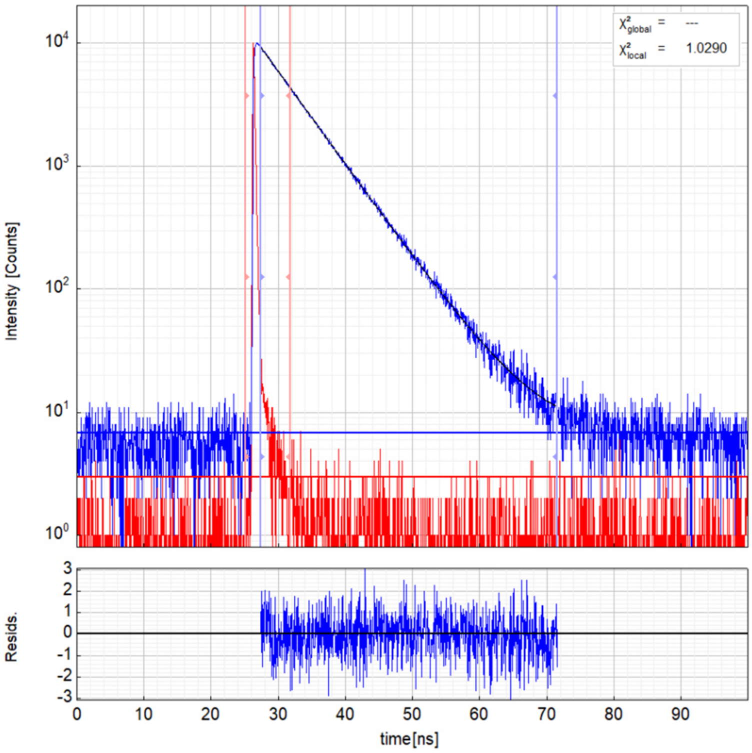

Parameter	Value	Δ	δ
A_1[kCnts/Chnl]	11.144	±0.047	0.4%
τ_1[ns]	5.761	±0.018	0.3%
I_1[kCnts]	1 283.9	±3.7	0.3%
A_{Rel1}[%]	100.0	---	---
I_{Rel1}[%]	100.0	---	---
$Bkgr_{Dec}$[kCnts]	0.0068	±0.0009	12%
$Bkgr_{IRF}$[Cnts/Chnl]	3.0000	---	---
$Shift_{IRF}$[ps]	0.0000	---	---
τ_{AvInt}[ns]	5.761	±0.018	0.3%
τ_{AvAmp}[ns]	5.761	±0.018	0.3%

Fig. S35: Raw time-resolved photoluminescence decay of **TCP:Aα** (100:90 μM, H_2O) in the solid-state (blue) with instrumental response function in red (left), including the residuals (λ_{ex} = 373 nm, λ_{em} = 415 nm); fitting parameters including pre-exponential factors and confidence limits (right).

6. Photoreaction Experiments

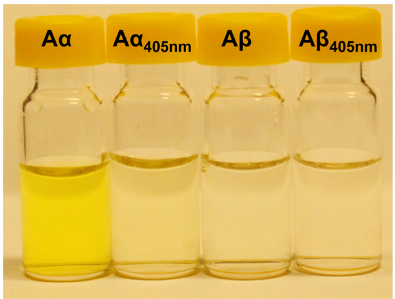

Fig. S36: 100 µM samples in daylight, before and after irradiation with light (3W 405 nm LED).

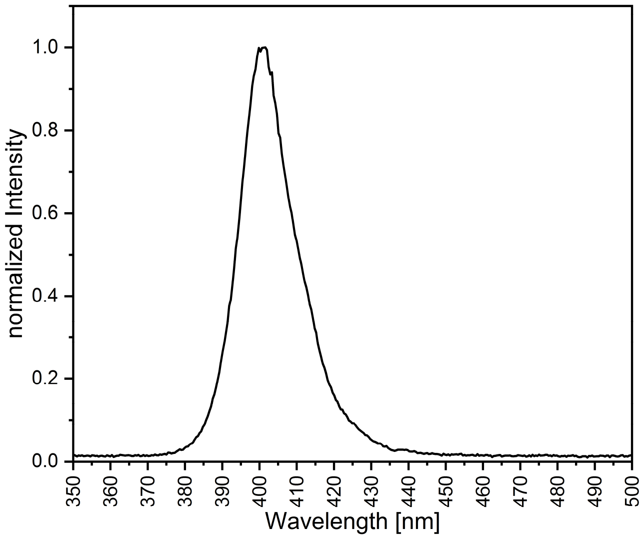

Fig. S37: Intensity spectrum of the used 3W 405 nm LED (Star-UV405-03-00-00 Highpower UV LED (405 nm) by Roschwege) at 700 mA.

NMR-Sample preparation

A sample of the amphiphile (1.5 mg, 1.7 µmol) was dissolved in 3 mL of MQ-H_2O in a 10 mL vial with a stir bar and irradiated with a 3W 405 nm LED for 30 minutes under continuous stirring. Afterwards, one equivalent NaOD (1.7 µmol) from a D_2O solution was added, mixed, and the sample transferred into a round-bottom flask for freeze-drying. The dried sample was then dissolved in 600 µL Acetone-d_6 and transferred into an NMR tube for further experiments.

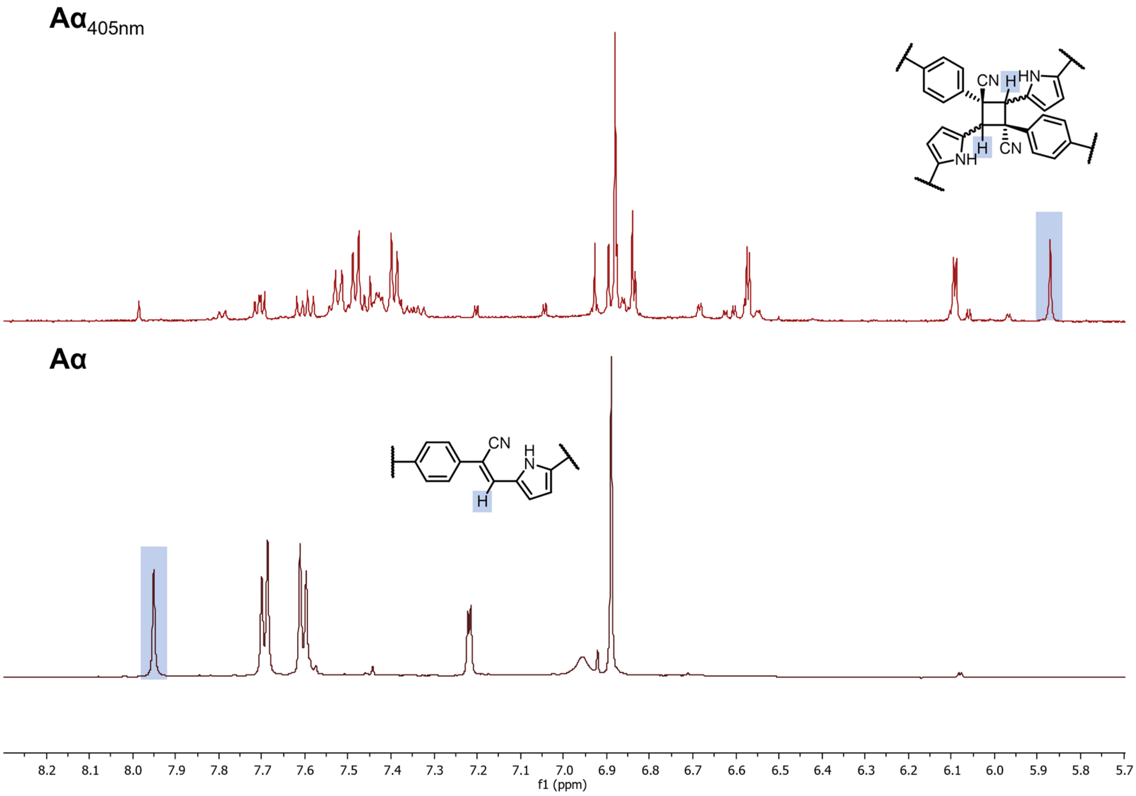

Fig. S38: ^{1}H-NMR spectra of the obtained photoproduct after irradiation of a 0.1 mM **Aα**-solution with 405 nm light for 30 min and **Aα** in Acetone-d_6 (600 MHz). Both compounds were deprotonated with 1.0 eq. NaOD with respect to the guanidinium groups, and freeze-dried prior to sample preparation. Highlights show the positions of cyclobutane protons of **Aα$_{405nm}$**, as well as the stilbene proton, for the *Z*-configuration of **Aα**.

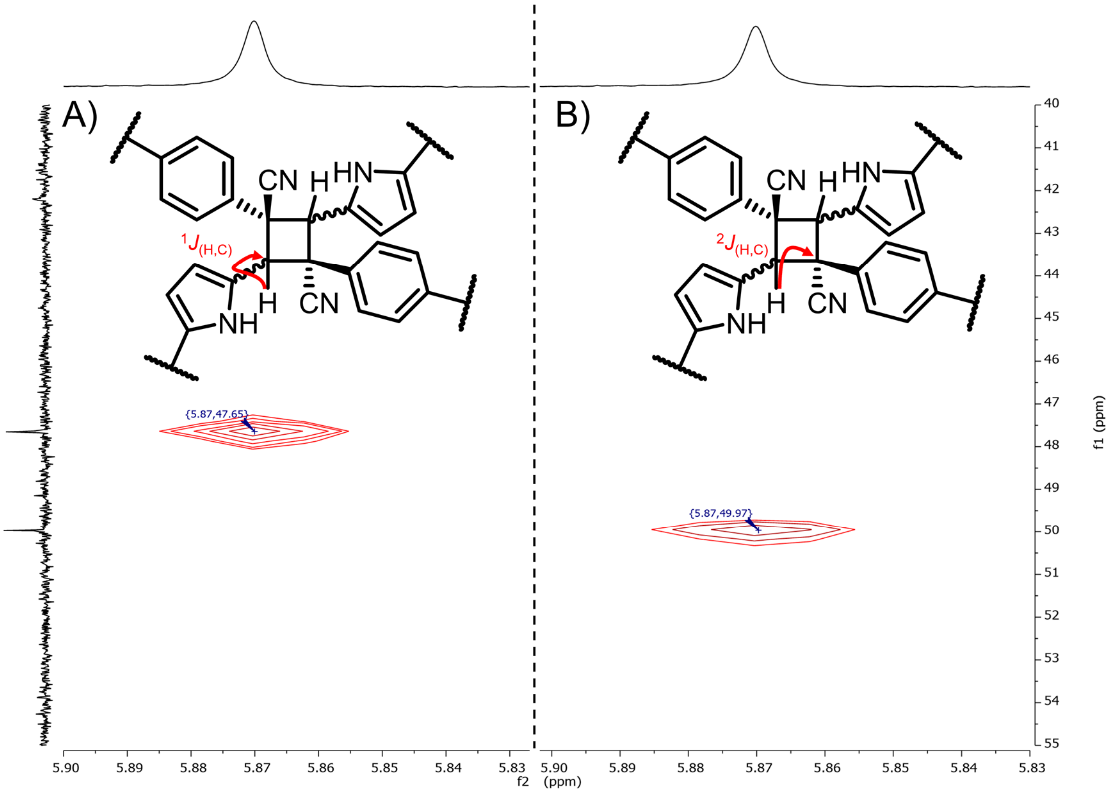

Fig. S39: (A) HSQC and (B) HMBC 2D spectra of the obtained photoproduct from irradiating a 0.1 mM **Aα** solution with 405 nm light for 30 minutes (Acetone-d_6, 600 MHz). Both spectra show a correlation between the cyclobutane proton and the two individual cyclobutane carbon atoms.

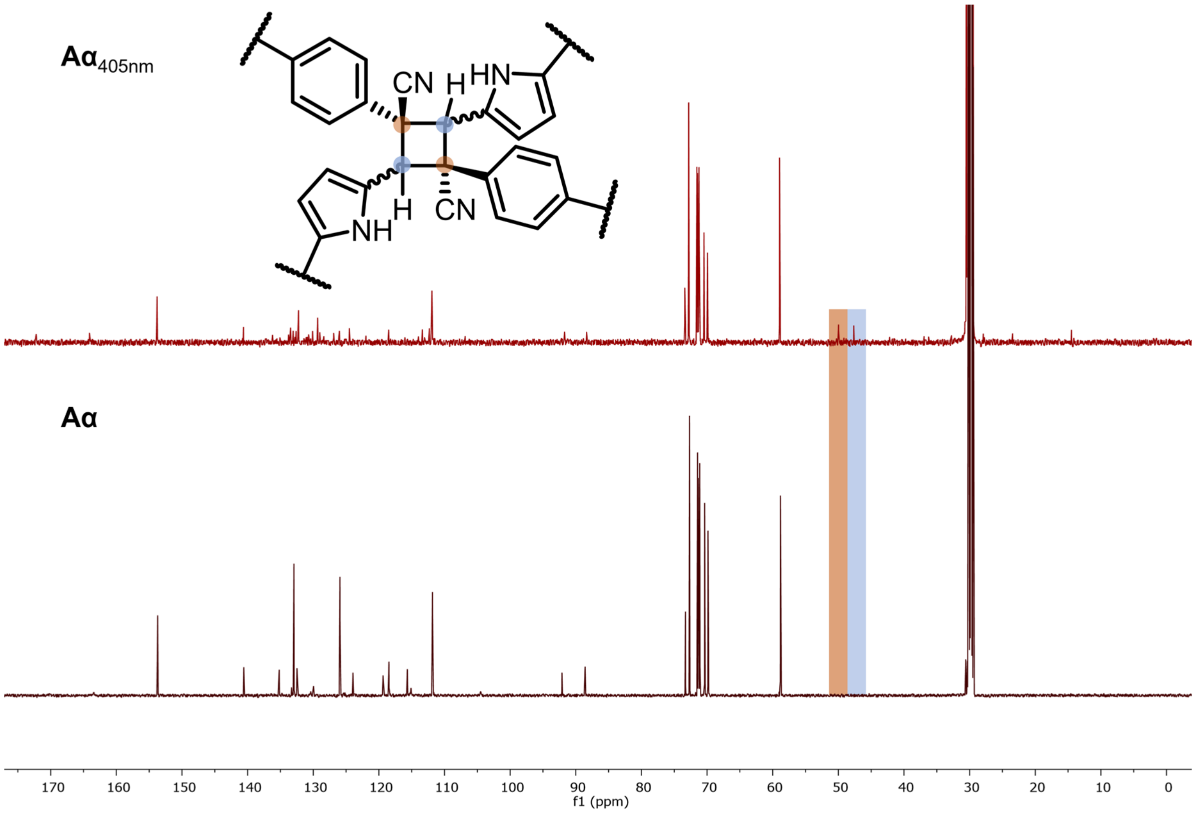

Fig. S40: ^{13}C-NMR spectra of **Aα** and its photoproduct were obtained after 30 minutes of irradiation with 405 nm light in Acetone-d_6 (600 MHz). Both compounds were deprotonated with 1.0 eq. NaOD relative to the guanidinium groups and freeze-dried before sample preparation. The newly formed cyclobutane carbon signals, which are not present in the starting material, are highlighted.

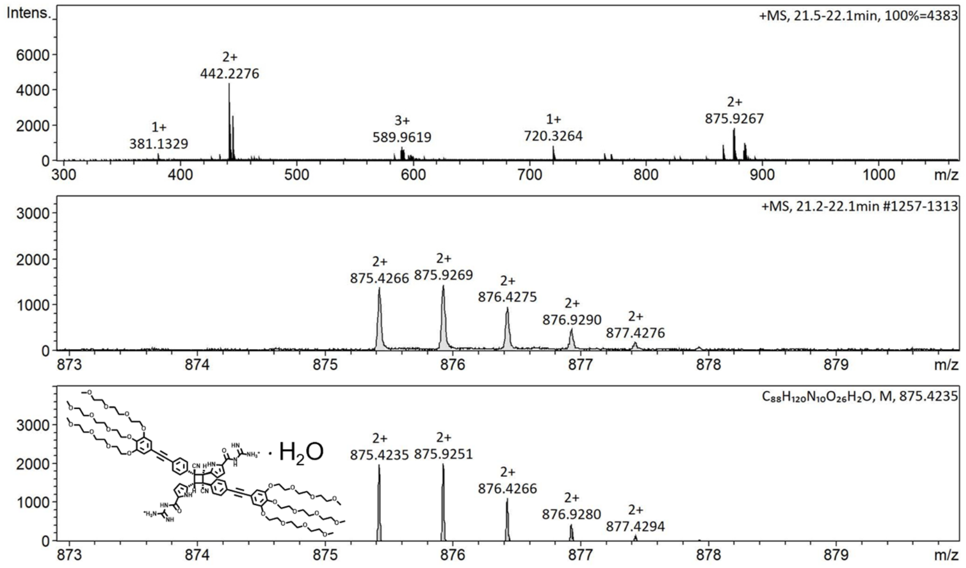

Fig. S41: HPLC-MS (ESI+) spectra at retention time 21.5 – 22.1 min of an originally 100 µM solution of **Aα** after irradiation with 405 nm light for 5 minutes. Top: overview spectrum, middle: detailed spectrum, bottom: simulated spectrum based on the sum formula of the postulated photoproduct-water adduct.

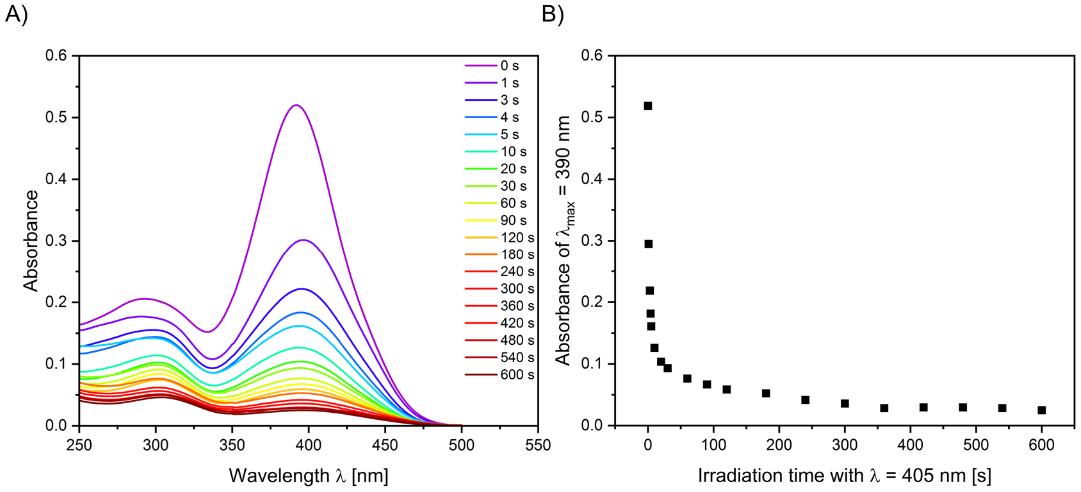

Fig. S42: (A) UV/vis spectra of **Aα** (100 µM) after increasing exposure time with 405 nm light under continuous stirring. (B) Plot of the maximum absorbance against irradiation time.

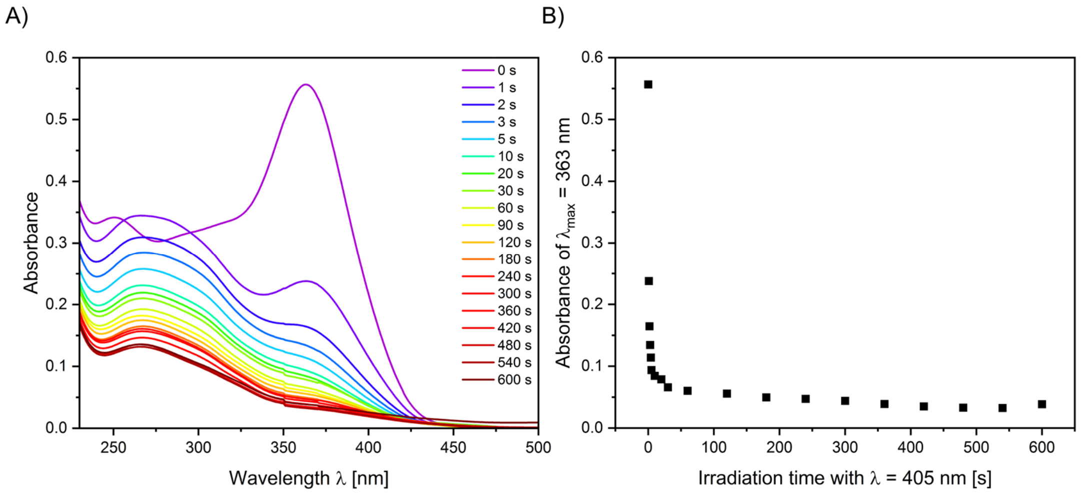

Fig. S43: (A) UV/vis spectra of **Aβ** (100 µM) after increasing exposure time with 405 nm light under continuous stirring. (B) Plot of the maximum absorbance against irradiation time.

7. Cytotoxicity and Cell permeability

Cell culture

If not stated otherwise, cells from the human cervical carcinoma line HeLa Kyoto (HeLa) were cultivated in DMEM growth medium (Invitrogen) containing 10% FBS (Gibco) and 1% Anitbiotic-Antimycotic (Gibco) at 37° C, 5% CO_2 and 90% relative humidity.

Compound preparation

Compounds **Aα** and **Aβ** were prepared in 10 mM stock solutions from MQ water. Photo-reacted samples **$A\alpha_{405nm}$** and **$A\beta_{405nm}$** were irradiated at 10 mM using a 405 nm UV LED from a distance of 1 cm, focusing the light on the 100 µL solution for 30 min. The solution was shaken every 5 minutes. The stock solutions were kept in the dark afterwards.

Cytotoxicity

Cytotoxicity was determined by MTS Cell Proliferation Assay (CellTiter 96® AQueous One, Promega). 1 x 10^6 HeLa cells were seeded in 96-well plates and incubated with different concentrations of the compounds (0.01 µM – 100 µM) in DMEM for 24 h. To determine the viability of the cells, we added 20 µL of the MTS tetrazolium compound (CellTiter 96® AQeous One Solution Reagent) to each well. MTS is reduced to colored formazan in living cells. After incubating for 1 h at 37 °C, the absorption of formazan was measured at 490 nm, which is directly proportional to the number of living cells per well, using a GloMax®-Multi plate reader (Promega). Data is shown as the mean of three replicates ± standard deviation. IC_{50} values were determined using a dose-response fit and are shown ± standard error of the mean (Origin).

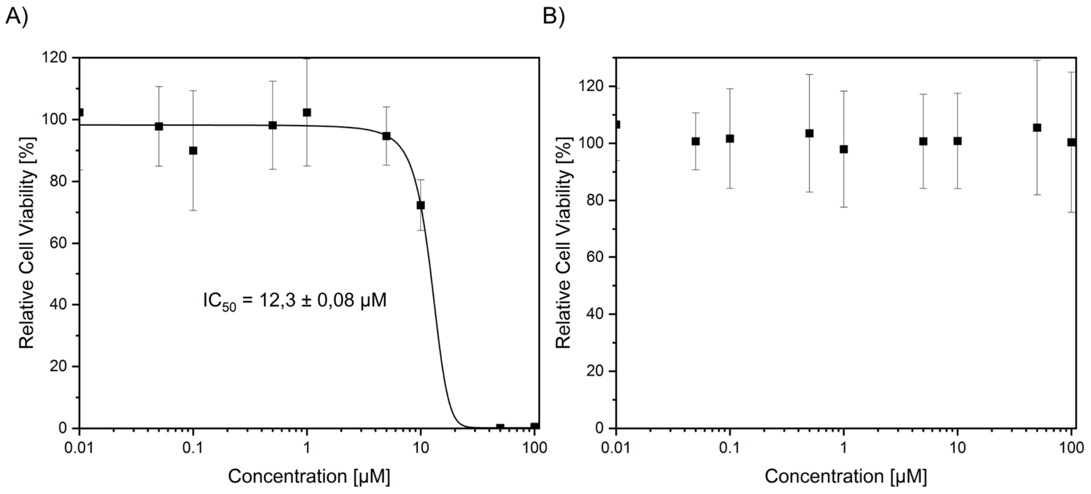

Fig. S44: Toxicity study showing the viability of HeLa cells at different concentrations of (A) **Aα** and (B) **Aα$_{\mathbf{405nm}}$**.

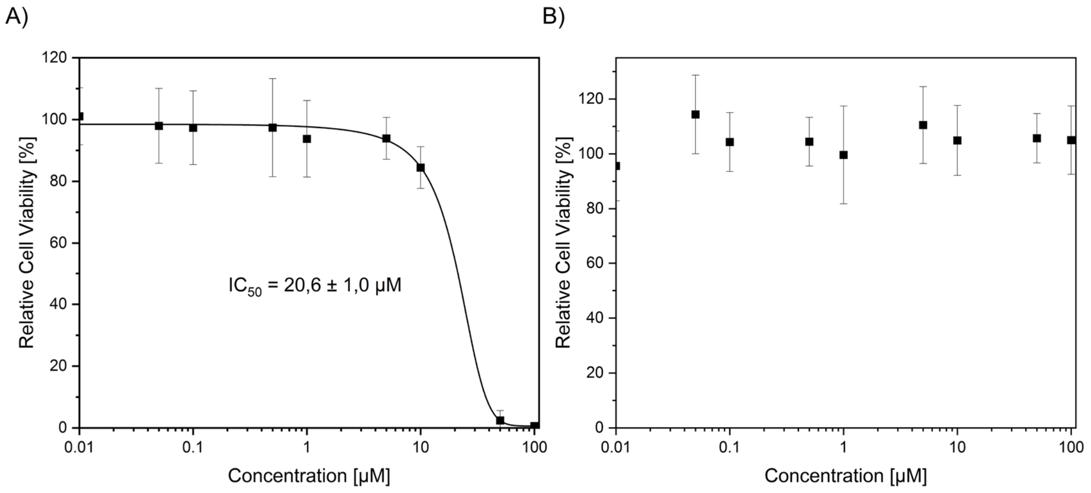

Fig. S45: Toxicity study showing the viability of HeLa cells at different concentrations of (A) **Aβ** and (B) **Aβ$_{\mathbf{405nm}}$**.

Cell permeability

Cell permeability was tested by incubating HeLa cells at two different concentrations, 10 µM and 35 µM, which are below and above the CAC for both non-irradiated compounds. After 24 h, the samples were imaged with a Leica SP8X Falcon confocal laser scanning microscope (Leica) using a HC PL APO 20x/0.75 CS2 objective (Leica). The compounds were excited using a 405 nm diode laser at 0.5% intensity. Images were generated using OMERO[8] and equal contrasts for the 405 nm channel. Automated image analysis was performed with CellProfiler (version 4.2.6).[9] Briefly, cells were detected with the Run Omnipose plugin for CellProfiler using the cyto2_omni model and their locations were used as a mask to detect vesicular structures inside the cells only.[10] Vesicular structures were detected based on Otsu three-classes thresholding (intensity). The mean sizes were measured in pixels and converted to µm^2. The pipeline is available upon request.

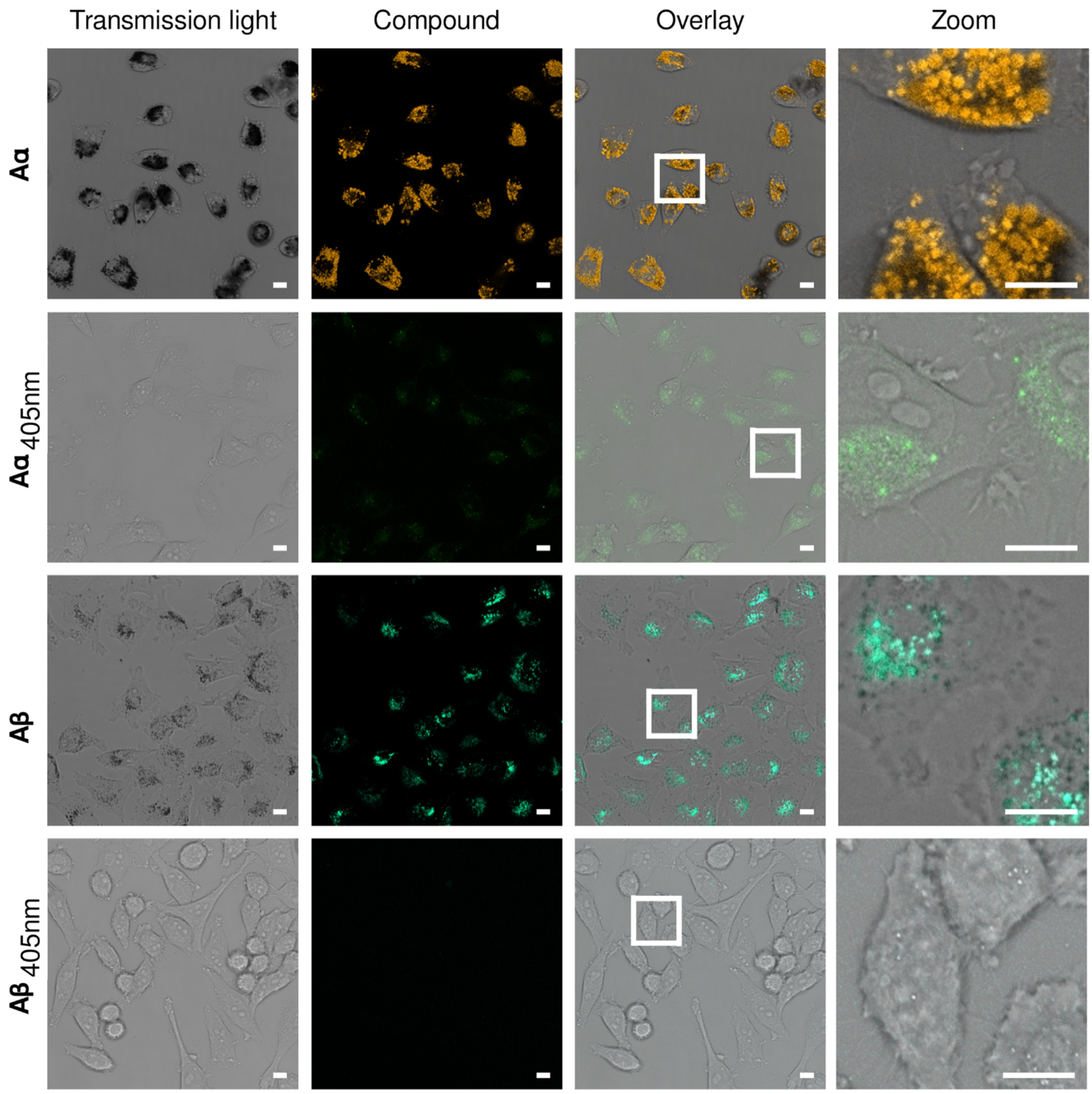

Fig. S46: Confocal images of HeLa cells after incubation with irradiated and non-irradiated amphiphiles at 10 µM concentration. Scale bar: 10 µm.

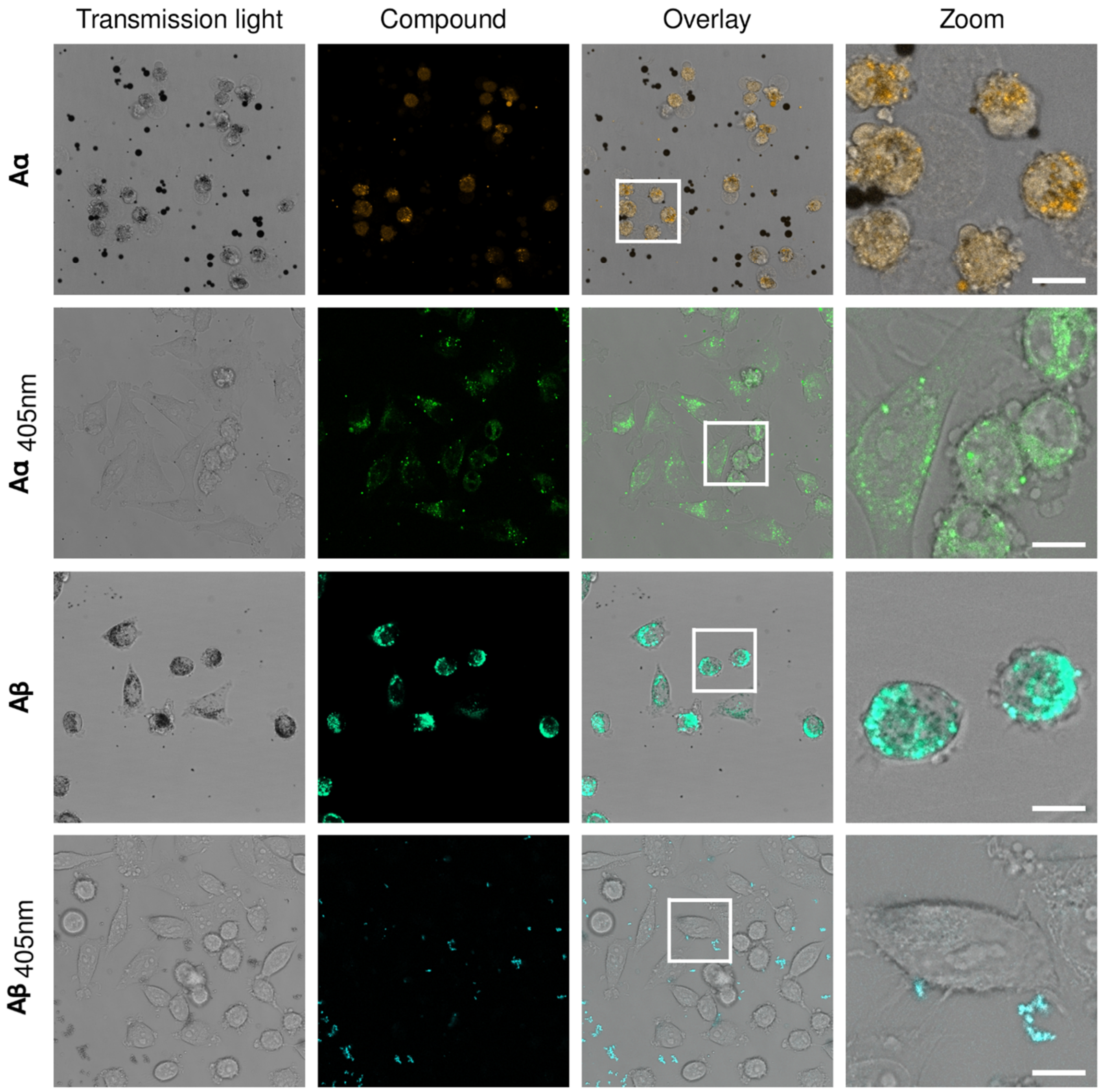

Fig. S47: Confocal images of HeLa cells after incubation with (previously irradiated) amphiphiles at 35 μM concentration. Scale bar: 10 μm.

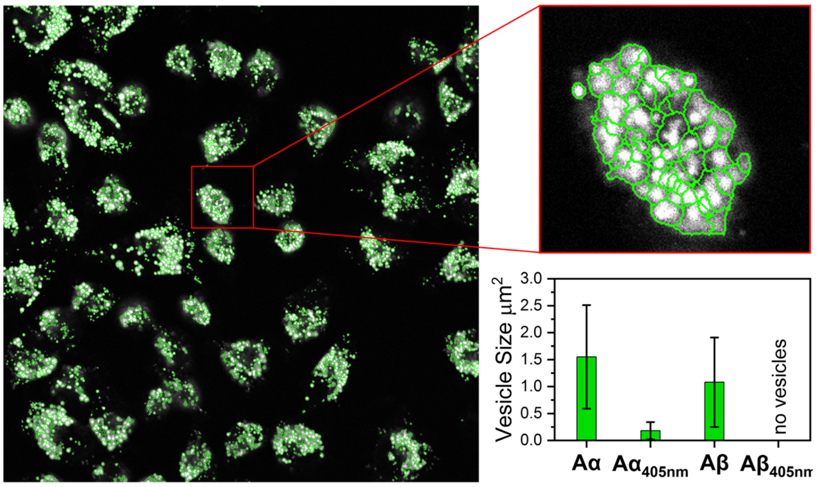

Fig. S48: Visual representation of automated image analysis with CellProfiler and mean sizes of compound-bearing vesicles.

8. Literature

[1] C. Schmuck, L. Geiger, *J. Am. Chem. Soc.* **2005**, *127*, 10486-10487.

[2] C. Schmuck, V. Bickert, M. Merschky, L. Geiger, D. Rupprecht, J. Dudaczek, P. Wich, T. Rehm, U. Machon, *Eur. J. Org. Chem.* **2008**, 324-329.

[3] J. Liang, J. Zhang, L. Zhu, A. Duarandin, V. G. Young Jr, N. Geacintov, J. W. Canary, *Inorg. Chem.* **2009**, *48*, 11196-11208.

[4] D. Zych, A. Slodek, D. Matuszczyk, S. Golba, *Eur. J. Inorg. Chem.* **2018**, *2018*, 5117-5128.

[5] W. Wu, Y. Jing, D. Zhang, X. Yan, R. Liang, Z. Lu, B. Ji, *Synthesis* **2022**, *54*, 403-410.

[6] Y.-L. Huang, D.-C. Zhong, L. Jiang, Y.-N. Gong, T.-B. Lu, *Inorg. Chem.* **2017**, *56*, 705-708.

[7] U. Vogeli, W. Von Philipsborn, *Org. Magn. Reson.* **1975**, *7*, 617-627.

[8] C. Allan, J.-M. Burel, J. Moore, C. Blackburn, M. Linkert, S. Loynton, D. MacDonald, W. J. Moore, C. Neves, A. Patterson, *Nature methods* **2012**, *9*, 245-253.

[9] D. R. Stirling, M. J. Swain-Bowden, A. M. Lucas, A. E. Carpenter, B. A. Cimini, A. Goodman, *BMC Bioinformatics* **2021**, *22*, 1-11.

[10] K. J. Cutler, C. Stringer, T. W. Lo, L. Rappez, N. Stroustrup, S. Brook Peterson, P. A. Wiggins, J. D. Mougous, *Nature methods* **2022**, *19*, 1438-1448.
